# Modeling solubility and acid–base properties of some amino acids in aqueous NaCl and (CH_3_)_4_NCl aqueous solutions at different ionic strengths and temperatures

**DOI:** 10.1186/s40064-016-2568-8

**Published:** 2016-06-30

**Authors:** Clemente Bretti, Ottavia Giuffrè, Gabriele Lando, Silvio Sammartano

**Affiliations:** Dipartimento di Scienze Chimiche, Biologiche, Farmaceutiche ed Ambientali, Università di Messina, Viale F. Stagno d’Alcontres 31, 98166 Messina, Italy

**Keywords:** Amino acids, Modeling, Protonation constants, Thermodynamics, Weak complexes, Solubility

## Abstract

New potentiometric experiments have been performed in NaCl and in (CH_3_)_4_NCl media, to determine the protonation constants, the protonation enthalpy changes and the solubility of six natural α-amino acids, namely Glycine (**Gly**), Alanine (**Ala**), Valine (**Val**), Leucine (**Leu**), Serine (**Ser)** and Phenylalanine (**Phe**). The aim of the work is the rationalization of the protonation thermodynamics (log $K_{\rm i}^{\rm H}$, solubility and $\Delta {H}_{\text{i}}^{0}$) in NaCl, determining recommended, tentative or provisional values in selected experimental conditions and to report, for the first time, data in a weak interacting medium, as (CH_3_)_4_NCl. Literature data analysis was performed selecting the most reliable values, analyzed together with new data here reported. Significant trends and similarities were observed in the behavior of the six amino acids, and in some cases it was possible to determine common parameters for the ionic strength and temperature dependence. In general, the first protonation step, relative to the amino group, is significantly exothermic (average value is $\Delta {H}_{1}^{0}$ = −44.5 ± 0.4 kJ mol^−1^ at infinite dilution and T = 298.15 K), and the second, relative to the carboxylate group, is fairly close to zero ($\Delta {H}_{2}^{0}$ = −2.5 ± 1.6, same conditions). In both cases, the main contribution to the proton binding reaction is mainly entropic in nature. For phenylalanine and leucine, solubility measurements at different concentrations of supporting electrolyte allowed to determine total and specific solubility values, then used to obtain the Setschenow and the activity coefficients of all the species involved in the protonation equilibria. The values of the first protonation constant in (CH_3_)_4_NCl are lower than the corresponding values in NaCl, due to the weak interaction between the deprotonated amino group and (CH_3_)_4_N^+^. In this light, differences between the protonation functions in NaCl and (CH_3_)_4_NCl were used for the quantification of the stability of the weak [(CH_3_)_4_N^+^–L^−^] complexes that resulted log *K* = −0.38 ± 0.07 as an average value for the six amino acids.

**Electronic supplementary material:**

The online version of this article (doi:10.1186/s40064-016-2568-8) contains supplementary material, which is available to authorized users.

## Background

As well known, α-amino acids (αAA) are fundamental for any form of life (Amend and Helgeson 1997), being the building block of proteins and nutrients. Many fundamental information about biochemistry and nutrition of AA may be found in a recent dedicated textbook (Wu 2013). From a nutritional point of view, the twenty natural α-amino acids may be divided into essential, EAA (AA that must be introduced in the organism by an adequate diet), conditionally essential, CEAA (AA that *normally* can be synthesized in adequate amounts by the organisms, but must be provided in the diet in certain *conditions*) and non-essential, NEAA (may be synthesized by the organisms), but this classification depends on species, development stage, physiological status, environmental factors and disease (Wu 2013).

According to Frausto da Silva and Williams, (Frausto da Silva and Williams 2001) the twenty α-amino acids may be divided in four classes on the basis of the residue linked to the α-carbon atom, namely: charged hydrophilic (Lysine, Arginine, etc.), intermediate hydrophilic (Serine, Histidine, etc.), hydrophobic (Leucine, Valine, Alanine, Phenylalanine, etc.) and structural amino acids (Glycine, Proline). The relative percentages of each amino acid in a protein largely influences its properties, such as folded structure, hydration and mobility (Frausto da Silva and Williams 2001). Being the α-carbon atom a chiral center all the α-amino acids have two enantiomers (except glycine), but the L-stereoisomer is predominant in nature. However, D-AA also exist in animals, micro-organisms, and plants (Friedman 1999).

In natural waters, amino acids may reach 30–40 % of the dissolved organic nitrogen (DON), (Tuschall and Brezonik 1980), and they may be found as excretion products of living organisms, as hydrolysis product of oligo-peptides or formed by transamination reaction (De Stefano et al. 2000). Amino acids participate in the synthetic and respiratory metabolism of the organisms and represent, together with sugars, an important food and energy source for heterotrophic microorganisms (Campbell and Goldstein 1972). Buffle et al. (1988) measured the percentages of combined amino acids, after hydrolysis, with respect to dry organic matter, finding 33, 55 to 75, 55.4 and 13 to 26 % for marine zoo- and phytoplankton, bacteria, mollusks, and macrophytes, respectively. It was found that marine organisms may uptake glycine and α-alanine even when salinity decreases to about 12. At lower levels, the animals continue to survive, but free amino acids may not be acquired, probably due to reduced osmotic regulation processes (Stephens 1972). Similarly, increasing the salinity, the availability of free amino acids decreases for their complexation with macro components of seawater (e.g. Ca^2+^). This discussion emphasizes the importance of speciation studies in natural fluids, in fact the knowledge of reliable stability constants, solubility data and accurate thermodynamic information is strictly necessary to solve analytical problems in industrial, environmental and biological fields, such as optimisation of purification and separation processes (Berthon 1995; Nagai et al. 2008).

In the literature, protonation constants and, to a lesser extent, enthalpy changes for most amino acids are available in the most important database (Martell et al. 2004; May and Murray 2001; Pettit and Powell 2001) and in some compilations that have been dedicated to this class of molecules [see, e.g., (Berthon et al. 1984; Kiss et al. 1991; Pettit 1984; Sóvágó et al. 1993)]. Generally, most of the data refer to 298.15 K and low ionic strength, mainly in aqueous NaCl, KCl, NaNO_3_, KNO_3_, NaClO_4_. On the contrary, data in tetralkylammonium salts aqueous solutions are, to our knowledge, absent except those reported by De Stefano et al. (1995).

The protonation of amino acids heavily depends on the presence of salts in solution. This dependence is specific, even if at low ionic strength values is very similar for the majority of 1:1 electrolytes. However, a marked difference is observed in tetraalkylammonium salts, depending on the lipophylic character of these cations, that in aqueous solution tend to reduce the surface area accessible to water molecules. As known solvation-desolvation processes influence protonation thermodynamic parameters of amino acids in aqueous solutions of tetralkylammonium salts. Moreover, as described in our previous papers, unprotonated amino group can interact with (CH_3_)_4_N^+^ and carboxylate anion with Na^+^ forming weak complexes (Berto et al. 2012; Bretti et al. 2013, 2014a, 2015; Crea et al. 2016).

Protonation constants of glycine and alanine in mixed ethanol–water (Doğan et al. 2002; Jabbari and Gharib 2010) and dioxane-water mixtures (Köseoğlu et al. 2000) confirmed that increasing the percentage of the organic solvents the more basic protonation constant tends to increase quite linearly. Microscopic and tautomeric protonation constants of alanine and valine were obtained by Gharib et al. (2015). A detailed collation of literature data is given as Additional file 1.

**Scheme 1:**
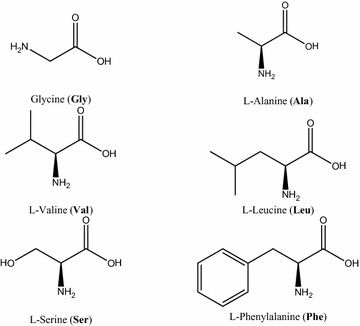
Structures of amino acids

In this paper different types of L-amino acids, have been studied (see Scheme 1), namely Glycine (**Gly**), Alanine (**Ala**), Valine (**Val**), Leucine (**Leu**), Serine (**Ser)** and Phenylalanine (**Phe**).

The aim of this study is the determination of suggested data sets for the protonation thermodynamic parameters (proton binding, enthalpy and solubility) of **Gly**, **Ala**, **Val**, **Leu**, **Ser**, **Phe**, in NaCl_aq_ and (CH_3_)_4_NCl_aq_ at different ionic strengths and temperatures, by means of an analysis of literature and new experimental data. The dependence of protonation constants and solubility of amino acids on ionic strength and temperature was quite neglected for some amino acids in the literature. The need of reliable thermodynamic data at different ionic strengths and temperatures is recognized particularly for applications to real matrices.

## Experimental section

### Chemicals

The six α-amino acids investigated in this work were Sigma-Aldrich products. Solutions were prepared by weighing, and the purity, determined by alkalimetry, resulted to be better than 99 %. Tetramethylammonium chloride was purified as recommended by Perrin (Perrin et al. 1966). HCl, NaOH and (CH_3_)_4_NOH aqueous solutions were prepared by diluting concentrated Fluka ampoules. NaOH and (CH_3_)_4_NOH aqueous solutions were checked against potassium phthalate, HCl aqueous solutions were standardized against sodium carbonate. Sodium chloride aqueous solutions were prepared by weighing pure salt (Fluka, p.a.), pre-dried in an oven at 408 K. Analytical grade water (R = 18 MΩ cm) and grade A glassware were used to prepare all the solutions.

### Apparatus and procedure for potentiometric and solubility measurements

Titrations were performed using a Model 809 Metrohm Titrando. Emf was measured with a combined glass electrode (Metrohm 6.032.100) to a precision of ±0.15 mV, calibrated by using a Gran plot, applied to data from HCl/NaOH (or (CH_3_)_4_NOH) titration. The reliability of the calibration in alkaline conditions was checked by computing the value of p*K*_w_. Titrant was added by a 800 Dosino by Metrohm, and the precision of the titrant delivery was ±0.003 cm^3^. Temperature was always maintained at 298.1 ± 0.1 K by means of water circulation. Magnetic stirring was used throughout. Presaturated nitrogen gas was bubbled through the solutions in order to remove dissolved oxygen and carbon dioxide. The procedure adopted for the potentiometric measurements is reported elsewhere (Cigala et al. 2012). Solubility measurements were performed for **Leu** and **Phe** as described in previous works (Bretti et al. 2012). Briefly, saturated solutions were prepared in thermostatted vessels by adding an excess of the ligand to a solution containing NaCl or (CH_3_)_4_NCl at different concentrations, ranging between 0.1 and 5.0 mol dm^−3^ (or 3.0 mol dm^−3^ in the case of (CH_3_)_4_NCl). Preliminary measurements of conductivity indicated that equilibrium was reached after stirring the solution for ca. 24 h. The solid was removed using 0.45 μm MFMillipore filters, and the supernatant solution was analyzed by means of potentiometric titration with standard NaOH or (CH_3_)_4_NOH solutions. To minimize the systematic errors, several independent experiments were carried out for each salt concentration.

### Analysis of the data

The computer program ESAB2M (De Stefano et al. 1987) was used to refine parameters of acid base titrations including (1) standard electrode potential *E*^0^, (2) junction potential coefficients *j*_a_, (the deviation from Nernstian behaviour is defined as *E*_j_ = *j*_a_ [H^+^]), (3) an alkali purity parameter, (4) protonation constants of ligands at a specific ionic strength. Protonation and solubility data at different ionic strengths or salt concentrations were fitted, using appropriate equations, with the software LIANA (De Stefano et al. 1997).

The formation constants of weak complexes were computed using the program ES2WC (De Robertis et al. 1987).

The equilibrium constants are given according to the equilibria (charges omitted for simplicity):

1 $$ \hbox{H} + \hbox{H}_{{\rm i} - 1} \hbox{L} = \hbox{H}_{\rm i} \hbox{L}\quad \hbox{K}_{\rm i}^{\rm H} = \left[{\hbox{H}_{\rm i} \hbox{L}} \right]/\left[\hbox{H} \right] \cdot \left[{H_{{\rm i} - 1} L} \right] $$

1a $$ \hbox{iH} + \hbox{L} = \hbox{H}_{\rm i} \hbox{L}\quad\beta_{i}^{\rm H} = \left[{\hbox{H}_{\rm i} \hbox{L}} \right]/\left[\hbox{H} \right]^{\rm i} \cdot \left[\hbox{L} \right] $$

and

2 $$ \hbox{jM} + \hbox{iH} + \hbox{L} = \hbox{M}_{j} \hbox{H}_{i} \hbox{L} \quad \beta_{\rm ji}^{\rm M} = [\hbox{M}_{\rm j} \hbox{H}_{\rm i} \hbox{L}]/[\hbox{M}]^{\rm j} \cdot [\hbox{H}]^{\rm i} \cdot [\hbox{L}] $$

where M can be Na^+^ or (CH_3_)_4_N^+^.

Protonation constants, concentrations and ionic strengths are determined on the molar concentration scale, but SIT parameters are based on the molal concentration scale. Molar to molal conversion was performed using the appropriate density values for the different ionic media (De Stefano et al. 1994). Throughout the paper, uncertainties are given as ±95 % confidence interval (CI). Distribution diagrams were drawn using HySS computer program (Alderighi et al. 1999) (Additional file 2).

### Dependence of equilibrium constants on ionic strength and temperature

The dependence of protonation constants, expressed as in Eq. (1a), on ionic strength can be expressed in terms of activity coefficients as in Eq. (3)

3 $$ { \log }\beta_{\text{i}}^{\text{H}} = { \log }\beta_{\text{i}}^{\text{H0}} + {\text{i}} \cdot { \log }\gamma_{{{{\text{H}}^{+}}}} + { \log }\gamma_{{\text{L}}} - { \log } \gamma_{{{{\text{H}}_{\text{i}} {\text{L}}}}} $$

where log $\beta_{\rm i}^{\rm H0}$ is the value of the protonation constants at infinite dilution and γ_Y_ is the activity coefficient of the species Y. The dependence of single ion activity coefficients on ionic strength can be expressed in terms of a simple Debye–Hückel type (DHt) equation Eq. (4)

4 $$ log\gamma_{Y} = {-} z_{Y}^{2} \cdot 0.51 \cdot I^{0.5}/\left({1 + 1.5 \cdot I^{0.5}} \right) + f\left(I \right) $$

where z_Y_ is the charge on the ion Y and *f* (*I*) is a function of ionic strength. Most simply is *f* (*I*) = *C*_i_ · *I*. When a neutral species is involved, the activity coefficient log γ is the Setschenow coefficient (*k*_*m*_) times the molal concentration of the supporting electrolyte.

When both ionic strength and equilibrium constants are expressed in the molal concentration scale, the Debye–Hückel type equation becomes the SIT (Specific ion Interaction Theory) (Brönsted 1922; Ciavatta 1980; Grenthe and Puigdomenech 1997; Guggenheim and Turgeon 1955; Scatchard 1936) approach and *f* (*I*) = Δε_i_ · *I*. The SIT theory is based on the assumption that in Eq. (4) the linear term *f* (*I*) depends on interaction between ions of opposite charge, and this can be expressed as:

5 $$ f\left(I \right) = \varSigma \varepsilon \cdot m_{\rm M,X} \approx \Delta \varepsilon_{i} \cdot I $$

where ε is the specific interaction coefficient and the sum covers the interactions between the ion under examination and all the ions (M or X) of opposite charge multiplied for the molal concentration (*m*) of the latter. For example, considering the stepwise protonation constants as in Eq. (1)

$$ \hbox{H}^{+} + \hbox{L}^{-} = \hbox{HL}^{0} \;\hbox{K}_{1}^{\rm H} $$

$$ \hbox{H}^{+} + \hbox{HL}^{0} = \hbox{H}_{2} \hbox{L}^{+} \;\hbox{K}_{2}^{\rm H} $$

The expression for Δε_i_, in NaCl, is

6 $$ \Delta \upvarepsilon_{1} = \upvarepsilon \left({\hbox{H}^{+},\hbox{Cl}^{-}} \right) + \varepsilon \left({\hbox{Na}^{+},\hbox{L}^{-}} \right){-}k_{\rm m} \left({\hbox{NaCl}} \right) $$

6a $$ \Delta \upvarepsilon_{2} = \upvarepsilon \left({\hbox{H}^{+},\hbox{Cl}^{-}} \right) + k_{m} \left({\hbox{NaCl}} \right) - \varepsilon \left({\hbox{H}_{2} \hbox{L}^{+},\hbox{Cl}^{-}} \right) $$

C_i_, Δε_i_, ε, and *k*_m_ may vary with ionic strength according to different equations. For example, in this work the following was used (Bretti et al. 2014b)

7 $$ \Delta \upvarepsilon_{\rm i} = \Delta \upvarepsilon_{\rm i}^{\infty} + (\Delta \varepsilon_{\rm i}^{0} - \Delta \varepsilon_{\rm i}^{\infty})/(I + 1) $$

The summation of the single activity coefficients of Eq. (3), as expressed in Eq. (4), leads to the general equation for the dependence of equilibrium constants on ionic strength:

8 $$ \hbox{log} K_{\rm i} = \hbox{log} K_{\rm i}^{0} -\hbox{z}^{*} \cdot 0.51 \cdot I^{0.5}/ \left({1 + 1.5 \cdot I^{0.5}} \right) + f(I ) $$

8a $$ z^{*} = \Sigma \left(\hbox{charges} \right)_{\rm reactants}^{2} -\Sigma \left( \hbox{charges} \right)_{\rm products}^{2} $$

The temperature dependence of the amino acid protonation constants was also studied by means of the well known Clarke and Glew (1966) equation:

9 $$ \log K_{T {\rm i}}^{\rm H0} = \log K_{\theta {\rm i}}^{\rm H0} + (\Delta H_{\rm i}^{0} + \Delta C_{\rm pi} \cdot (T - \theta) + \Delta \varepsilon_{\rm i}^{\prime} \cdot I) \cdot 52.23 \cdot \left({\frac{1}{\theta} - \frac{1}{T}} \right) $$

where ${\text{log}}K_{{{\uptheta  {\rm i}}}}^{\text{H0}}$ is the protonation constant ad infinite dilution (superscript “0”) and at the reference temperature, θ, log $K_{{{\text{T}  {\rm i}}}}^{\text{H0}}$ is the protonation constant at any temperature. $\Delta H_{\rm i}^{0}$ is the protonation enthalpy at infinite dilution of the ith step, $\Delta {C}_{\text{pi}}$ and $\Delta \varepsilon_{\rm i}^{\prime}$ are the temperature and ionic strength dependence parameters of $\Delta H_{\rm i}^{0}$.

Combination of Eqs. (8) and (9) leads to the fitting equation:

10 $$ \log K_{T {\rm i}}^{\rm H} = \log  K_{\theta}^{\rm H0} - z^{*} \cdot 0.51 \cdot I^{0.5}/(1 + 1.5 \cdot I^{0.5}) + \Delta \varepsilon_{\rm i} \cdot I + (\Delta H_{\rm i}^{0} + \Delta C_{\rm pi} \cdot (T - \theta) + \Delta \varepsilon_{i}^{\prime} \cdot I) \cdot 52.23 \cdot \left({\frac{1}{\uptheta} - \frac{1}{T}} \right) $$

### Solubility of α-amino acids in NaCl_(aq)_ and (CH_3_)_4_NCl_(aq)_ ionic media

The theoretical aspects of solubility measurements are well established (Brandariz 2006; Bretti et al. 2011, 2012). The total solubility (S^T^) of a ligand L is defined as the sum of the concentrations of all the species formed in the solution in equilibrium with solid(s), Eq. (11)

11 $$ S^{\text{T}} = \left[{\text{L}}^{-} \right] + \left[{\text{HL}}^{0} \right] + \left[{\text{H}}_{2} {\text{L}}^{+} \right] $$

If HL^0^ is denoted as the solubility of the neutral species (or “specific”) of the ligand, *S*^0^, and considering the stepwise protonation constants (${K}_{\text{i}}^{\text{H}}$, see hereafter) calculated in the same experimental conditions of the solubility measurements, rearrangement of Eq. (11) leads to:

12 $$ {S}^{\text{T}} = S^{ 0} \cdot \left({{1 +}\frac{1}{{{K}_{1}^{\text{H}} \cdot \left[{{\text{H}}^{+}} \right]}} + {K}_{2}^{\text{H}} \cdot \left[{{\text{H}}^{+}} \right]} \right) $$

Equation (12) is used to calculate the solubility of the neutral species once the total solubility (*S*^T^), the pH and the protonation constants are known.

In the presence of a supporting electrolyte MX, the total solubility ($\log S_{m}^{T}$) can be expressed as a function of this salt concentration (*m*_MX_) according to the following equation:

13 $$ {\text{log}}S_{{m}}^{\text{T}} = {\text{log}}S_{{m 0}}^{\text{T}} +\left({{{a}}_{\infty} + \frac{{{{a}}_{0} - {{a}}_{\infty}}}{1 + {{{{m}}_{\text{MX}}}}}} \right) \cdot {{m}}_{\text{MX}} $$

where $\log S_{m0}^{\rm T}$ is the total solubility in pure water and ${{a}}_{ 0}$ and ${{a}}_{\infty}$ are empirical parameters proposed in the past by Bretti et al. (Bretti et al. 2012). The subscript “*m*” indicates the molal concentration scale, but the molar can also be used with the appropriate conversions. According to Long and McDevit (Long and McDevit 1952), using the Setschenow constant *k*_m_ (Setschenow 1889), variation in the activity coefficient with a supporting electrolyte concentration is

14 $$ \log \gamma = \log \frac{{S_{m0}^{0}}}{{S_{m}^{0}}} = k_{m} \cdot m_{\rm MX} $$

therefore

15 $$ \log  S_{m}^{0} = \log  S_{m 0}^{0} - k_{m} \cdot m_{\rm MX} $$

The Setschenow coefficient *k*_*m*_ may vary with salt concentration similarly to the parameter “*a*” in Eq. (13). Solubility products (log *K*_S0_) can be determined by means of eq. (16):

16 $$ {\text{K}}_{\text{S0}} = \frac{{{\text{S}}^{0}}}{{{\text{K}}_{1}^{\text{H}}}} $$

### Determination of the weak complexes

In this work, weak complexes have been successfully calculated by the so-called Δlog *K*^H^ or Δp*K* method, i.e., by the differences of the apparent protonation constants of the ligands in “non-interacting” (better “very weakly interacting”) aqueous media and those in an interacting medium. In general and as in this case, for molecules containing the amino group, which forms weak species with tetraalkylammonium cations (Bretti et al. 2008; De Stefano et al. 2005), the baseline electrolyte is an alkali metal salt. A detailed description of the basic principles of this approach and some examples can be found, for example, in Berto et al. (2012), Bretti et al. (2013), Daniele et al. (2008). Briefly, it is well established that for a simple monoprotic acid (HL), the lowering effect of the apparent protonation constant in an “interacting” medium (log *K*_i_^H*^) with respect to a non-interacting (log *K*_i_^H^) can be interpreted in terms of weak complex formation between the deprotonated ligand and the cation of the supporting electrolyte:

17 $$ \log K_{\rm i}^{\rm H^{*}} = \log K_{\rm i}^{\rm H} {-}\log (1 + 10^{\log K^{\rm M}} c_{\rm M}) $$

For polyprotic ligands, a slightly more complicated calculation should be used, though it starts from a basic assumption, i.e., that the average number of protons bound to a ligand ($\bar{p}$) is fixed in given conditions, independently of its expression. This means that it can indifferently be calculated using only the apparent overall protonation constants (*β*_i_^H*^, referred to Eq. 1a)

18 $$ {{\bar{p}}}^{*} = \frac{{\sum {{\rm i}\beta}_{\text{i}}^{{{\text{H}}^{*}}} \left[{{\text{H}}^{+}} \right]^{\text{i}}}} {{{1 +}\sum {\beta}_{\text{i}}^{{{\text{H}}^{*}}} \left[{{\text{H}}^{ }} \right]^{\text{i}}}} $$

or by the effective protonation constants (*β*_0i_, *β*_0i_≡*β*_i_^H^) and the complex formation constants (Eq. 2):

19 $$ \bar{p} = \frac{{\sum {\text{i}}\beta_{\rm ji}^{\rm M} \left[ {{\text{M}}^{ + } } \right]^{\rm j} \left[ {{\text{H}}^{ + } } \right]^{i} }}{{1 + \sum \beta_{\text{ji}}^{\rm M} \left[ {{\text{M}}^{ + } } \right]^{\rm j} \left[ {{\text{H}}^{ + } } \right]^{\rm i} }} $$

the equivalence of the two expressions means that the formation constants (of weak complexes) can be calculated by minimizing the function

20 $$ U = \sum \left({\bar{p}{-}\bar{p}^{*}} \right)^{2} $$

using ES2WC (De Robertis et al. 1987). Worth mentioning is the fact that, in this kind of calculations, the molar concentration scale (in mol dm^−3^) must be used (De Robertis et al. 1987; Pytkowicz 1979a, b)

## Result and discussion

### Collection of literature data

Since in the literature there are many papers dealing with the protonation thermodynamics (equilibrium constants, enthalpy, entropy and solubility) of the amino acids under study, a critical analysis of the literature data was done. The most important reference papers are those of the IUPAC organization and references therein (Amend and Helgeson 1997; Kiss et al. 1991; Pettit 1984; Sóvágó et al. 1993), that proposed a useful method for the selection of the literature data, dividing the whole data among four groups: recommended, tentative, doubtful and rejected. For our purposes, only the data belonging to the first two groups were used. Together with the IUPAC data, worth mentioning are the equilibrium constant databases (Martell et al. 2004; May and Murray 2001; Pettit and Powell 2001), that report reference data in the molal concentration scale. Other important papers, published after the above mentioned ones, are those in refs. (Brandariz et al. 1993; Doğan et al. 2002; Gharib et al. 2015; Hamborg et al. 2007; Jabbari and Gharib 2010; Köseoğlu et al. 2000; Nagai et al. 2008). A collection of the literature data (log $K_{\rm i}^{\rm H}$ and $\Delta H_{\rm i}^{0}$) selected for the analysis in this paper is reported as Additional file 1: Tables S1–S5. Considering the amount of data listed for the protonation of glycine in the IUPAC report (Kiss et al. 1991), it was chosen to avoid the presence of a dedicated table. For the sake of simplicity, graphical comparisons of the literature and experimental data for **Ala**, **Leu**, **Phe** and **Ser** are reported at *T* = 298.15 K (see following paragraph).

### Protonation constants and solubility of α-amino acids

In this paper, new potentiometric and solubility measurements are reported at different temperatures and ionic strengths in NaCl and (CH_3_)_4_NCl for **Gly**, **Leu**, **Val**, **Ala**, **Phe** and **Ser**. For the sake of brevity, the experimental values of the protonation constants of the six amino acids are reported entirely as Additional file 1: Tables S6–S17 both in NaCl and in (CH_3_)_4_NCl aqueous media. Experimental enthalpy change values for the proton binding reactions were derived from the dependence of the protonation constants on temperature according to the well known Clarke and Glew equation (Clarke and Glew 1966; see Eq. 9), and a collection of these values is reported as Additional file 1: Tables S18−S23), together with literature values for **Ala** (Additional file 1: Table 24S), whereas those of **Gly** are reported mainly in the dedicated IUPAC report (Kiss et al. 1991).

Experimental and literature protonation constants showed similar trends, and for this reason they have been analyzed simultaneously, taking into account the reliability of the literature ones, to obtain common ionic strength and temperature dependence parameters, and a unique set of calculated protonation constants in different conditions.

**Fig. 1:**
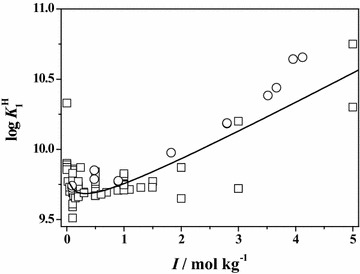
Literature (*square*) and experimental (*circle*) values of the first protonation constant of **Ala** versus ionic strength in Na^+^ media at *T* = 298.15 K with Eq. (10, *black line*)

**Fig. 2:**
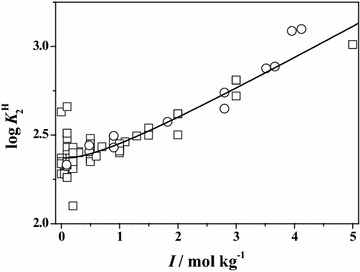
Literature (*square*) and experimental (*circle*) values of the second protonation constant of **Ala** versus ionic strength in Na^+^ media at *T* = 298.15 K with Eq. (10, *black line*)

**Fig. 3:**
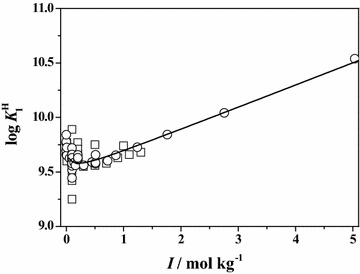
Literature (*square*) and experimental (*circle*) values of the first protonation constant of **Leu** versus ionic strength in Na^+^ media at *T* = 298.15 K with Eq. (10, *black line*)

**Fig. 4:**
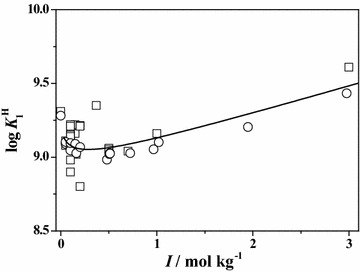
Literature (*square*) and experimental (*circle*) values of the first protonation constant of **Phe** versus ionic strength in Na^+^ media at *T* = 298.15 K with Eq. (10, *black line*)

**Fig. 5:**
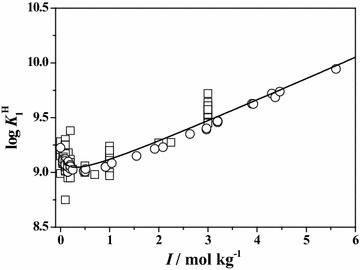
Literature (*square*) and experimental (*circle*) values of the first protonation constant of **Ser** versus ionic strength in Na^+^ media at *T* = 298.15 K with Eq. (10, *black line*)

In order to better compare and describe the scattering of the literature ($\square$) and experimental (○) data reported in this paper, some plots of both data are reported at *T* = 298.15 K. In particular, in Figs. 1 and 2, the dependence of the first and the second protonation constants of **Ala** on ionic strength is shown. The graphs relative to the first protonation constant of **Leu**, **Phe** and **Ser**, are reported in Figs. 3, 4 and 5, respectively. In all cases, although the general agreement between the literature and the experimental data is satisfactory, there is a big amount of scattered data in the ionic strength range 0 < *I*/mol kg^−1^ ≤ 1.5, whereas few data up to *I* = 5 mol kg^−1^. In the Figures, the black line represents the best fit, according to Eq. (10), obtained considering all the data.

**Fig. 6:**
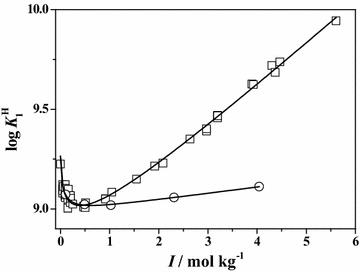
Dependence of the experimental values of **Ser** first protonation constant on ionic strength in NaCl (*square*) and (CH_3_)_4_NCl (*circle*) at *T* = 298.15 K

Data in tetramethylammonium chloride, or in other interacting medium, are not present in the literature. The trend of the experimental values of **Ser** first protonation constant in NaCl and in (CH_3_)_4_NCl is reported in Fig. 6, showing that data in the latter medium are lower than in the former and indicating that the tetramethylammonium cation interacts with deprotonated amino group, as reported recently (Bretti et al. 2013, 2015). The quantification of this interaction will be treated later.

Once literature and experimental protonation constants were established, it was chosen to perform the data analysis, with the model represented by Eq. (10), in the molal concentration scale, owing to the recommendation of the SIT model (Brönsted 1922; Ciavatta 1980; Grenthe and Puigdomenech 1997; Guggenheim and Turgeon 1955; Scatchard 1936). Therefore, the experimental and the literature protonation data, when necessary, were converted to this scale, using appropriate density values (De Stefano et al. 1994).

The results of the data analysis are reported in Table 1, together with average values of all the parameters, calculated considering the six amino acids.

**Table 1:** Ionic strength and temperature dependence parameters of protonation constants Eq. (10) at *T* = 298.15 K and at infinite dilution for l-glycine, l-alanine, l-valine, l-leucine, l-serine and l-phenylalanine in NaCl and (CH_3_)_4_NCl

Parameter	Gly	Ala	Val	Leu	Ser	Phe	Average
Log ${\text{K}}_{1}^{\text{H0}}$	9.777 ± 0.004^a^	9.912 ± 0.012^a^	9.730 ± 0.008^a^	9.777 ± 0.005^a^	9.255 ± 0.009^a^	9.257 ± 0.006^a^	9.618 ± 0.287^a^, 9.80 ± 0.08^c^
Log ${\text{K}}_{2}^{\text{H0}}$	2.329 ± 0.007	2.365 ± 0.015	2.286 ± 0.005	2.321 ± 0.007	2.162 ± 0.005	2.144 ± 0.017	2.267 ± 0.093, 2.32 ± 0.03^c^
$\Delta {\varepsilon}_{ 1}^{\infty}$ (NaCl)	0.190 ± 0.016	0.195 ± 0.012	0.189 ± 0.082	0.260 ± 0.012	0.187 ± 0.005	0.162 ± 0.047	0.197 ± 0.033
$\Delta {\varepsilon}_{ 1}^{0}$ (NaCl)	0.311 ± 0.020	0.35 ± 0.037	0.395 ± 0.068	0.354 ± 0.014	0.329 ± 0.019	0.332 ± 0.049	0.345 ± 0.029
$\Delta {\varepsilon}_{ 2}^{\infty}$ (NaCl)	0.098 ± 0.010	0.114 ± 0.007	0.089 ± 0.180	0.177 ± 0.007	0.096 ± 0.009	0.241 ± 0.053	0.136 ± 0.061
$\Delta {\varepsilon}_{ 2}^{ 0}$ (NaCl)	0.093 ± 0.023	0.091 ± 0.031	0.070 ± 0.066	0.069 ± 0.020	0.061 ± 0.029	0.121 ± 0.075	0.084 ± 0.022
$\Delta {\varepsilon}_{ 1}^{\infty}$ ((CH_3_)_4_NCl)	0.048 ± 0.009	0.034 ± 0.016	0.033 ± 0.012	0.055 ± 0.006	0.051 ± 0.016	0.015 ± 0.022	0.039 ± 0.015
$\Delta {\varepsilon}_{ 1}^{ 0}$ ((CH_3_)_4_NCl)	0.269 ± 0.030	0.275 ± 0.051	0.262 ± 0.032	0.276 ± 0.019	0.277 ± 0.044	0.247 ± 0.055	0.268 ± 0.012
$\Delta {\varepsilon}_{ 2}^{\infty}$ ((CH_3_)_4_NCl)	0.110 ± 0.008	0.073 ± 0.007	0.092 ± 0.015	0.091 ± 0.006	0.119 ± 0.024	0.146 ± 0.018	0.105 ± 0.026
$\Delta {\varepsilon}_{ 2}^{ 0}$ ((CH_3_)_4_NCl)	0.079 ± 0.020	0.082 ± 0.033	0.060 ± 0.057	0.066 ± 0.034	0.102 ± 0.055	0.091 ± 0.063	0.080 ± 0.016
$\Delta {{H}}_{ 1}^{ 0}$	−44.33 ± 0.03^b^	−44.20 ± 0.03^b^	−44.46 ± 0.09^b^	−45.25 ± 0.09^b^	−42.78 ± 0.05^b^	−44.06 ± 0.15^b^	−44.2 ± 0.8^b^
$\Delta {{H}}_{ 2}^{ 0}$	−3.99 ± 0.01	−2.63 ± 0.02	−0.33 ± 0.12	−1.77 ± 0.16	−4.47 ± 0.20	−1.64 ± 0.29	−2.5 ± 1.6
$\Delta {\varepsilon}_{ 1}^{\prime}$	−0.96 ± 0.03	−2.35 ± 0.70	−1.73 ± 0.34	−2.76 ± 0.05	8.8 ± 0.6	−1.32 ± 0.12	−0.1 ± 4.4
$\Delta {\varepsilon}_{ 2}^{\prime}$	−0.85 ± 0.03	−1.75 ± 0.38	1.82 ± 3.00	−0.82 ± 0.05	7.3 ± 1.6	−2.16 ± 0.12	0.6 ± 3.6
$\Delta {\text{C}}_{\text{p1}}$	41 ± 2	41 ± 2	39 ± 2	39 ± 3	41 ± 2	38 ± 3	40 ± 1
$\Delta {\text{C}}_{\text{p2}}$	136 ± 1	108 ± 5	150 ± 4	131 ± 7	141 ± 7	138 ± 5	134 ± 14
$\Delta {\varepsilon}_{ 1}^{\prime}$ ((CH_3_)_4_NCl)				−0.09 ± 0.06			
$\Delta {\varepsilon}_{ 2}^{\prime}$ ((CH_3_)_4_NCl)				−1.78 ± 0.15			

^a^ ± 95 % CI

^b^ in kJ mol^−1^

^c^ average among glycine, alanine, valine and leucine

The protonation constants at infinite dilution are very similar among **Gly**, **Leu**, **Val** and **Ala**, while those of **Phe** and **Ser** are lower and similar between them, due to the presence of withdrawing electron groups linked to the α-carbon atom, a benzyl group in the case of **Phe** and an hydroxy-methyl group in the case of **Ser**.

The ionic strength dependence parameters, $\Delta {\varepsilon}_{\text{i}}$, are very similar for all the molecules. In addition, the values in NaCl are generally higher than those in (CH_3_)_4_NCl, according to the trend of the protonation constants in these media. This results in the formation of a more stable complex between the cation (CH_3_)_4_N^+^ and the deprotonated amino group, compared to the quite negligible interaction between Na^+^ and the carboxylate group. This fact is hardly explicable if only the electrostatic nature for this interaction is considered, since the former interaction occurs between a positively charged group, (CH_3_)_4_N^+^ and a neutral one, while the latter between Na^+^ and the negatively charged carboxylate. Therefore, it is possible that the driving force of the reaction is not completely electrostatic, possibly being an hydrophobic interaction (Bretti et al. 2015).

The values of $\Delta {{H}}_{\rm i}^{0}$ are very similar for all the amino acids considered, indicating a similar thermodynamic behavior. The first protonation step, relative to the amino group, is exothermic (average value is $\Delta {{H}}_{1}^{0}$ = −44.5 ± 0.4 kJ mol^−1^) while the second, relative to the carboxylate, is slightly negative (average value is $\Delta {{H}}_{ 2}^{ 0}$ = −2.5 ± 1.6 kJ mol^−1^).

Similar considerations can be done for the values of $\Delta {{C}}_{\text{p i}}$ among the four amino acids, as far as not determinable with great accuracy. In NaCl, the values of $\Delta {\varepsilon}_{\text{i}}^{\prime}$ generally assume negative values, except for the **Ser** and for the $\Delta {\varepsilon}_{ 2}^{\prime}$ of **Val**, for which no $\Delta {{H}}_{\text{i}}^{ 0}$ data are available at *I* = 0.5 mol dm^−3^.

Calculated values of thermodynamic parameters for the six amino acids in different conditions are tabulated as Additional file 1: Tables S25–S30).

The total solubility of **Leu** and **Phe** was determined at *T* = 298.15 K in NaCl and in (CH_3_)_4_NCl (only for **Phe**) at different salt concentrations. The experimental results are reported as Additional file 1: Tables S31 and S32. The dependence of the total and specific solubility with background salt concentration was performed as described in Eqs. (13–15) and the results are reported in Table 2. The solubility of the neutral species is very similar to the total solubility, contrary to what was found for amino acids with polar residues (Bretti et al. 2015). As expected, the solubility of **Phe**, which contains a very hydrophobic aromatic ring, is significantly lower than that of **Leu**, which also contains a hydrophobic group such as the isopropyl, in turn much lower than amino acids such as **Gly** or **Ala**. The values of the Setschenow coefficients are slightly positive, both in NaCl that in (CH_3_)_4_NCl, showing a “salting out” effect. The values of the activity coefficients of the neutral species may be determined by Eq. (13) using the Setschenow coefficients, whereas those of the charged species by means of Eq. (4). In this last case, the specific interaction coefficient, ε, are needed. Looking at Eqs. (6, 6a) it is easy to understand that these values can be calculated only if the Setschenow coefficient is known, therefore only in the case of **Phe** and **Leu**, that, according to the SIT theory (Brönsted 1922; Ciavatta 1980; Grenthe and Puigdomenech 1997; Guggenheim and Turgeon 1955; Scatchard 1936), are:

$$ \begin{aligned} & {\varepsilon \left({\hbox{Na}^{+},{\textbf{Phe}}^{-}} \right) \pm 0.01 = 0.115 + \left({0.240{-}0.115} \right)/\left({I + 1} \right)} \hfill \\&  {\varepsilon \left({\left({\hbox{CH}_{3}} \right)_{4} \hbox{N}^{+},{\textbf{Phe}}^{-}} \right) \pm 0.01 = - 0.035 + \left({0.093 + 0.035} \right)/\left({I + 1} \right)} \hfill \\ & {\varepsilon \left({\hbox{H}_{2} {\textbf{Phe}}^{+},\hbox{Cl}^{-}} \right) \pm 0.01 = 0.083 + \left({- 0.176{-}0.083} \right)/\left({I + 1} \right)} \hfill \\ &{\varepsilon \left({Na^{+},{\textbf{Leu}}^{-}} \right) \pm 0.01 = 0.195 + \left({0.410{-}0.195} \right)/\left({I + 1} \right)} \hfill \\ & {\varepsilon \left({\hbox{H}_{2} {\textbf{Leu}}^{+},\hbox{Cl}^{-}} \right) \pm 0.01 = 0.049 + \left({0.061{-}0.049} \right)/\left({I + 1} \right)} \hfill \\ \end{aligned} $$

It is important to underline that the value of the *ε* (H_2_**Phe**^+^, Cl^−^) coefficient is equal in both NaCl and (CH_3_)_4_NCl media, owing to the same anion, chloride, in the two media.

**Table 2:** Parameters for the dependence of the solubility of **Leu**, and **Phe** in NaCl and (CH_3_)_4_NCl salt concentration, according to Eqs. (13–15) at *T* = 298.15 K

Parameter	Leu	Phe
log ${\text{S}}_{ 0}^{\text{T}}$ ≈ log ${\text{S}}_{ 0}^{ 0}$	−0.772 ± 0.002^a^	−1.084 ± 0.011^a^
$a_{c}$ ≈ $k_{c}$ (NaCl)	0.105 ± 0.002	0.067 ± 0.007
−$a_{m}$ ≈ $k_{m}$ (NaCl)	0.088 ± 0.001	0.055 ± 0.006
−$a_{c}$ ≈ $k_{c}$ ((CH_3_)_4_NCl)		0.122 ± 0.007
−$a_{m}$ ≈ $k_{m}$ ((CH_3_)_4_NCl)		0.048 ± 0.005

^a^ ± 95 % CI

**Fig. 7:**
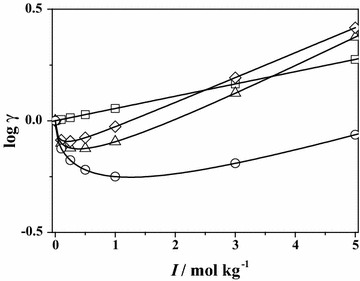
Activity coefficients of the species involved in the system H^+^/Phe in NaCl versus ionic strength at *T* = 298.15 K. *Square* neutral HL_(aq)_, *circle* H_2_L^+^, *triangle* H^+^, *diamond* L^−^

The dependence of the activity coefficients of **Phe** media on ionic strength (in NaCl) is given in Fig. 7 for all the species involved in the protonation equilibria.

Surprisingly, the solubility of **Phe** is higher than that of tyrosine (Bretti et al. 2012).

Averaging the *k*_m_ values reported in Table 2, in NaCl *k*_m_ ~ 0.07. This value may be used, in first approximation, for the solubility of other α-amino acids. If the activity coefficients of the neutral species were available for all molecules, Pitzer equation would have been useful for the modeling of the protonation constants in a wide ionic strength range and in different ionic media.

**Fig. 8:**
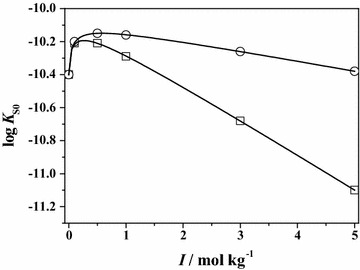
Dependence of the log *K*_S0_ values on ionic strength for **Phe** in NaCl (*square*) and (CH_3_)_4_NCl (*circle*) at *T* = 298.15 K

Using Eq. (16), the values of the solubility product of **Leu** and **Phe** can be determined in NaCl and in (CH_3_)_4_NCl at different ionic strengths and at *T* = 298.15 K. The trend of these values, reported in Table 3, is given in Fig. 8, where the log *K*_S0_ values of **Phe** are plotted against ionic strength. It can be noted that the trends are quite different, tracing that of the protonation constants.

**Table 3:** Values of the solubility product (Eq. (16)) of **Leu** and **Phe** in NaCl and in (CH_3_)_4_NCl at different ionic strengths and at *T* = 298.15 K

I/mol kg^−1^	log *K*_S0_ ± 0.01		
	**Leu**	**Phe**	**Phe**
	NaCl	NaCl	(CH_3_)_4_NCl
0	−10.55	−10.40	−10.40
0.1	−10.38	−10.21	−10.20
0.5	−10.41	−10.21	−10.15
1.0	−10.55	−10.29	−10.16
3.0	−11.18	−10.68	−10.26
5.0	−11.85	−11.10	−10.38

As mentioned earlier, the variation of the protonation constant values with the ionic strength suggests the formation of a weak complex between the amino group and the (CH_3_)_4_N^+^. This interaction has been quantified with the Δp*K* method (see the section dedicated to the determination of the weak complexes), by means of the ES2WC computer program (De Robertis et al. 1987), using the data of the six amino acids simultaneously and choosing NaCl as the baseline electrolyte. This allowed us to obtain an average value of log *K* = −0.38 ± 0.07 at infinite dilution. In the past, weak species between sodium and tetraethylammonium cations and glycinate (both with log *K*^M^ ~ −0.35 at infinite dilution and *T* = 298.15 K) were reported (De Stefano et al. 1995).

### Literature comparison

As regards the protonation constants of the studied amino acids, a critical comparison was done in previous sections, representing the core of the papers. Therefore, this section is dedicated to the comparison of other quantities, such as solubility and enthalpy changes, or comparisons among different amino acids, to evaluate the effect of various substituents on the acid–base properties of this class of molecules.

In the past, the total solubility of **Leu** was determined by Amend (Amend and Helgeson 1997) (log ${\text{S}}_{ 0}^{\text{T}}$ = −0.777), Dalton (Dalton and Schmidt 1933) (log ${\text{S}}_{ 0}^{\text{T}}$ = −0.733) and Dunn (Dunn et al. 1933) (log ${\text{S}}_{ 0}^{\text{T}}$ = −1.046) and the first value is not significantly different than our experimental value, log ${\text{S}}_{ 0}^{\text{T}}$ = −0.772. The same authors reported also the total solubility of **Phe** (log ${\text{S}}_{ 0}^{\text{T}}$ = −0.770, −1.068, −1.066 same order of above). In this case, the last two values are in a good agreement with the value here reported (log ${\text{S}}_{ 0}^{\text{T}}$ = −1.084). The solubility of **Phe** is higher than that of l-tyrosine, that has a hydroxyl group in para position to the alanil group. In fact, for l-tyrosine Bretti et al. (2012) found log ${\text{S}}_{ 0}^{\text{T}}$ = −2.648 [other values are log ${\text{S}}_{ 0}^{\text{T}}$ = −2.602 (Dalton and Schmidt 1933) and −2.523 (Amend and Helgeson 1997)].

Pettit et al. (1984) reported that the protonation constant of the amino group of phenylalanine (log *K*_1_^H^ = 9.15 at *I* = 0.1 mol dm^−3^ in KNO_3_) is slightly more basic that the corresponding value for tyrosine, which has a ionizable hydroxyl group linked to the aromatic ring (log *K*_2_^H^ = 9.03 in the same conditions), in turn higher than 3,4-dihydroxyphenylalanine (two ionizable hydroxyl groups on the aromatic ring, log *K*_3_^H^ = 8.77 in the same conditions). Note that hydroxyl groups of the latter molecules are more basic than the amino group, and the indexes of the protonation constants change accordingly. An opposite trend is observed for the enthalpy change, in fact the value reported for tyrosine is higher than that of phenylalanine, being Δ*H*_2_^0^ = −42.4 (Pettit 1984) or −38.1 kJ mol^−1^ (Bretti et al. 2012) for tyrosine and Δ*H*_1_^0^ = −43.2 (Pettit 1984) or −45.2 (this work) kJ mol^−1^ for phenylalanine. These trends may be ascribable to the inductive effect produced by the presence of the hydroxyl groups.

As regards serine and threonine, differing only for the presence of a methyl group in the latter, the protonation constants of serine (log ${\text{K}}_{ 1}^{\text{H0}}$ = 9.255 at infinite dilution) are quite higher than that of threonine [log ${\text{K}}_{ 1}^{\text{H0}}$ = 9.100 in the same conditions (Martell et al. 2004)], whereas protonation enthalpy are very similar (Martell et al. 2004). On the contrary, cysteine, in which the hydroxyl group is substituted with a thiol, the amino group has a greater basicity, being log ${\text{K}}_{ 1}^{\text{H0}}$ = 10.21 at infinite dilution (Sharma et al. 2002). Protonation constants of structural isomers, such as the couple leucine and isoleucine or valine and norvaline, are not significantly different (Martell et al. 2004).

Glycine, alanine and serine protonation constants were measured at high NaCl concentration and, in NaCl and artificial sea water (De Stefano et al. 1995, 2000): many data are reported in that works at different temperatures and ionic strengths, in several supporting electrolytes. Izatt et al. (1992) studied the effect of temperature and pressure on the protonation of glycine, determining the value of Δ*C*_p1_ = 41.2 J K^−1^ mol^−1^, not significantly different than the average value obtained in this work, namely Δ*C*_p1_ = 40 ± 1 J K^−1^ mol^−1^.

## Conclusions

This work contributes to the rationalization of the knowledge of the acid–base and thermodynamic properties of six natural occurring amino acids. In particular, the protonation constants and enthalpy change values are reported at different ionic strengths and temperatures in two ionic media, namely NaCl and (CH_3_)_4_NCl. These two solutes have different effects on molecular behavior of the water, producing diverse trends in properties measured in these media. For example, at *I* > 0.5 mol kg^−1^, the first protonation constant of serine (and other similar amino acids) increases with increasing ionic strength in (CH_3_)_4_NCl, and remains quite constant in NaCl. This can be due to the weak interactions between cations and the deprotonated ligands. The dependence on ionic strength of the protonation constants was studied using both the Debye-Hückel type and the SIT equations and the differences between the protonation constants determined in the two ionic media were also interpreted in terms of formation of weak complexes. No data, to our knowledge, are reported in the literature for log $K_{\rm i}^{\rm H}$ and $\Delta H_{\rm i}$ relative to the proton binding reactions obtained in aqueous solutions of tetramethylammonium salts. The ionic strength dependence parameters of protonation constants resulted quite similar among all the amino acids here investigated and, for those having non-polar side chain, also log $K_{\rm i}^{\rm H0}$ is fairly constant, being log $K_{1}^{\rm H0} = 9.80 \pm 0.08$ and log $K_{2}^{\rm H0} = 2.32 \pm 0.03$ as average values. This also applies to $\Delta H_{\rm i}^{0}$ values: $\Delta H_{1}^{0} = \left( { - 44.5 \pm 0.4} \right)$ kJ mol^−1^, and $\Delta H_{2}^{0} = \left( { - 2.5 \pm 1.6} \right)$ kJ mol^−1^ and to Δ*C*_pi_: Δ*C*_p1_ = (40 ± 1) J K^−1^ mol^−1^ and Δ*C*_p2_ = (134 ± 14) J K^−1^ mol^−1^. These results are particularly important for three reasons: (1) using these few number of parameters and the proper equations, it is possible to calculate the protonation constants of each amino acid in a wide range of experimental conditions; (2) the need of reliable thermodynamic data in different condition is particularly important for applications to real matrices, and (3) similarities in the behaviour of the six amino acids allows one to built models for the thermodynamic properties of this class of ligands. In addition, the errors associated to all parameters are quite low. For this reason, in Table 4 some recommended (flagged as R), tentative (T) or provisional (P) log $K_{\rm i}^{\rm H}$ and $\Delta H_{\rm i}^{0}$ values are proposed in NaCl. Data in (CH_3_)_4_NCl are not reported since there are not literature comparisons. The flag associated to each value depends on the amount of experimental data and on the value of the confidence interval. The total solubilities of leucine and phenylalanine at different salt concentrations [NaCl 0 to 5 mol kg^−1^; (CH_3_)_4_NCl 0 to 3.5 mol kg^−1^] are reported for the first time. From the dependence on salt concentration it was possible to calculate the Setschenow coefficients and therefore activity coefficients of neutral species. Regarding glycine and alanine, in the literature it was reported a quite high solubility (Carta 1998, 1999; Carta and Tola 1996) that makes difficult the calculation of Setschenow coefficients because of the self association at high concentration.

**Table 4:** Recommended (R), tentative (T) or provisional (P) log $K_{\rm i}^{\rm H}$ and $\Delta H_{\rm i}$ values of glycine, alanine, valine, leucine, serine and pheylalanine in NaCl at different temperatures and ionic strength

	*I* ^a^	*T* ^b^	log ${\text{K}}_{1}^{\text{H}}$	FLAG	$\Delta H_{1}^{0}$ ^c^	FLAG	log ${\text{K}}_{2}^{\text{\rm H}}$	FLAG	$\Delta H_{2}^{0}$ ^c^	FLAG
Ala	0	298.15	9.912 ± 0.013^d^	R	−44.2 ± 0.3^d^	R	2.365 ± 0.015^d^	R	−2.6 ± 0.3^d^	R
	0.1	298.15	9.727 ± 0.010	R	−45.1 ± 0.3	R	2.374 ± 0.013	R	−2.7 ± 0.3	R
	0.5	298.15	9.712 ± 0.009	R	−46.5 ± 0.3	R	2.414 ± 0.009	R	−3.4 ± 0.3	R
	1.0	298.15	9.777 ± 0.013	R	−47.8 ± 0.5	R	2.467 ± 0.009	R	−4.3 ± 0.3	R
	3.0	298.15	10.123 ± 0.020	T	−52.7 ± 1.9	T	2.690 ± 0.014	R	−7.8 ± 1.1	T
	5.0	298.15	10.493 ± 0.023	P	−57.6 ± 3.3	P	2.916 ± 0.016	P	−11.3 ± 1.8	P
	0.15	310.15	9.402 ± 0.010	R	−45.0 ± 0.3	R	2.369 ± 0.012	R	−1.5 ± 0.3	R
Gly	0	298.15	9.777 ± 0.004	R	−44.3 ± 0.3	R	2.329 ± 0.007	R	−4.0 ± 0.3	R
	0.1	298.15	9.588 ± 0.004	R	−45.1 ± 0.3	R	2.338 ± 0.006	R	−4.1 ± 0.3	R
	0.5	298.15	9.562 ± 0.007	R	−45.8 ± 0.3	R	2.376 ± 0.007	R	−4.4 ± 0.3	R
	1.0	298.15	9.619 ± 0.010	R	−46.5 ± 0.3	R	2.424 ± 0.009	R	−4.8 ± 0.3	R
	3.0	298.15	9.946 ± 0.014	T	−48.7 ± 0.5	T	2.619 ± 0.014	T	−6.5 ± 0.3	T
	5.0	298.15	10.304 ± 0.016	P	−50.7 ± 0.5	P	2.814 ± 0.016	P	−8.2 ± 0.3	P
	0.15	310.15	9.262 ± 0.004	R	−44.9 ± 0.3	R	2.326 ± 0.006	R	−2.5 ± 0.3	R
Leu	0	298.15	9.778 ± 0.005	R	−45.3 ± 0.3	R	2.321 ± 0.008	R	−1.9 ± 0.3	R
	0.1	298.15	9.594 ± 0.004	R	−46.2 ± 0.3	R	2.329 ± 0.007	R	−1.9 ± 0.3	R
	0.5	298.15	9.589 ± 0.004	R	−47.7 ± 0.3	R	2.374 ± 0.005	R	−2.3 ± 0.3	R
	1.0	298.15	9.677 ± 0.004	R	−49.3 ± 0.3	R	2.445 ± 0.004	R	−2.7 ± 0.3	R
	3.0	298.15	10.137 ± 0.007	T	−55.0 ± 0.5	T	2.772 ± 0.006	T	−4.3 ± 0.5	T
	5.0	298.15	10.632 ± 0.008	P	−60.6 ± 0.5	P	3.117 ± 0.007	P	−5.9 ± 0.5	P
	0.15	310.15	9.261 ± 0.004	R	−46.2 ± 0.3	R	2.330 ± 0.007	R	−0.5 ± 0.3	R
Phe	0	298.15	9.258 ± 0.006	R	−44.1 ± 0.3	R	2.144 ± 0.007	R	−1.6 ± 0.3	R
	0.1	298.15	9.071 ± 0.006	R	−44.8 ± 0.3	R	2.157 ± 0.004	R	−1.9 ± 0.3	R
	0.5	298.15	9.046 ± 0.014	R	−45.7 ± 0.3	R	2.225 ± 0.014	R	−2.7 ± 0.3	R
	1.0	298.15	9.097 ± 0.022	T	−46.6 ± 0.3	R	2.325 ± 0.023	T	−3.8 ± 0.3	R
	3.0	298.15	9.381 ± 0.034	T	−49.5 ± 0.5	T	2.777 ± 0.037	P	−8.1 ± 0.5	T
	5.0	298.15	9.686 ± 0.038	P	−52.2 ± 0.5	P	3.249 ± 0.042	P	−12.5 ± 0.7	P
	0.15	310.15	8.747 ± 0.006	R	−44.7 ± 0.3	R	2.151 ± 0.005	R		
Ser	0	298.15	9.255 ± 0.009	R	−42.8 ± 0.5	T	2.162 ± 0.005	R	−4.5 ± 0.5	T
	0.1	298.15	9.068 ± 0.007	R	−42.5 ± 0.4	R	2.169 ± 0.005	R	−3.7 ± 0.5	T
	0.5	298.15	9.046 ± 0.006	R	−39.4 ± 0.5	T	2.199 ± 0.009	R	−0.8 ± 1.5	P
	1.0	298.15	9.105 ± 0.007	R			2.241 ± 0.013	T		
	3.0	298.15	9.431 ± 0.010	T			2.424 ± 0.020	T		
	5.0	298.15	9.785 ± 0.011	P			2.613 ± 0.022	P		
	0.15	310.15	8.763 ± 0.007	R	−41.8 ± 0.3	R	2.161 ± 0.005	R	−1.7 ± 0.4	R
Val	0	298.15	9.730 ± 0.008	R	−44.5 ± 0.3	R	2.286 ± 0.005	R	−0.3 ± 0.5	T
	0.1	298.15	9.549 ± 0.006	R	−45.3 ± 0.3	R	2.293 ± 0.006	R	−0.1 ± 0.6	T
	0.5	298.15	9.544 ± 0.018	R	−46.4 ± 0.3	R	2.324 ± 0.020	T		
	1.0	298.15	9.615 ± 0.028	T	−47.4 ± 0.5	T	2.365 ± 0.031	T		
	3.0	298.15	9.961 ± 0.045	P	−51.1 ± 0.5	T	2.537 ± 0.047	P		
	5.0	298.15	10.324 ± 0.050	P	−54.6 ± 1.7	T	2.712 ± 0.052	P		
	0.15	310.15	9.225 ± 0.007	R	−45.1 ± 0.3	R	2.309 ± 0.009	R	1.7 ± 0.9	P

^a^In mol kg^−1^

^ b^ in K

^ c^ in kJ mol^−1^

^d^ 95 % CI

## Additional files

10.1186/s40064-016-2568-8 Experimental and calculated values of Protonation constants, solubility and thermodynamic parameters.
10.1186/s40064-016-2568-8 Calculation sheet.

## Abbreviations

Gly: glycine
Ala: alanine
Val: valine
Leu: leucine
Ser: serine
Phe: phenylalanine
DON: dissolved organic nitrogen
DHt: Debye–Hückel type
SIT: Specific ion Interaction Theory
IUPAC: International Union of Pure and Applied Chemistry
