# Supplementary material for: Modeling solubility and acid–base properties of some amino acids in aqueous NaCl and (CH3)4NCl aqueous solutions at different ionic strengths and temperatures
Source: Springerplus. 2016 Jun 30;5(1):928. doi: 10.1186/s40064-016-2568-8 (PMC4927535; doi:10.1186/s40064-016-2568-8)
Supplement: Supplementary file 1 — 10.1186/s40064-016-2568-8 Experimental and calculated values of Protonation constants, solubility and thermodynamic parameters. [file 40064_2016_2568_MOESM1_ESM.docx]

Supplementary material of the paper

Modeling solubility and acid-base properties of some simple amino acids in aqueous NaCl and (CH_3_)_4_NCl aqueous solutions at different ionic strengths and temperatures

Submitted on March 31^st^, 2016 to Amino Acids

By: *Clemente Bretti, Ottavia Giuffrè, Gabriele Lando, and Silvio Sammartano**

Dipartimento di Scienze Chimiche, Biologiche, Farmaceutiche ed Ambientali, Università di Messina, Viale F. Stagno d’Alcontres 31, 98166 Messina, Italy

* Correspondence to: Silvio Sammartano, E-mail: ssammartano@unime.it; Tel: +39-090-6765749; Fax: +39-090-392827

**Table 1S** Literature values for the protonation of L-Leucine at different temperatures, ionic strengths and in different ionic media

| Ionic medium | *T* / K | *I* / mol dm^-3^ | log $\text{K}_{\text{1}}^{\text{H}}$ | log $\text{K}_{\text{2}}^{\text{H}}$ | Ref. |
| --- | --- | --- | --- | --- | --- |
|  | 274.15 | 0 | 10.454 | 2.383 | (Sóvágo et al., 1993), (Christensen et al., 1976) |
|  | 285.65 | 0 | 10.095 | 2.348 | (Sóvágo et al., 1993), (Christensen et al., 1976) |
|  | 298.15 | 0 | 9.744 | 2.328 | (Sóvágo et al., 1993), (Christensen et al., 1976) |
|  | 310.65 | 0 | 9.434 | 2.327 | (Sóvágo et al., 1993), (Christensen et al., 1976) |
|  | 323.15 | 0 | 9.142 | 2.333 | (Sóvágo et al., 1993), (Christensen et al., 1976) |
| ? | 298.15 | 0.100 | 9.68 | ⎯ | (Sóvágo et al., 1993) |
| NaCl | 293.15 | 0.150 | 9.73 | ⎯ | (Pettit and Powell, 2001), (Sóvágo et al., 1993) |
|  | 298.15 | 0 | 9.78 | 2.34 | (Pettit and Powell, 2001), (Vilarino et al., 1997) |
|  | 298.15 | 0 | 9.93 | 2.58 | (Pettit and Powell, 2001) |
|  | 298.15 | 0 | 9.50 | 2.44 | (Pettit and Powell, 2001) |
|  | 298.15 | 0 | 9.59 | 2.44 | (Pettit and Powell, 2001) |
|  | 293.15 | 0 | 9.92 | ⎯ | (Pettit and Powell, 2001) |
|  | 298.15 | 0.010 | 9.60 | 2.36 | (Sóvágo et al., 1993) |
|  | 298.15 | 0.010 | 9.77 | 2.36 | (Sóvágo et al., 1993) |
|  | 293.15 | <0.010 | 9.92 | ⎯ | (Sóvágo et al., 1993) |
|  | 295.15 | <0.010 | 9.62 | 2.37 | (Pettit and Powell, 2001), (Neveu et al., 1976) |
| Unknown | 298.15 | 0.100 | 9.71 | 2.39 | (Sóvágo et al., 1993) |
| Unknown | 298.15 | 0.100 | 9.69 | 2.36 | (Sóvágo et al., 1993) |
| Unknown | 298.15 | 0.100 | 9.76 | 2.33 | (Sóvágo et al., 1993) |
| KCl | 298.15 | 0.100 | 9.66 | 2.32 | (Pettit and Powell, 2001) |
| KCl | 298.15 | 0.100 | 9.58 | 2.34 | (Pettit and Powell, 2001), (Sóvágo et al., 1993) |
| KCl | 308.15 | 0.100 | 9.09 | 1.85 | (Pettit and Powell, 2001), (Sóvágo et al., 1993) |
| KCl | 318.15 | 0.100 | 8.38 | 1.14 | (Pettit and Powell, 2001), (Sóvágo et al., 1993) |
| KCl | 298.15 | 0.200 | 9.77 | 2.45 | (Pettit and Powell, 2001), (Sóvágo et al., 1993) |
| KCl | 293.15 | 1.000 | 9.62 | ⎯ | (Pettit and Powell, 2001), (Sóvágo et al., 1993) |
| NaClO_4_ | 298.15 | 0.100 | 9.66 | 2.32 | (Sóvágo et al., 1993) |
| NaClO_4_ | 310.15 | 0.150 | 9.266 | 2.324 | (Pettit and Powell, 2001), (Sóvágo et al., 1993) |
| NaClO_4_ | 298.15 | 0.200 | 9.67 | 2.33 | (Pettit and Powell, 2001) |
| NaClO_4_ | 298.15 | 0.200 | 9.71 | 2.32 | (Sóvágo et al., 1993) |
| NaClO_4_ | 293.15 | 1.000 | 9.62 | 2.37 | (Pettit and Powell, 2001), (Sóvágo et al., 1993) |
| NaNO_3_ | 298.15 | 0.100 | 9.63 | 2.69 | (Pettit and Powell, 2001), ([Zhang et al., 2000a](#_ENREF_68)) |
| NaNO_3_ | 298.15 | 0.100 | 9.42 | 2.28 | (Pettit and Powell, 2001), ([Zhang et al., 2000b](#_ENREF_69)) |
| KNO_3_ | 298.15 | 0.100 | 9.67 | 2.46 | (Sóvágo et al., 1993) |
| KNO_3_ | 298.15 | 0.100 | 9.703 | 2.286 | (Pettit and Powell, 2001), (Sóvágo et al., 1993) |
| KNO_3_ | 298.15 | 0.100 | 9.621 | 2.645 | (Pettit and Powell, 2001), (Sóvágo et al., 1993), (Nourmand and Meissami, 2001) |
| KNO_3_ | 298.15 | 0.100 | 9.51 | 2.35 | (Pettit and Powell, 2001), (Sóvágo et al., 1993) |
| KNO_3_ | 298.15 | 0.100 | 9.558 | 2.345 | (Pettit and Powell, 2001), (Vilarino et al., 1997) |
| KNO_3_ | 298.15 | 0.100 | 9.71 | 2.28 | (Pettit and Powell, 2001) |
| KNO_3_ | 298.15 | 0.100 | 9.25 | 2.74 | (Pettit and Powell, 2001) |
| KNO_3_ | 298.15 | 0.100 | 9.89 | 2.23 | (Pettit and Powell, 2001) |
| KNO_3_ | 298.15 | 0.100 | 9.75 | ⎯ | (Pettit and Powell, 2001) |
| KNO_3_ | 310.15 | 0.150 | 9.36 | 2.36 | (Sóvágo et al., 1993) |
| KNO_3_ | 298.15 | 0.160 | 9.54 | ⎯ | (Sóvágo et al., 1993) |
| KNO_3_ | 308.15 | 0.200 | 9.23 | 2.27 | (Pettit and Powell, 2001), (Sóvágo et al., 1993) |
| KNO_3_ | 298.15 | 0.300 | 9.55 | 2.37 | (Vilarino et al., 1997) |
| KNO_3_ | 298.15 | 0.500 | 9.56 | 2.42 | (Vilarino et al., 1997) |
| KNO_3_ | 298.15 | 0.500 | 9.58 | 2.42 | (Pettit and Powell, 2001), (Sóvágo et al., 1993) |
| KNO_3_ | 298.15 | 0.500 | 9.75 | 2.42 | (Pettit and Powell, 2001), (Sóvágo et al., 1993) |
| KNO_3_ | 293.15 | 0.500 | 9.83 | 2.73 | (Pettit and Powell, 2001) |
| KNO_3_ | 298.15 | 0.700 | 9.58 | 2.44 | (Vilarino et al., 1997) |
| KNO_3_ | 298.15 | 0.900 | 9.63 | 2.49 | (Vilarino et al., 1997) |
| KNO_3_ | 298.15 | 1.000 | 9.74 | 2.32 | (Pettit and Powell, 2001) |
| KNO_3_ | 298.15 | 1.100 | 9.66 | 2.49 | (Vilarino et al., 1997) |
| KNO_3_ | 298.15 | 1.300 | 9.68 | 2.51 | (Vilarino et al., 1997) |
| Ionic medium | *T* / K | *I* / mol dm^-3^ | Δ$\text{H}_{\text{1}}^{\text{0}}$ / kJ mol^-1^ | Δ$\text{H}_{\text{2}}^{\text{0}}$ / kJ mol^-1^ | Ref. |
|  | 283.15 | 0 | -46 | -3 | (Sóvágo et al., 1993), (Christensen et al., 1976) |
|  | 298.15 | 0 | -45.39 | -1.6 | (Sóvágo et al., 1993), (Christensen et al., 1976) |
|  | 313.15 | 0 | -44.68 | 0.8 | (Sóvágo et al., 1993), (Christensen et al., 1976) |
|  | 274.15 | 0 | -45.48 | -4.937 | (Sóvágo et al., 1993), (Christensen et al., 1976) |
|  | 285.65 | 0 | -45.773 | -3.598 | (Sóvágo et al., 1993), (Christensen et al., 1976) |
|  | 298.15 | 0 | -45.606 | -1.757 | (Sóvágo et al., 1993), (Christensen et al., 1976) |
|  | 310.65 | 0 | -44.894 | 0.377 | (Sóvágo et al., 1993), (Christensen et al., 1976) |
|  | 323.15 | 0 | -43.555 | 2.929 | (Sóvágo et al., 1993), (Christensen et al., 1976) |
|  | 293.15 | 0.010 | -45.2 | ⎯ | (Sóvágo et al., 1993), (Christensen et al., 1976) |
|  | 298.15 | 0 | ⎯ | -1.51 | (Pettit and Powell, 2001) |
| KNO_3_ | 298.15 | 0 | -47.4 | -2.9 | (Pettit and Powell, 2001) |
| KCl | 298.15 | 0.160 | -44.9 | ⎯ | (Sóvágo et al., 1993), (Christensen et al., 1976) |
|  | 298.15 | 0.100 | -44.9 | ⎯ | (Pettit and Powell, 2001) |

**Table 2S** Literature values for the protonation of L-Valine at different temperatures, ionic strengths and in different ionic media

| Ionic medium | *T* / K | *I* / mol dm^-3^ | log $\text{K}_{\text{1}}^{\text{H}}$ | log $\text{K}_{\text{2}}^{\text{H}}$ | Ref. |
| --- | --- | --- | --- | --- | --- |
|  | 298.15 | 0.1-0.2 | 9.54 | 2.28 | Recommended values([Sóvágó et al., 1993](#_ENREF_63)) |
|  | 298.15 | 0.05-0.2 | 9.60 | 2.38 | Tentative values ([Sóvágó et al., 1993](#_ENREF_63)) |
|  | 283.15 | 0 | 10.133 | 2.3 | ([Martell et al., 2004](#_ENREF_41)) |
|  | 298.15 | 0 | 9.719 | 2.286 | ([Martell et al., 2004](#_ENREF_41)) |
|  | 313.15 | 0 | 9.345 | 2.294 | ([Martell et al., 2004](#_ENREF_41)) |
|  | 274.15 | 0 | 10.413 | 2.320 | ([Sóvágó et al., 1993](#_ENREF_63)), ([Christensen et al., 1976](#_ENREF_15)) |
|  | 285.65 | 0 | 10.064 | 2.297 | ([Sóvágó et al., 1993](#_ENREF_63)), ([Christensen et al., 1976](#_ENREF_15)) |
|  | 298.15 | 0 | 9.719 | 2.286 | ([Sóvágó et al., 1993](#_ENREF_63)), ([Christensen et al., 1976](#_ENREF_15)) |
|  | 310.65 | 0 | 9.405 | 2.292 | ([Sóvágó et al., 1993](#_ENREF_63)), ([Christensen et al., 1976](#_ENREF_15)) |
|  | 323.15 | 0 | 9.124 | 2.31 | ([Sóvágó et al., 1993](#_ENREF_63)), |
|  | 283.15 | 0 | 10.04 | 2.33 | ([Sóvágó et al., 1993](#_ENREF_63)) |
|  | 288.15 | 0 | 9.9 | 2.33 | ([Sóvágó et al., 1993](#_ENREF_63)) |
|  | 298.15 | 0 | 9.62 | 2.32 | ([Sóvágó et al., 1993](#_ENREF_63)), ([Pettit and Powell, 2001](#_ENREF_51)) |
|  | 303.15 | 0 | 9.49 | 2.32 | ([Sóvágó et al., 1993](#_ENREF_63)) |
|  | 308.15 | 0 | 9.38 | 2.32 | ([Sóvágó et al., 1993](#_ENREF_63)) |
|  | 313.15 | 0 | 9.25 | 2.32 | ([Sóvágó et al., 1993](#_ENREF_63)) |
|  | 293.15 | 0 | 9.59 | ⎯ | ([Sóvágó et al., 1993](#_ENREF_63)), ([Pettit and Powell, 2001](#_ENREF_51)) |
|  | 298.15 | 0 | 9.72 | ⎯ | ([Pettit and Powell, 2001](#_ENREF_51)) |
|  | 298.15 | 0.010 | 9.62 | 2.32 | ([Sóvágó et al., 1993](#_ENREF_63)), ([Pettit and Powell, 2001](#_ENREF_51)) |
|  | 298.15 | 0.010 | 9.72 | 2.20 | ([Sóvágó et al., 1993](#_ENREF_63)), ([Pettit and Powell, 2001](#_ENREF_51)) |
|  | 298.15 | 0.020 | 9.81 | 2.29 | ([Sóvágó et al., 1993](#_ENREF_63)), ([Pettit and Powell, 2001](#_ENREF_51)) |
| Unknown | 298.15 | 0.100 | 9.527 | 2.243 | ([Pettit and Powell, 2001](#_ENREF_51)) |
| Unknown | 298.15 | 0.150 | 9.61 | ⎯ | ([Sóvágó et al., 1993](#_ENREF_63)), ([Pettit and Powell, 2001](#_ENREF_51)) |
| Unknown | 303.15 | 0.150 | 9.46 | ⎯ | ([Sóvágó et al., 1993](#_ENREF_63)) |
| Unknown | 313.15 | 0.150 | 9.15 | ⎯ | ([Sóvágó et al., 1993](#_ENREF_63)) |
| Unknown | 310.15 | 0.150 | 9.17 | 2.27 | ([Martell et al., 2004](#_ENREF_41)) |
| Unknown | 298.15 | 0.500 | 9.45 | 2.3 | ([Martell et al., 2004](#_ENREF_41)) |
| Unknown | 298.15 | 1.000 | 9.5 | 2.33 | ([Martell et al., 2004](#_ENREF_41)) |
| KCl | 298.15 | 0.100 | 9.71 | 2.48 | ([Sóvágó et al., 1993](#_ENREF_63)) |
| KCl | 293.15 | 0.100 | 9.65 | 2.24 | ([Sóvágó et al., 1993](#_ENREF_63)), ([Pettit and Powell, 2001](#_ENREF_51)) |
| KCl | 298.15 | 0.100 | 9.54 | 2.28 | ([Pettit and Powell, 2001](#_ENREF_51)) |
| KCl | 298.15 | 0.100 | 9.548 | 2.302 | ([Pettit and Powell, 2001](#_ENREF_51)) |
| KCl | 293.15 | 1.000 | 9.50 | ⎯ | ([Sóvágó et al., 1993](#_ENREF_63)), ([Pettit and Powell, 2001](#_ENREF_51)) |
| NaNO_3_ | 298.15 | 0.100 | 9.62 | 2.57 | ([Pettit and Powell, 2001](#_ENREF_51)) |
| NaNO_3_ | 298.15 | 0.100 | 9.46 | 2.30 | ([Pettit and Powell, 2001](#_ENREF_51)) |
| NaNO_3_ | 298.15 | 0.100 | 9.64 | 2.21 | ([Pettit and Powell, 2001](#_ENREF_51)) |
| NaNO_3_ | 298.15 | 1.000 | 9.565 | 2.316 | ([Sóvágó et al., 1993](#_ENREF_63)) |
| KNO_3_ | 295.15 | 0.100 | 9.59 | 2.20 | ([Sóvágó et al., 1993](#_ENREF_63)), ([Pettit and Powell, 2001](#_ENREF_51)) |
| KNO_3_ | 298.15 | 0.100 | 9.63 | ⎯ | ([Pettit and Powell, 2001](#_ENREF_51)) |
| KNO_3_ | 298.15 | 0.100 | 9.51 | 2.26 | ([Pettit and Powell, 2001](#_ENREF_51)) |
| KNO_3_ | 298.15 | 0.100 | 9.603 | 2.693 | ([Sóvágó et al., 1993](#_ENREF_63)) |
| KNO_3_ | 298.15 | 0.100 | 9.52 | 2.36 | ([Sóvágó et al., 1993](#_ENREF_63)) |
| KNO_3_ | 298.15 | 0.100 | 9.61 | ⎯ | ([Sóvágó et al., 1993](#_ENREF_63)) |
| KNO_3_ | 298.15 | 0.100 | 9.63 | 2.39 | ([Sóvágó et al., 1993](#_ENREF_63)) |
| KNO_3_ | 298.15 | 0.100 | 9.50 | 2.28 | ([Sóvágó et al., 1993](#_ENREF_63)), ([Pettit and Powell, 2001](#_ENREF_51)) |
| KNO_3_ | 298.15 | 0.100 | 9.573 | ⎯ | ([Sóvágó et al., 1993](#_ENREF_63)), ([Pettit and Powell, 2001](#_ENREF_51)) |
| KNO_3_ | 298.15 | 0.100 | 9.74 | 2.32 | ([Pettit and Powell, 2001](#_ENREF_51)) |
| KNO_3_ | 298.15 | 0.100 | 9.62 | 2.38 | ([Pettit and Powell, 2001](#_ENREF_51)) |
| KNO_3_ | 298.15 | 0.100 | 9.60 | 2.64 | ([Pettit and Powell, 2001](#_ENREF_51)) |
| KNO_3_ | 298.15 | 0.100 | 9.58 | ⎯ | ([Pettit and Powell, 2001](#_ENREF_51)) |
| KNO_3_ | 298.15 | 0.100 | 9.51 | 2.26 | ([Sóvágó et al., 1993](#_ENREF_63)) |
| KNO_3_ | 308.15 | 0.100 | 9.22 | ⎯ | ([Pettit and Powell, 2001](#_ENREF_51)) |
| KNO_3_ | 298.15 | 0.110 | 9.47 | 2.25 | ([Sóvágó et al., 1993](#_ENREF_63)) |
| KNO_3_ | 310.15 | 0.150 | 9.316 | ⎯ | ([Sóvágó et al., 1993](#_ENREF_63)) |
| KNO_3_ | 310.15 | 0.150 | 9.32 | 2.34 | ([Sóvágó et al., 1993](#_ENREF_63)), ([Pettit and Powell, 2001](#_ENREF_51)) |
| KNO_3_ | 310.15 | 0.150 | 9.31 | 2.24 | ([Pettit and Powell, 2001](#_ENREF_51)) |
| KNO_3_ | 298.15 | 0.160 | 9.44 | ⎯ | ([Sóvágó et al., 1993](#_ENREF_63)), ([Christensen et al., 1976](#_ENREF_15)) |
| KNO_3_ | 308.15 | 0.200 | 9.27 | 2.25 | ([Sóvágó et al., 1993](#_ENREF_63)), ([Pettit and Powell, 2001](#_ENREF_51)) |
| KNO_3_ | 293.15 | 0.500 | 9.80 | 2.74 | ([Pettit and Powell, 2001](#_ENREF_51)) |
| KNO_3_ | 303.15 | 0.500 | 9.48 | ⎯ | ([Sóvágó et al., 1993](#_ENREF_63)) |
| KNO_3_ | 296.15 | 0.500 | 9.62 | 2.35 | ([Sóvágó et al., 1993](#_ENREF_63)) |
| KNO_3_ | 298.15 | 0.500 | 9.54 | ⎯ | ([Sóvágó et al., 1993](#_ENREF_63)), ([Pettit and Powell, 2001](#_ENREF_51)) |
| KNO_3_ | 298.15 | 0.500 | 9.51 | 2.31 | ([Sóvágó et al., 1993](#_ENREF_63)) |
| KNO_3_ | 298.15 | 1.000 | 9.71 | 2.28 | ([Pettit and Powell, 2001](#_ENREF_51)) |
| KNO_3_ | 303.15 | 1.000 | 9.44 | ⎯ | ([Sóvágó et al., 1993](#_ENREF_63)), ([Pettit and Powell, 2001](#_ENREF_51)) |
| KNO_3_ | 303.15 | 1.000 | 9.46 | ⎯ | ([Pettit and Powell, 2001](#_ENREF_51)) |
| NaClO_4_ | 298.15 | 0.100 | 9.57 | 2.34 | ([Pettit and Powell, 2001](#_ENREF_51)) |
| NaClO_4_ | 303.15 | 0.100 | 9.49 | 2.41 | ([Sóvágó et al., 1993](#_ENREF_63)) |
| NaClO_4_ | 310.15 | 0.150 | 9.262 | 2.388 | ([Pettit and Powell, 2001](#_ENREF_51)) |
| NaClO_4_ | 310.15 | 0.150 | 9.263 | 2.392 | ([Sóvágó et al., 1993](#_ENREF_63)), ([Pettit and Powell, 2001](#_ENREF_51)) |
| NaClO_4_ | 310.15 | 0.150 | 9.25 | 2.30 | ([Pettit and Powell, 2001](#_ENREF_51)) |
| NaClO_4_ | 298.15 | 0.200 | 9.60 | 2.32 | ([Sóvágó et al., 1993](#_ENREF_63)), ([Pettit and Powell, 2001](#_ENREF_51)) |
| NaClO_4_ | 298.15 | 0.500 | 9.62 | 2.35 | ([Sóvágó et al., 1993](#_ENREF_63)), ([Pettit and Powell, 2001](#_ENREF_51)) |
| NaClO_4_ | 293.15 | 1.000 | 9.59 | 2.38 | ([Sóvágó et al., 1993](#_ENREF_63)), ([Pettit and Powell, 2001](#_ENREF_51)) |
| NaClO_4_ | 298.15 | 1.000 | 9.68 | ⎯ | ([Sóvágó et al., 1993](#_ENREF_63)) |
| NaClO_4_ | 298.15 | 3.000 | 9.75 | ⎯ | ([Pettit and Powell, 2001](#_ENREF_51)) |
|  |  |  |  |  |  |
| Ionic medium | *T* / K | *I* / mol dm^-3^ | Δ$\text{H}_{\text{1}}^{\text{0}}$ / kJ mol^-1^ | Δ$\text{H}_{\text{2}}^{\text{0}}$ / kJ mol^-1^ | Ref. |
|  | 274.15 | 0 | -43.388 | -3.724 | ([Sóvágó et al., 1993](#_ENREF_63)), ([Christensen et al., 1976](#_ENREF_15)) |
|  | 283.15 | 0 |  | -2.5 | ([Martell et al., 2004](#_ENREF_41)) |
|  | 285.65 | 0 | -45.187 | -2.259 | ([Sóvágó et al., 1993](#_ENREF_63)), ([Christensen et al., 1976](#_ENREF_15)) |
|  | 298.15 | 0 | -44.936 | -0.335 | ([Sóvágó et al., 1993](#_ENREF_63)), ([Christensen et al., 1976](#_ENREF_15)) |
|  | 298.15 | 0 | -44.72 | -0.71 | ([Martell et al., 2004](#_ENREF_41)) |
|  | 298.15 | 0 | -44.51 | ⎯ | ([Sóvágó et al., 1993](#_ENREF_63)) |
|  | 298.15 | 0 | -44.9 | ⎯ | ([Sóvágó et al., 1993](#_ENREF_63)) |
|  | 298.15 | 0 | ⎯ | -5.36 | ([Pettit and Powell, 2001](#_ENREF_51)) |
|  | 310.65 | 0 | -44.183 | 1.925 | ([Sóvágó et al., 1993](#_ENREF_63)), ([Christensen et al., 1976](#_ENREF_15)) |
|  | 313.15 | 0 | -43.9 | ⎯ | ([Martell et al., 2004](#_ENREF_41)) |
|  | 323.15 | 0 | -42.802 | 4.602 | ([Sóvágó et al., 1993](#_ENREF_63)), ([Christensen et al., 1976](#_ENREF_15)) |
|  | 293.15 | 0.010 | -43.388 | ⎯ | ([Christensen et al., 1976](#_ENREF_15)) |
| Unknown | 298.15 | 0.100 | -44.73 | ⎯ | ([Sóvágó et al., 1993](#_ENREF_63)), ([Christensen et al., 1976](#_ENREF_15)) |
| Unknown | 298.15 | 1.000 | -47.44 | ⎯ | ([Martell et al., 2004](#_ENREF_41)) |
| KNO_3_ | 298.15 | 0.100 | -45.98 | ⎯ | ([Christensen et al., 1976](#_ENREF_15)) |
| KNO_3_ | 298.15 | 0.100 | -44.7 | ⎯ | ([Pettit and Powell, 2001](#_ENREF_51)) |
| KNO_3_ | 298.15 | 0.160 | -45.90 | ⎯ | ([Sóvágó et al., 1993](#_ENREF_63)), ([Christensen et al., 1976](#_ENREF_15)) |
| NaClO_4_ | 298.15 | 0.100 | -47.3 | 0.35 | ([Pettit and Powell, 2001](#_ENREF_51)) |
| NaClO_4_ | 298.15 | 1.000 | -47.46 | ⎯ | ([Sóvágó et al., 1993](#_ENREF_63)) |

**Table 3S** Literature values for the protonation of L-Serine at different temperatures, ionic strengths and in different ionic media

| Ionic medium | *T* / K | *I* / mol dm^-3^ | log $\text{K}_{\text{1}}^{\text{H}}$ | log $\text{K}_{\text{2}}^{\text{H}}$ | Ref. |
| --- | --- | --- | --- | --- | --- |
| Unknown | 310.15 | 0.150 | 8.72 | 2.09 | Recommended values ([Berthon, 1995](#_ENREF_7)) |
| KCl | 298.15 | 0.050 | 9.09 | 2.16 | Tentative values ([Berthon, 1995](#_ENREF_7)) |
| KNO_3_ | 298.15 | 0.100 | 9.05 | 2.1 | Tentative values ([Berthon, 1995](#_ENREF_7)) |
| NaClO_4_ | 298.15 | 3.000 | 9.53 | 2.5 | Tentative values ([Berthon, 1995](#_ENREF_7)) |
|  | 274.15 | 0 | 9.880 | 2.296 | ([Berthon, 1995](#_ENREF_7)) |
|  | 285.65 | 0 | 9.542 | 2.232 | ([Berthon, 1995](#_ENREF_7)) |
|  | 298.15 | 0 | 9.208 | 2.186 | ([Berthon, 1995](#_ENREF_7)) |
|  | 310.65 | 0 | 8.904 | 2.154 | ([Berthon, 1995](#_ENREF_7)) |
|  | 323.15 | 0 | 8.628 | 2.132 | ([Berthon, 1995](#_ENREF_7)) |
|  | 298.15 | 0 | 9.15 | ⎯ | ([Berthon, 1995](#_ENREF_7)) |
|  | 293.15 | 0 | 9.34 | ⎯ | ([Pettit and Powell, 2001](#_ENREF_51)) |
|  | 298.15 | 0 | 9.28 | 2.18 | ([Pettit and Powell, 2001](#_ENREF_51)) |
|  | 298.15 | 0 | 8.99 | 2.23 | ([Pettit and Powell, 2001](#_ENREF_51)) |
|  | 285.15 | 0 | 9.542 | 2.232 | ([Martell et al., 2004](#_ENREF_41)) |
|  | 298.15 | 0 | 9.209 | 2.187 | ([Martell et al., 2004](#_ENREF_41)) |
|  | 310.15 | 0 | 8.904 | 2.154 | ([Martell et al., 2004](#_ENREF_41)) |
|  | 293.15 | 0.010 | 9.24 | 2.20 | ([Berthon, 1995](#_ENREF_7)), ([Pettit and Powell, 2001](#_ENREF_51)) |
|  | 293.15 | 0.010 | 9.34 | ⎯ | ([Berthon, 1995](#_ENREF_7)) |
|  | 283.15 | 0.050 | 9.57 | 2.27 | ([Berthon, 1995](#_ENREF_7)) |
|  | 292.65 | 0.050 | 9.35 | 2.23 | ([Berthon, 1995](#_ENREF_7)) |
|  | 298.15 | 0.050 | 9.15 | 2.21 | ([Berthon, 1995](#_ENREF_7)) |
|  | 303.15 | 0.050 | 9.02 | 2.19 | ([Berthon, 1995](#_ENREF_7)) |
|  | 313.15 | 0.050 | 8.78 | 2.17 | ([Berthon, 1995](#_ENREF_7)) |
| Unknown | 298.15 | 0.100 | 9.05 | 2.16 | ([Martell et al., 2004](#_ENREF_41)) |
| Unknown | 308.15 | 0.100 | 9.0 | 2.1 | ([Berthon, 1995](#_ENREF_7)) |
| Unknown | 310.15 | 0.150 | 8.72 | 2.11 | ([Martell et al., 2004](#_ENREF_41)) |
| Unknown | 298.15 | 0.500 | 9.00 | 2.20 | ([Martell et al., 2004](#_ENREF_41)) |
| Unknown | 298.15 | 1.000 | 9.00 | 2.19 | ([Martell et al., 2004](#_ENREF_41)) |
| NaCl | 298.15 | 0.100 | 9.24 | ⎯ | ([Berthon, 1995](#_ENREF_7)) |
| NaCl | 293.15 | 0.150 | 9.18 | ⎯ | ([Berthon, 1995](#_ENREF_7)) |
| NaCl | 310.15 | 0.150 | 8.708 | 2.107 | ([Berthon, 1995](#_ENREF_7)) |
| NaCl | 298.15 | 0.150 | 8.954 | 2.209 | ([Berthon, 1995](#_ENREF_7)) |
| NaCl | 310.15 | 0.150 | 8.728 | 2.16 | ([Pettit and Powell, 2001](#_ENREF_51)) |
| NaCl | 293.15 | 0.150 | 9.18 | 1.47 | ([Pettit and Powell, 2001](#_ENREF_51)) |
| NaCl | 298.15 | 0.150 | 8.95 | 2.21 | ([Pettit and Powell, 2001](#_ENREF_51)) |
| NaCl | 298.15 | 0.250 | 9.03 | 2.173 | ([Berthon, 1995](#_ENREF_7)), ([Pettit and Powell, 2001](#_ENREF_51)) |
| NaCl | 298.15 | 3.000 | 9.50 | 2.45 | ([Martell et al., 2004](#_ENREF_41)), ([Berthon, 1995](#_ENREF_7)), ([Pettit and Powell, 2001](#_ENREF_51)) |
| KCl | 29315 | 0.050 | 9.23 | 2.14 | ([Berthon, 1995](#_ENREF_7)) |
| KCl | 298.15 | 0.050 | 9.10 | 2.15 | ([Berthon, 1995](#_ENREF_7)), ([Pettit and Powell, 2001](#_ENREF_51)) |
| KCl | 298.15 | 0.050 | 9.08 | 2.17 | ([Berthon, 1995](#_ENREF_7)), ([Pettit and Powell, 2001](#_ENREF_51)) |
| KCl | 303.15 | 0.050 | 8.97 | 2.18 | ([Berthon, 1995](#_ENREF_7)) |
| KCl | 308.15 | 0.050 | 8.83 | 2.22 | ([Berthon, 1995](#_ENREF_7)) |
| KCl | 293.15 | 0.100 | 9.0 | ⎯ | ([Berthon, 1995](#_ENREF_7)) |
| KCl | 295.15 | 0.100 | 9.21 | ⎯ | ([Berthon, 1995](#_ENREF_7)) |
| KCl | 298.15 | 0.100 | 8.75 | ⎯ | ([Pettit and Powell, 2001](#_ENREF_51)) |
| KCl | 298.15 | 0.100 | 9.260 | ⎯ | ([Berthon, 1995](#_ENREF_7)) |
| KCl | 310.15 | 0.100 | 8.977 | ⎯ | ([Berthon, 1995](#_ENREF_7)) |
| KCl | 323.15 | 0.100 | 8.685 | ⎯ | ([Berthon, 1995](#_ENREF_7)) |
| KCl | 298.15 | 0.150 | 9.02 | 2.12 | ([Berthon, 1995](#_ENREF_7)) |
| KCl | 298.15 | 0.200 | 9.04 | 2.13 | ([Berthon, 1995](#_ENREF_7)), ([Pettit and Powell, 2001](#_ENREF_51)) |
| KCl | 298.15 | 0.200 | 9.02 | 2.16 | ([Pettit and Powell, 2001](#_ENREF_51)) |
| KCl | 298.15 | 0.200 | 9.38 | 2.44 | ([Berthon, 1995](#_ENREF_7)), ([Pettit and Powell, 2001](#_ENREF_51)) |
| KCl | 293.15 | 1.000 | 9.12 | ⎯ | ([Berthon, 1995](#_ENREF_7)), ([Pettit and Powell, 2001](#_ENREF_51)) |
| KCl | 298.15 | 1.000 | 9.24 | ⎯ | ([Berthon, 1995](#_ENREF_7)) |
| KCl | 298.15 | 1.000 | 9.15 | ⎯ | ([Berthon, 1995](#_ENREF_7)) |
| KCl | 298.15 | 3.000 | 9.452 | 2.405 | ([Berthon, 1995](#_ENREF_7)) |
| KCl | 298.15 | 3.000 | 9.45 | 2.41 | ([Martell et al., 2004](#_ENREF_41)) |
| NaNO_3_ | 298.15 | 0.100 | 9.17 | ⎯ | ([Berthon, 1995](#_ENREF_7)) |
| NaNO_3_ | 303.15 | 0.200 | 8.95 | 2.25 | ([Berthon, 1995](#_ENREF_7)), ([Pettit and Powell, 2001](#_ENREF_51)) |
| NaNO_3_ | 298.15 | 1.000 | 8.97 | 2.19 | ([Berthon, 1995](#_ENREF_7)) |
| NaNO_3_ | 298.15 | 2.000 | 9.27 | 2.32 | ([Martell et al., 2004](#_ENREF_41)) |
| NaNO_3_ | 298.15 | 2.250 | 9.274 | 2.326 | ([Pettit and Powell, 2001](#_ENREF_51)) |
| NaNO_3_ | 298.15 | 2.250 | 9.274 | 2.322 | ([Berthon, 1995](#_ENREF_7)) |
| KNO_3_ | 298.15 | 0.100 | 9.20 | ⎯ | ([Pettit and Powell, 2001](#_ENREF_51)) |
| KNO_3_ | 298.15 | 0.100 | 9.25 | ⎯ | ([Pettit and Powell, 2001](#_ENREF_51)) |
| KNO_3_ | 298.15 | 0.100 | 9.05 | 2.10 | ([Pettit and Powell, 2001](#_ENREF_51)) |
| KNO_3_ | 298.15 | 0.100 | 9.074 | 1.95 | ([Berthon, 1995](#_ENREF_7)) |
| KNO_3_ | 298.15 | 0.100 | 9.073 | 1.951 | ([Berthon, 1995](#_ENREF_7)) |
| KNO_3_ | 298.15 | 0.100 | 9.16 | 2.349 | ([Berthon, 1995](#_ENREF_7)) |
| KNO_3_ | 298.15 | 0.100 | 9.15 | ⎯ | ([Berthon, 1995](#_ENREF_7)) |
| KNO_3_ | 298.15 | 0.100 | 9.3 | ⎯ | ([Pettit and Powell, 2001](#_ENREF_51)) |
| KNO_3_ | 298.15 | 0.100 | 9.24 | ⎯ | ([Pettit and Powell, 2001](#_ENREF_51)) |
| KNO_3_ | 298.15 | 0.100 | 9.25 | 1.89 | ([Pettit and Powell, 2001](#_ENREF_51)) |
| KNO_3_ | 298.15 | 0.100 | 9.249 | 1.891 | ([Pettit and Powell, 2001](#_ENREF_51)) |
| KNO_3_ | 298.15 | 0.100 | 9.15 | ⎯ | ([Pettit and Powell, 2001](#_ENREF_51)) |
| KNO_3_ | 298.15 | 0.100 | 9.05 | ⎯ | ([Berthon, 1995](#_ENREF_7)), ([Pettit and Powell, 2001](#_ENREF_51)) |
| KNO_3_ | 298.15 | 0.100 | 9.16 | 2.35 | ([Pettit and Powell, 2001](#_ENREF_51)) |
| KNO_3_ | 298.15 | 0.100 | 9.17 | 2.27 | ([Berthon, 1995](#_ENREF_7)), ([Pettit and Powell, 2001](#_ENREF_51)) |
| KNO_3_ | 298.15 | 0.100 | 9.073 | 1.947 | ([Pettit and Powell, 2001](#_ENREF_51)) |
| KNO_3_ | 298.15 | 0.100 | 9.07 | 1.95 | ([Pettit and Powell, 2001](#_ENREF_51)) |
| KNO_3_ | 298.15 | 0.100 | 9.14 | ⎯ | ([Berthon, 1995](#_ENREF_7)), ([Pettit and Powell, 2001](#_ENREF_51)) |
| KNO_3_ | 298.15 | 0.100 | 9.15 | 2.55 | ([Berthon, 1995](#_ENREF_7)), ([Pettit and Powell, 2001](#_ENREF_51)) |
| KNO_3_ | 293.15 | 0.100 | 9.18 | ⎯ | ([Berthon, 1995](#_ENREF_7)), ([Pettit and Powell, 2001](#_ENREF_51)) |
| KNO_3_ | 303.15 | 0.100 | 8.95 | ⎯ | ([Berthon, 1995](#_ENREF_7)), ([Pettit and Powell, 2001](#_ENREF_51)) |
| KNO_3_ | 313.15 | 0.100 | 8.78 | ⎯ | ([Berthon, 1995](#_ENREF_7)), ([Pettit and Powell, 2001](#_ENREF_51)) |
| KNO_3_ | 323.15 | 0.100 | 8.57 | ⎯ | ([Berthon, 1995](#_ENREF_7)), ([Pettit and Powell, 2001](#_ENREF_51)) |
| KNO_3_ | 333.15 | 0.100 | 8.38 | ⎯ | ([Berthon, 1995](#_ENREF_7)), ([Pettit and Powell, 2001](#_ENREF_51)) |
| KNO_3_ | 298.15 | 0.150 | 9.14 | ⎯ | ([Berthon, 1995](#_ENREF_7)), ([Pettit and Powell, 2001](#_ENREF_51)) |
| KNO_3_ | 310.15 | 0.150 | 8.72 | 2.09 | ([Pettit and Powell, 2001](#_ENREF_51)) |
| KNO_3_ | 310.15 | 0.150 | 8.839 | 2.180 | ([Berthon, 1995](#_ENREF_7)) |
| KNO_3_ | 310.15 | 0.150 | 8.841 | 2.180 | ([Berthon, 1995](#_ENREF_7)) |
| KNO_3_ | 310.15 | 0.150 | 8.84 | 2.18 | ([Pettit and Powell, 2001](#_ENREF_51)) |
| KNO_3_ | 298.15 | 0.160 | 9.18 | ⎯ | ([Berthon, 1995](#_ENREF_7)) |
| KNO_3_ | 288.15 | 0.200 | 9.34 | 2.30 | ([Berthon, 1995](#_ENREF_7)) |
| KNO_3_ | 298.15 | 0.200 | 9.12 | 2.29 | ([Berthon, 1995](#_ENREF_7)) |
| KNO_3_ | 298.15 | 0.200 | 9.04 | 2.25 | ([Berthon, 1995](#_ENREF_7)) |
| KNO_3_ | 308.15 | 0.200 | 8.70 | 2.20 | ([Pettit and Powell, 2001](#_ENREF_51)) |
| KNO_3_ | 313.15 | 0.200 | 8.78 | 2.27 | ([Berthon, 1995](#_ENREF_7)), ([Pettit and Powell, 2001](#_ENREF_51)) |
| KNO_3_ | 303.15 | 0.250 | 8.81 | 2.17 | ([Berthon, 1995](#_ENREF_7)), ([Pettit and Powell, 2001](#_ENREF_51)) |
| KNO_3_ | 303.15 | 0.500 | 9.18 | 2.21 | ([Berthon, 1995](#_ENREF_7)) |
| KNO_3_ | 293.15 | 0.500 | 9.26 | 2.80 | ([Berthon, 1995](#_ENREF_7)) |
| KNO_3_ | 298.15 | 0.500 | 9.06 | ⎯ | ([Berthon, 1995](#_ENREF_7)), ([Pettit and Powell, 2001](#_ENREF_51)) |
| KNO_3_ | 298.15 | 1.000 | 9.118 | 2.289 | ([Pettit and Powell, 2001](#_ENREF_51)) |
| KNO_3_ | 298.15 | 1.000 | 9.12 | 2.24 | ([Pettit and Powell, 2001](#_ENREF_51)) |
| KNO_3_ | 303.15 | 1.000 | 9.10 | ⎯ | ([Berthon, 1995](#_ENREF_7)) |
| KNO_3_ | 303.15 | 1.000 | 9.11 | ⎯ | ([Pettit and Powell, 2001](#_ENREF_51)) |
| KNO_3_ | 298.15 | 3.000 | 9.53 | 2.5 | ([Pettit and Powell, 2001](#_ENREF_51)) |
| LiClO_4_ | 298.15 | 0.100 | 9.01 | 2.12 | ([Berthon, 1995](#_ENREF_7)) |
| NaClO_4_ | 293.15 | 0.100 | 9.30 | 2.74 | ([Pettit and Powell, 2001](#_ENREF_51)) |
| NaClO_4_ | 298.15 | 0.100 | 9.02 | 2.29 | ([Berthon, 1995](#_ENREF_7)) |
| NaClO_4_ | 298.15 | 0.100 | 9.05 | 2.29 | ([Pettit and Powell, 2001](#_ENREF_51)) |
| NaClO_4_ | 298.15 | 0.100 | 9.171 | 2.211 | ([Berthon, 1995](#_ENREF_7)), ([Pettit and Powell, 2001](#_ENREF_51)) |
| NaClO_4_ | 310.15 | 0.150 | 8.712 | 2.078 | ([Pettit and Powell, 2001](#_ENREF_51)) |
| NaClO_4_ | 310.15 | 0.150 | 8.728 | 2.158 | ([Berthon, 1995](#_ENREF_7)) |
| NaClO_4_ | 310.15 | 0.150 | 8.712 | 2.081 | ([Berthon, 1995](#_ENREF_7)) |
| NaClO_4_ | 298.15 | 0.200 | 9.09 | 2.16 | ([Pettit and Powell, 2001](#_ENREF_51)) |
| NaClO_4_ | 293.15 | 0.700 | 8.98 | ⎯ | ([Berthon, 1995](#_ENREF_7)) |
| NaClO_4_ | 298.15 | 0.700 | 8.98 | ⎯ | ([Berthon, 1995](#_ENREF_7)) |
| NaClO_4_ | 298.15 | 1.000 | 9.18 | 2.27 | ([Berthon, 1995](#_ENREF_7)) |
| NaClO_4_ | 298.15 | 1.000 | 9.20 | ⎯ | ([Pettit and Powell, 2001](#_ENREF_51)) |
| NaClO_4_ | 293.15 | 1.000 | 9.12 | 2.26 | ([Berthon, 1995](#_ENREF_7)), ([Pettit and Powell, 2001](#_ENREF_51)) |
| NaClO_4_ | 298.15 | 3.000 | 9.53 | 2.5 | ([Berthon, 1995](#_ENREF_7)) |
| NaClO_4_ | 298.15 | 3.000 | 9.64 | 2.54 | ([Martell et al., 2004](#_ENREF_41)) |
| NaClO_4_ | 298.15 | 3.000 | 9.61 | 2.60 | ([Berthon, 1995](#_ENREF_7)) |
| NaClO_4_ | 298.15 | 3.000 | 9.72 | 2.48 | ([Pettit and Powell, 2001](#_ENREF_51)) |
| NaClO_4_ | 298.15 | 3.000 | 9.57 | 2.56 | ([Pettit and Powell, 2001](#_ENREF_51)) |
| NaClO_4_ | 298.15 | 3.000 | 9.574 | 2.559 | ([Berthon, 1995](#_ENREF_7)) |
| KH_2_PO_4_ | 298.15 | 0.060 | 9.14 | ⎯ | ([Berthon, 1995](#_ENREF_7)), ([Pettit and Powell, 2001](#_ENREF_51)) |
|  |  |  |  |  |  |
| Ionic medium | *T* / K | *I* / mol dm^-3^ | Δ$\text{H}_{\text{1}}^{\text{0}}$ / kJ mol^-1^ | Δ$\text{H}_{\text{2}}^{\text{0}}$ / kJ mol^-1^ | Ref. |
|  | 274.15 | 0 | -43.72 | -8.29 | ([Berthon, 1995](#_ENREF_7)), ([Christensen et al., 1976](#_ENREF_15)) |
|  | 285.65 | 0 | -43.89 | -7.20 | ([Berthon, 1995](#_ENREF_7)), ([Christensen et al., 1976](#_ENREF_15)) |
|  | 298.15 | 0 | -40.6 | -1.34 | ([Pettit and Powell, 2001](#_ENREF_51)), ([Berthon, 1995](#_ENREF_7)) |
|  | 298.15 | 0 | -43.3 | -5.52 | ([Christensen et al., 1976](#_ENREF_15)) |
|  | 298.15 | 0 | -43.53 | -5.71 | ([Berthon, 1995](#_ENREF_7)), ([Christensen et al., 1976](#_ENREF_15)) |
|  | 298.15 | 0 | -40.6 | -1.3 | ([Pettit and Powell, 2001](#_ENREF_51)) |
|  | 298.15 | 0 | -40.63 | ⎯ | ([Pettit and Powell, 2001](#_ENREF_51)) |
|  | 298.15 | 0 | -45.5 | -3.99 | ([Pettit and Powell, 2001](#_ENREF_51)) |
|  | 310.65 | 0 | -42.68 | -3.90 | ([Berthon, 1995](#_ENREF_7)), ([Christensen et al., 1976](#_ENREF_15)) |
|  | 323.15 | 0 | -41.17 | -1.72 | ([Berthon, 1995](#_ENREF_7)), ([Christensen et al., 1976](#_ENREF_15)) |
| Unknown | 293.15 | 0.010 | -41.5 | ⎯ | ([Berthon, 1995](#_ENREF_7)), ([Christensen et al., 1976](#_ENREF_15)) |
| Unknown | 298.15 | 0.100 | -43.0 | -3 | ([Martell et al., 2004](#_ENREF_41)) |
| KCl | 298.15 | 0.050 | -42.68 | -5.02 | ([Berthon, 1995](#_ENREF_7)), ([Christensen et al., 1976](#_ENREF_15)) |
| KCl | 298.15 | 0.100 | -42.42 | ⎯ | ([Berthon, 1995](#_ENREF_7)), ([Christensen et al., 1976](#_ENREF_15)) |
| KCl | 298.15 | 0.200 | -43.6 | -1.9 | ([Pettit and Powell, 2001](#_ENREF_51)), ([Berthon, 1995](#_ENREF_7)) |
| KNO_3_ | 298.15 | 0.100 | -43.2 | -2 | ([Pettit and Powell, 2001](#_ENREF_51)) |
| KNO_3_ | 298.15 | 0.100 | -42.3 | ⎯ | ([Pettit and Powell, 2001](#_ENREF_51)) |
| KNO_3_ | 298.15 | 0.100 | -43.76 | ⎯ | ([Berthon, 1995](#_ENREF_7)), ([Christensen et al., 1976](#_ENREF_15)) |
| KNO_3_ | 298.15 | 0.100 | -43.28 | ⎯ | ([Berthon, 1995](#_ENREF_7)), ([Christensen et al., 1976](#_ENREF_15)) |
| KNO_3_ | 298.15 | 0.160 | -42.05 | ⎯ | ([Berthon, 1995](#_ENREF_7)), ([Christensen et al., 1976](#_ENREF_15)) |
| NaClO_4_ | 298.15 | 0.100 | -45.5 | -3.99 | ([Berthon, 1995](#_ENREF_7)) |

**Table 4S** Literature values for the protonation of L-Phenylalanine at different temperatures, ionic strengths and in different ionic media

| Ionic medium | *T* / K | *I* / mol dm^-3^ | log $\text{K}_{\text{1}}^{\text{H}}$ | log $\text{K}_{\text{2}}^{\text{H}}$ | Ref. |
| --- | --- | --- | --- | --- | --- |
|  | 273.15 | 0 | 9.95 | 2.28 | ([Pettit, 1984](#_ENREF_50)) |
|  | 283.15 | 0 | 9.66 | 2.21 | ([Pettit, 1984](#_ENREF_50)) |
|  | 283.15 | 0 | 9.75 | ⎯ | ([Martell et al., 2004](#_ENREF_41)), ([Christensen et al., 1976](#_ENREF_15)) |
|  | 283.15 | 0 | 9.75 | 2.14 | ([Pettit, 1984](#_ENREF_50)) |
|  | 293.15 | 0 | 9.38 | 2.20 | ([Pettit, 1984](#_ENREF_50)) |
|  | 293.15 | 0.005 | 9.33 | ⎯ | ([Pettit, 1984](#_ENREF_50)) |
|  | 293.15 | 0.010 | 9.31 | 2.04 | ([Pettit, 1984](#_ENREF_50)) |
|  | 298.15 | 0 | 9.31 | 2.20 | ([Martell et al., 2004](#_ENREF_41)), ([Pettit, 1984](#_ENREF_50)) |
|  | 298.15 | 0 | 9.31 | ⎯ | ([Christensen et al., 1976](#_ENREF_15)) |
|  | 303.15 | 0 | 9.15 | 2.23 | ([Pettit, 1984](#_ENREF_50)) |
|  | 313.15 | 0 | 8.89 | 2.20 | ([Pettit, 1984](#_ENREF_50)) |
|  | 313.15 | 0 | 8.96 | ⎯ | ([Christensen et al., 1976](#_ENREF_15)) |
|  | 313.15 | 0 | 8.96 | 2.21 | ([Pettit, 1984](#_ENREF_50)) |
| Unknown | 298.15 | 0.100 | 9.09 | 2.18 | ([Martell et al., 2004](#_ENREF_41)) |
| Unknown | 310.15 | 0.150 | 8.76 | 2.15 | ([Martell et al., 2004](#_ENREF_41)) |
| Unknown | 298.15 | 0.500 | 9.05 | 2.29 | ([Martell et al., 2004](#_ENREF_41)) |
| Unknown | 298.15 | 1.000 | 9.16 | 2.28 | ([Martell et al., 2004](#_ENREF_41)) |
| NaCl | 296.15 | 0.100 | 9.23 | 2.21 | ([Pettit and Powell, 2001](#_ENREF_51)) |
| NaCl | 297.15 | 0.100 | 9.12 | 2.16 | ([Pettit, 1984](#_ENREF_50)) |
| NaCl | 298.15 | 0.100 | 9.15 | 2.16 | ([Pettit, 1984](#_ENREF_50)) |
| NaCl | 298.15 | 0.150 | 9.16 | 2.00 | ([Pettit and Powell, 2001](#_ENREF_51)) |
| NaCl | 310.15 | 0.150 | 8.772 | 2.16 | ([Pettit and Powell, 2001](#_ENREF_51)) |
| KNO_3_ | 293.15 | 0.100 | 9.25 | 2.39 | ([Pettit, 1984](#_ENREF_50)) |
| KNO_3_ | 298.15 | 0.100 | 8.90 | 2.50 | ([Pettit and Powell, 2001](#_ENREF_51)) |
| KNO_3_ | 298.15 | 0.100 | 9.19 | 2.26 | ([Pettit, 1984](#_ENREF_50)) |
| KNO_3_ | 298.15 | 0.100 | 9.15 | 1.81 | ([Pettit, 1984](#_ENREF_50)) |
| KNO_3_ | 298.15 | 0.100 | 9.09 | ⎯ | ([Pettit, 1984](#_ENREF_50)) |
| KNO_3_ | 298.15 | 0.100 | 9.06 | 2.17 | ([Pettit and Powell, 2001](#_ENREF_51)) |
| KNO_3_ | 303.15 | 0.100 | 9.03 | 2.37 | ([Pettit, 1984](#_ENREF_50)) |
| KNO_3_ | 308.15 | 0.100 | 8.59 | ⎯ | ([Pettit and Powell, 2001](#_ENREF_51)) |
| KNO_3_ | 313.15 | 0.100 | 8.80 | 2.33 | ([Pettit, 1984](#_ENREF_50)) |
| KNO_3_ | 323.15 | 0.100 | 8.57 | 2.31 | ([Pettit, 1984](#_ENREF_50)) |
| KNO_3_ | 333.15 | 0.100 | 8.45 | 2.39 | ([Pettit, 1984](#_ENREF_50)) |
| KNO_3_ | 298.15 | 0.150 | 9.22 | ⎯ | ([Pettit and Powell, 2001](#_ENREF_51)) |
| KNO_3_ | 298.15 | 0.160 | 9.02 | ⎯ | ([Pettit, 1984](#_ENREF_50)) |
| KNO_3_ | 298.15 | 0.160 | 9.02 | ⎯ | ([Christensen et al., 1976](#_ENREF_15)) |
| KNO_3_ | 308.15 | 0.200 | 9.32 | 2.28 | ([Pettit and Powell, 2001](#_ENREF_51)) |
| KNO_3_ | 308.15 | 0.200 | 8.73 | 2.12 | ([Pettit and Powell, 2001](#_ENREF_51)) |
| KNO_3_ | 298.15 | 0.500 | 9.06 | 2.27 | ([Pettit, 1984](#_ENREF_50)) |
| KNO_3_ | 298.15 | 0.500 | 9.05 | 2.32 | ([Pettit, 1984](#_ENREF_50)) |
| KNO_3_ | 298.15 | 0.700 | 9.04 | 1.95 | ([Pettit and Powell, 2001](#_ENREF_51)) |
| KCl | 293.15 | 0.05 | 9.20 | 2.09 | ([Pettit, 1984](#_ENREF_50)) |
| KCl | 298.15 | 0.050 | 9.11 | 2.20 | ([Pettit, 1984](#_ENREF_50)) |
| KCl | 298.15 | 0.050 | 9.08 | 2.09 | ([Pettit, 1984](#_ENREF_50)) |
| KCl | 303.15 | 0.050 | 8.97 | 2.09 | ([Pettit, 1984](#_ENREF_50)) |
| KCl | 308.15 | 0.050 | 8.85 | 2.09 | ([Pettit, 1984](#_ENREF_50)) |
| KCl | 278.15 | 0.100 | 9.759 | ⎯ | ([Christensen et al., 1976](#_ENREF_15)) |
| KCl | 293.15 | 0.100 | 8.4 | ⎯ | ([Pettit, 1984](#_ENREF_50)) |
| KCl | 293.15 | 0.100 | 9.04 | ⎯ | ([Pettit, 1984](#_ENREF_50)) |
| KCl | 298.15 | 0.100 | 9.08 | 2.18 | ([Pettit and Powell, 2001](#_ENREF_51)) |
| KCl | 298.15 | 0.100 | 9.220 | ⎯ | ([Christensen et al., 1976](#_ENREF_15)) |
| KCl | 298.15 | 0.100 | 9.1 | 1.80 | ([Pettit, 1984](#_ENREF_50)) |
| KCl | 308.15 | 0.100 | 8.89 | 1.59 | ([Pettit, 1984](#_ENREF_50)) |
| KCl | 318.15 | 0.100 | 8.52 | 1.22 | ([Pettit, 1984](#_ENREF_50)) |
| KCl | 318.15 | 0.100 | 8.670 | ⎯ | ([Christensen et al., 1976](#_ENREF_15)) |
| NaClO_4_ | 298.15 | 0.100 | 9.21 | 1.92 | ([Pettit and Powell, 2001](#_ENREF_51)) |
| NaClO_4_ | 298.15 | 0.200 | 9.214 | 2.04 | ([Pettit and Powell, 2001](#_ENREF_51)) |
| NaClO_4_ | 298.15 | 0.200 | 9.214 | 1.94 | ([Pettit and Powell, 2001](#_ENREF_51)) |
| NaClO_4_ | 298.15 | 0.200 | 8.80 | 2.20 | ([Pettit and Powell, 2001](#_ENREF_51)) |
| NaClO_4_ | 298.15 | 0.200 | 9.21 | 1.92 | ([Pettit and Powell, 2001](#_ENREF_51)), ([Martell et al., 2004](#_ENREF_41)) |
| NaClO_4_ | 303.15 | 0.200 | 8.98 | 3.11 | ([Pettit and Powell, 2001](#_ENREF_51)) |
| NaClO_4_ | 303.15 | 0.200 | 9.21 | 1.92 | ([Pettit and Powell, 2001](#_ENREF_51)) |
| NaClO_4_ | 293.15 | 1.000 | 9.18 | 2.21 | ([Pettit, 1984](#_ENREF_50)) |
| NaClO_4_ | 298.15 | 3.000 | 9.61 | 2.75 | ([Martell et al., 2004](#_ENREF_41)). ([Pettit, 1984](#_ENREF_50)), ([Christensen et al., 1976](#_ENREF_15)) |
| NaNO_3_ | 298.15 | 0.100 | 9.14 | 2.55 | ([Pettit and Powell, 2001](#_ENREF_51)) |
| NaNO_3_ | 298.15 | 0.100 | 8.98 | 2.36 | ([Pettit and Powell, 2001](#_ENREF_51)) |
| NaNO_3_ | 293.15 | 0.370 | 9.35 | 2.41 | ([Pettit, 1984](#_ENREF_50)) |
|  |  |  |  |  |  |
| Ionic medium | *T* / K | *I* / mol dm^-3^ | Δ$\text{H}_{\text{1}}^{\text{0}}$ / kJ mol^-1^ | Δ$\text{H}_{\text{2}}^{\text{0}}$ / kJ mol^-1^ | Ref. |
|  | 283.15 | 0 | -47.6 | ⎯ | ([Martell et al., 2004](#_ENREF_41)), ([Christensen et al., 1976](#_ENREF_15)) |
|  | 298.15 | 0 | -44.7 | -2 | ([Martell et al., 2004](#_ENREF_41)) |
|  | 283.15 | 0 | -47.7 | ⎯ | ([Pettit, 1984](#_ENREF_50)) |
|  | 298.15 | 0 | -44.6 | ⎯ | ([Christensen et al., 1976](#_ENREF_15)) |
|  | 298.15 | 0 | -45.56 | -0.59 | ([Pettit and Powell, 2001](#_ENREF_51)) |
|  | 298.15 | 0 | -45.65 | ⎯ | ([Pettit and Powell, 2001](#_ENREF_51)) |
|  | 298.15 | 0 | -44.6 | ⎯ | ([Pettit, 1984](#_ENREF_50)) |
|  | 313.15 | 0 | -43.9 | ⎯ | ([Martell et al., 2004](#_ENREF_41)) |
|  | 313.15 | 0 | -44.1 | ⎯ | ([Pettit, 1984](#_ENREF_50)) |
|  | 293.15 | 0.010 | -42.3 | ⎯ | ([Christensen et al., 1976](#_ENREF_15)) |
| Unknown | 298.15 | 0.100 | -44.7 | -2 | ([Martell et al., 2004](#_ENREF_41)) |
| Unknown | 298.15 | 1.000 | -46.4 | -3 | ([Martell et al., 2004](#_ENREF_41)) |
| NaCl | 298.15 | 0.150 | -24.09 | ⎯ | ([Pettit and Powell, 2001](#_ENREF_51)) |
| KCl | 278.15 | 0.100 | -39.6 | ⎯ | ([Christensen et al., 1976](#_ENREF_15)) |
| KCl | 298.15 | 0.100 | -46.3 | ⎯ | ([Christensen et al., 1976](#_ENREF_15)) |
| KCl | 318.15 | 0.100 | -53.6 | ⎯ | ([Christensen et al., 1976](#_ENREF_15)) |
| KNO_3_ | 298.15 | 0.160 | -43.2 | ⎯ | ([Pettit, 1984](#_ENREF_50)), ([Christensen et al., 1976](#_ENREF_15)) |
| NaClO_4_ | 298.15 | 3.000 | -50.4 | -9.7 | ([Martell et al., 2004](#_ENREF_41)). ([Pettit, 1984](#_ENREF_50)), ([Christensen et al., 1976](#_ENREF_15)) |

**Table 5S** Literature values of the protonation of L-Alanine at different temperatures, ionic strengths and in different ionic media

| Ionic medium | *T* / K | *I* / mol dm^-3^ | log $\text{K}_{\text{1}}^{\text{H}}$ | log $\text{K}_{\text{2}}^{\text{H}}$ | Ref. |
| --- | --- | --- | --- | --- | --- |
| KNO_3_ | 298.15 | 0.296 | 9.70 | 2.41 | ([Brandariz et al., 1993](#_ENREF_10)) |
| KNO_3_ | 298.15 | 0.498 | 9.67 | 2.37 | ([Brandariz et al., 1993](#_ENREF_10)) |
| KNO_3_ | 298.15 | 0.698 | 9.69 | 2.43 | ([Brandariz et al., 1993](#_ENREF_10)) |
| KNO_3_ | 298.15 | 0.893 | 9.71 | 2.46 | ([Brandariz et al., 1993](#_ENREF_10)) |
| KNO_3_ | 298.15 | 1.095 | 9.71 | 2.46 | ([Brandariz et al., 1993](#_ENREF_10)) |
| KNO_3_ | 298.15 | 1.294 | 9.73 | 2.49 | ([Brandariz et al., 1993](#_ENREF_10)) |
| KNO_3_ | 298.15 | 1.497 | 9.73 | 2.50 | ([Brandariz et al., 1993](#_ENREF_10)) |
|  | 273.15 | 0 | 11.08 |  | ([Hamborg et al., 2007](#_ENREF_30)) |
|  | 273.15 | 0 | 10.57 | 2.43 | ([Izatt et al., 1961](#_ENREF_33)) |
| KNO_3_ | 273.15 | 0.100 | 10.19 | 2.76 | ([Pettit, 1984](#_ENREF_50)) |
|  | 274.15 | 0 | 10.59 | 2.43 | ([Smith et al., 1937](#_ENREF_62)) |
| -- | 278.15 | 0 | 9.62 | 2.33 | ([Nims and Smith, 1933](#_ENREF_45)) |
|  | 278.15 | 0 | 10.92 |  | ([Hamborg et al., 2007](#_ENREF_30)) |
| NaCl | 283.15 | 0 | 10.30 | 2.39 | ([Martell et al., 2004](#_ENREF_41)) |
|  | 283.15 | 0 | 10.30 | 2.39 | ([Izatt et al., 1961](#_ENREF_33)) |
|  | 288.15 | 0 | 9.47 |  | ([Hamborg et al., 2007](#_ENREF_30)) |
|  | 288.15 | 0 | 10.29 | 2.40 | ([Anderson et al., 1966](#_ENREF_2)) |
|  | 288.15 | 0 | 10.29 | 2.60 | ([Anderson et al., 1967](#_ENREF_1)) |
| KCl | 288.15 | 0.200 | 10.01 | 2.47 | ([Sharma and Mathur, 1965](#_ENREF_60)) |
|  | 288.65 | 0 | 10.23 | 2.38 | ([Smith et al., 1937](#_ENREF_62)) |
| -- | 293.15 | 0 | 10.01 | 2.35 | ([Nims and Smith, 1933](#_ENREF_45)) |
|  | 293.15 | 0 | 10.04 | 2.37 | ([Doğan et al., 2002](#_ENREF_17)) |
|  | 293.15 | 0 | 10.04 | 2.28 | ([Gergely et al., 1971](#_ENREF_22)) |
| KCl | 293.15 | 0.050 | 9.86 | 2.28 | ([Gergely et al., 1971](#_ENREF_22)) |
| KCl | 293.15 | 0.100 | 9.84 | 2.20 | ([Gillard et al., 1966](#_ENREF_28)) |
|  | 293.15 | 0.100 | 9.93 | 2.30 | ([Irving and Pettit, 1963](#_ENREF_32)) |
|  | 293.15 | 0.100 | 9.88 |  | ([Berezina et al., 1973](#_ENREF_6)) |
|  | 293.15 | 0.150 | 9.86 |  | ([Vlasova and Davidenko, 1985](#_ENREF_67)) |
| NaNO_3_ | 293.15 | 0.370 | 9.93 | 2.54 | ([Simeon and Weber](#_ENREF_61)) |
| KNO_3_ | 293.15 | 0.500 | 9.92 | 2.91 | ([Pettit, 1984](#_ENREF_50)) |
| KCl | 293.15 | 1.000 | 9.79 | 2.48 | ([Vieles and Bonniol, 1973](#_ENREF_66)) |
| NaClO_4_ | 293.15 | 1.000 | 9.79 | 2.49 | ([Perrin, 1958](#_ENREF_48)) |
|  | 293.15 | 1.000 | 9.79 |  | ([Vieles and Bonniol, 1973](#_ENREF_66)) |
|  | 298 | 0 | 10.33 |  | ([Hamborg et al., 2007](#_ENREF_30)) |
| -- | 298.15 | 0 | 9.87 | 2.34 | ([Nims and Smith, 1933](#_ENREF_45)) |
|  | 298.15 | 0 | 10.33 |  | ([Hamborg et al., 2007](#_ENREF_30)) |
|  | 298.15 | 0 | 9.87 | 2.35 | ([Smith et al., 1937](#_ENREF_62)) |
|  | 298.15 | 0 | 9.89 | 2.37 | ([Anderson et al., 1966](#_ENREF_2)) |
|  | 298.15 | 0 | 9.89 | 2.63 | ([Anderson et al., 1967](#_ENREF_1)) |
|  | 298.15 | 0 | 9.90 | 2.28 | ([Gergely et al., 1971](#_ENREF_22)) |
| NaClO_4_ | 298.15 | 0.020 | 9.77 | 2.35 | ([Gergely et al., 1974b](#_ENREF_24)) |
| KCl | 298.15 | 0.050 | 9.72 | 2.28 | ([Gergely et al., 1971](#_ENREF_22)) |
| KCl | 298.15 | 0.050 | 9.73 | 2.33 | ([Gergely et al., 1972](#_ENREF_26)) |
| NaClO_4_ | 298.15 | 0.050 | 9.70 | 2.34 | ([Gergely et al., 1974b](#_ENREF_24)) |
| KCl | 298.15 | 0.100 | 9.86 | 2.51 | ([Kurganov et al., 1977](#_ENREF_36)) |
| KNO_3_ | 298.15 | 0.100 | 9.85 | 2.26 | ([Orenberg et al., 1980](#_ENREF_47)) |
| KNO_3_ | 298.15 | 0.100 | 9.80 | 2.33 | ([Sawhney et al., 1980](#_ENREF_57)) |
| KNO_3_ | 298.15 | 0.100 | 9.59 | 2.48 | ([Nourmand and Meissami, 1982](#_ENREF_46)) |
| KNO_3_ | 298.15 | 0.100 | 9.70 | 2.43 | ([Arena et al., 1983](#_ENREF_3)) |
| NaClO_4_ | 298.15 | 0.100 | 9.51 | 2.39 | ([Gharib et al., 2015](#_ENREF_27)) |
| NaClO_4_ | 298.15 | 0.100 | 9.83 | 2.26 | ([Griesser et al., 1970](#_ENREF_29)) |
| NaClO_4_ | 298.15 | 0.100 | 9.69 | 2.33 | ([Gergely et al., 1974b](#_ENREF_24)) |
| NaClO_4_ | 298.15 | 0.100 | 9.82 | 2.34 | ([Fischer and Sigel, 1980](#_ENREF_20)) |
|  | 298.15 | 0.100 | 9.80 |  | ([Sharma and Tandon, 1971](#_ENREF_59)) |
|  | 298.15 | 0.100 | 9.80 |  | ([Heijne and van der Linden, 1975](#_ENREF_31)) |
| NaCl | 298.15 | 0.120 | 9.67 | 2.35 | ([Malik and Singh, 1978](#_ENREF_40)) |
| NaCl | 298.15 | 0.150 | 9.65 | 2.37 | ([Pettit, 1984](#_ENREF_50)) |
| KCl | 298.15 | 0.200 | 9.77 | 2.40 | ([Sharma and Mathur, 1965](#_ENREF_60)) |
| KCl | 298.15 | 0.200 | 9.68 | 2.35 | ([Gergely and Sóvágó, 1973](#_ENREF_25)) |
| KCl | 298.15 | 0.200 | 9.68 | 2.35 | ([Gergely et al., 1974a](#_ENREF_23)) |
| KNO_3_ | 298.15 | 0.200 | 9.74 | 2.36 | ([Sóvágó et al., 1986](#_ENREF_64)) |
| NaClO_4_ | 298.15 | 0.200 | 9.66 | 2.10 | ([Pettit, 1984](#_ENREF_50)) |
| NaClO_4_ | 298.15 | 0.200 | 9.73 | 2.31 | ([Chidambaram and Bhattacharya, 1970](#_ENREF_13)) |
| NaClO_4_ | 298.15 | 0.200 | 9.67 | 2.33 | ([Gergely et al., 1974b](#_ENREF_24)) |
| NaClO_4_ | 298.15 | 0.200 |  | 2.43 | ([Sarin and Munshi, 1973](#_ENREF_56)) |
|  | 298.15 | 0.240 | 9.87 |  | ([Friedman and Levina, 1974](#_ENREF_21)) |
| KCl | 298.15 | 0.500 | 9.83 | 2.44 | ([Leussing and Hanna, 1966](#_ENREF_37)) |
| KCl | 298.15 | 0.500 | 9.81 | 2.45 | ([Felty et al., 1970](#_ENREF_19)) |
| KCl | 298.15 | 0.500 | 9.82 | 2.44 | ([Scheidegger et al., 1970](#_ENREF_58)) |
| KCl | 298.15 | 0.500 | 9.82 | 2.44 | ([Leussing and Leach, 1971](#_ENREF_38)) |
| KNO_3_ | 298.15 | 0.500 | 9.70 | 2.38 | ([Korsunov and Sergeev, 1971](#_ENREF_35)) |
| KNO_3_ | 298.15 | 0.500 | 9.77 | 2.48 | ([Lim, 1978](#_ENREF_39)) |
| NaCl | 298.15 | 0.500 | 9.67 | 2.35 | ([Martell et al., 2004](#_ENREF_41)) |
| NaClO_4_ | 298.15 | 0.500 | 9.84 | 2.39 | ([Petit-Ramel and Paris, 1968](#_ENREF_49)) |
| NaClO_4_ | 298.15 | 0.500 | 9.69 | 2.36 | ([Gergely et al., 1974b](#_ENREF_24)) |
|  | 298.15 | 0.500 | 9.75 |  | ([Heijne and van der Linden, 1975](#_ENREF_31)) |
| NaCl | 298.15 | 0.600 | 9.68 | 2.38 | ([Pettit, 1984](#_ENREF_50)) |
| KNO_3_ | 298.15 | 1.000 | 9.81 | 2.44 | ([Bonnet et al., 1972](#_ENREF_9)) |
| NaCl | 298.15 | 1.000 | 9.71 | 2.40 | ([Martell et al., 2004](#_ENREF_41)) |
| NaClO_4_ | 298.15 | 1.000 | 9.75 | 2.44 | ([Gergely et al., 1974b](#_ENREF_24)) |
| NaNO_3_ | 298.15 | 1.000 | 9.75 | 2.42 | ([Jawaid et al., 1978](#_ENREF_34)) |
| NaClO_4_ | 298.15 | 1.500 | 9.77 | 2.54 | ([Gergely et al., 1974b](#_ENREF_24)) |
| NaClO_4_ | 298.15 | 2.000 | 9.87 | 2.62 | ([Martell et al., 2004](#_ENREF_41)) |
| NaClO_4_ | 298.15 | 2.000 | 9.65 | 2.50 | ([Aziz and Lyle, 1971](#_ENREF_5)) |
| NaClO_4_ | 298.15 | 2.000 | 9.87 | 2.62 | ([Gergely et al., 1974b](#_ENREF_24)) |
| KCl | 298.15 | 3.000 | 10.20 | 2.72 | ([Martell et al., 2004](#_ENREF_41)) |
| NaClO_4_ | 298.15 | 3.000 | 9.72 | 2.81 | ([Pettit, 1984](#_ENREF_50)) |
| NaClO_4_ | 298.15 | 3.000 | 9.72 | 2.81 | ([Matsui and Ohtaki, 1982](#_ENREF_42)) |
|  | 298.15 | 3.000 | 9.72 |  | ([Matsui and Ohtaki, 1982](#_ENREF_42)) |
| NaCl | 298.15 | 5.000 | 10.75 | 3.01 | ([Pettit, 1984](#_ENREF_50)) |
| NaClO_4_ | 298.15 | 5.000 | 10.30 | 3.01 | ([Martell et al., 2004](#_ENREF_41)) |
| -- | 303.15 | 0 | 9.74 | 2.33 | ([Nims and Smith, 1933](#_ENREF_45)) |
|  | 303.15 | 0 | 9.78 | 2.36 | ([Doğan et al., 2002](#_ENREF_17)) |
|  | 303.15 | 0 | 9.78 | 2.28 | ([Gergely et al., 1971](#_ENREF_22)) |
| KCl | 303.15 | 0.050 | 9.60 | 2.28 | ([Gergely et al., 1971](#_ENREF_22)) |
| NaClO_4_ | 303.15 | 0.100 | 9.67 | 2.48 | ([Ramanujam et al., 1979](#_ENREF_53)) |
|  | 303.15 | 0.100 | 9.70 |  | ([Berezina et al., 1973](#_ENREF_6)) |
| NaClO_4_ | 303.15 | 0.200 | 9.76 | 2.31 | ([Pettit, 1984](#_ENREF_50)) |
| NaNO_3_ | 303.15 | 0.200 | 9.61 | 2.35 | ([Ramanujam and Selvarajan, 1981](#_ENREF_54)) |
| KNO_3_ | 303.15 | 0.250 | 9.58 | 2.53 | ([Pettit, 1984](#_ENREF_50)) |
|  | 303.15 | 1.000 | 9.70 |  | ([Chandel and Gupta, 1982](#_ENREF_11)) |
|  | 303.15 | 1.000 | 9.70 |  | ([Chandel and Gupta, 1984](#_ENREF_12)) |
| NaClO_4_ | 304.15 | 0.100 | 9.67 | 2.48 | ([Rangaraj and Ramanujam, 1977](#_ENREF_55)) |
|  | 308.15 | 0 | 10.06 |  | ([Hamborg et al., 2007](#_ENREF_30)) |
|  | 308.15 | 0 | 9.66 | 2.28 | ([Gergely et al., 1971](#_ENREF_22)) |
| KCl | 308.15 | 0.050 | 9.48 | 2.28 | ([Gergely et al., 1971](#_ENREF_22)) |
| KNO_3_ | 308.15 | 0.200 | 9.33 | 2.27 | ([Prasad and Mohan, 1987](#_ENREF_52)) |
| NaClO_4_ | 308.15 | 0.200 |  | 2.45 | ([Sarin and Munshi, 1973](#_ENREF_56)) |
| NaClO_4_ | 310.15 | 0.100 | 9.91 | 2.58 | ([Pettit, 1984](#_ENREF_50)) |
| NaCl | 310.15 | 0.150 | 9.40 | 2.31 | ([Martell et al., 2004](#_ENREF_41)) |
| NaClO_4_ | 310.15 | 0.150 | 9.55 | 2.26 | ([Nair and Santappa, 1981](#_ENREF_44)) |
| NaClO_4_ | 310.15 | 0.150 | 9.40 | 2.37 | ([Berthon et al., 1984](#_ENREF_8)) |
|  | 310.15 | 0.150 | 9.50 |  | ([Childs and Perrin, 1969](#_ENREF_14)) |
|  | 310.65 | 0 | 9.55 | 2.33 | ([Smith et al., 1937](#_ENREF_62)) |
| -- | 313.15 | 0 | 9.49 | 2.32 | ([Nims and Smith, 1933](#_ENREF_45)) |
|  | 313.15 | 0 | 9.94 |  | ([Hamborg et al., 2007](#_ENREF_30)) |
|  | 313.15 | 0 | 9.48 | 2.36 | ([Doğan et al., 2002](#_ENREF_17)) |
|  | 313.15 | 0 | 9.51 | 2.35 | ([Anderson et al., 1966](#_ENREF_2)) |
|  | 313.15 | 0 | 9.51 | 2.65 | ([Anderson et al., 1967](#_ENREF_1)) |
|  | 313.15 | 0.100 | 9.52 |  | ([Berezina et al., 1973](#_ENREF_6)) |
| KCl | 313.15 | 0.200 | 9.41 | 2.44 | ([Sharma and Mathur, 1965](#_ENREF_60)) |
| -- | 318.15 | 0 | 9.38 | 2.32 | ([Nims and Smith, 1933](#_ENREF_45)) |
|  | 318.15 | 0 | 9.82 |  | ([Hamborg et al., 2007](#_ENREF_30)) |
| NaClO_4_ | 318.15 | 0.200 |  | 2.45 | ([Sarin and Munshi, 1973](#_ENREF_56)) |
|  | 323.15 | 0 | 9.70 |  | ([Hamborg et al., 2007](#_ENREF_30)) |
|  | 323.15 | 0 | 9.26 | 2.33 | ([Smith et al., 1937](#_ENREF_62)) |
|  | 323.15 | 0.100 | 9.36 |  | ([Berezina et al., 1973](#_ENREF_6)) |
| KNO_3_ | 323.23 | 0.200 | 9.33 | 2.27 | ([Pettit, 1984](#_ENREF_50)) |
|  | 333.15 | 0.100 | 9.22 |  | ([Berezina et al., 1973](#_ENREF_6)) |
|  | 348.15 | 0 | 9.16 |  | ([Hamborg et al., 2007](#_ENREF_30)) |
|  | 398.15 | 0 | 8.29 |  | ([Hamborg et al., 2007](#_ENREF_30)) |
| Ionic medium | *T* / K | *I* / mol dm^-3^ | Δ$\text{H}_{\text{1}}^{\text{0}}$ / kJ mol^-1^ | Δ$\text{H}_{\text{2}}^{\text{0}}$ / kJ mol^-1^ | Ref. |
|  | 274.15 | 0 | -46.0 | -6.3 | ([Smith et al., 1937](#_ENREF_62)) |
| -- | 278.15 | 0 | -44.7 | -1.5 | ([Nims and Smith, 1933](#_ENREF_45)) |
|  | 283.15 | 0 | -46.0 | -5.2 | ([Christensen et al., 1968](#_ENREF_16)) |
|  | 288.65 | 0 | -46.5 | -5.1 | ([Smith et al., 1937](#_ENREF_62)) |
| -- | 293.15 | 0 | -46.6 | -3.8 | ([Nims and Smith, 1933](#_ENREF_45)) |
| NaCl | 298.15 | 0 | -45.2 | -2.0 | ([Martell et al., 2004](#_ENREF_41)) |
| NaCl | 298.15 | 0 | -45.4 | -2.6 | ([Pettit, 1984](#_ENREF_50)) |
| NaCl | 298.15 | 0 | -41.9 | -0.2 | ([Pettit, 1984](#_ENREF_50)) |
|  | 298.15 | 0 | -46.8 |  | ([Hamborg et al., 2007](#_ENREF_30)) |
| -- | 298.15 | 0 | -45.2 | -3.0 | ([Nims and Smith, 1933](#_ENREF_45)) |
|  | 298.15 | 0 | -46.2 | -3.4 | ([Smith et al., 1937](#_ENREF_62)) |
|  | 298.15 | 0 | -45.3 | -2.6 | ([Sturtevant, 1942](#_ENREF_65)) |
|  | 298.15 | 0 | -44.3 | -2.9 | ([Doğan et al., 2002](#_ENREF_17)) |
|  | 298.15 | 0 | -43.5 | -2.9 | ([Anderson et al., 1966](#_ENREF_2)) |
|  | 298.15 | 0 | -45.0 |  | ([Avedikian, 1967](#_ENREF_4)) |
|  | 298.15 | 0 |  | -3.1 | ([Christensen et al., 1968](#_ENREF_16)) |
| NaCl | 298.15 | 0.100 | -45.0 | -3.1 | ([Pettit, 1984](#_ENREF_50)) |
| KNO_3_ | 298.15 | 0.100 | -45.0 | -3.1 | ([Arena et al., 1983](#_ENREF_3)) |
|  | 298.15 | 0.160 | -44.4 |  | ([Meyer and Bauman, 1970](#_ENREF_43)) |
| KCl | 298.15 | 0.200 | -47.2 | -2.7 | ([Gergely and Sóvágó, 1973](#_ENREF_25)) |
| NaCl | 298.15 | 0.500 | -46.8 | -3.0 | ([Martell et al., 2004](#_ENREF_41)) |
| NaCl | 298.15 | 0.500 | -46.5 | -3.7 | ([Pettit, 1984](#_ENREF_50)) |
| NaCl | 298.15 | 1.000 | -48.1 | -4.1 | ([Martell et al., 2004](#_ENREF_41)) |
|  | 298.15 | 1.000 | -49.2 |  | ([Enea et al., 1979](#_ENREF_18)) |
| -- | 303.15 | 0 | -44.9 | -2.3 | ([Nims and Smith, 1933](#_ENREF_45)) |
| NaCl | 308.15 | 0 | -44.9 | -1.0 | ([Martell et al., 2004](#_ENREF_41)) |
|  | 310.65 | 0 | -45.6 | -1.3 | ([Smith et al., 1937](#_ENREF_62)) |
| -- | 313.15 | 0 | -44.8 | -0.9 | ([Nims and Smith, 1933](#_ENREF_45)) |
|  | 313.15 | 0 |  | -1.0 | ([Christensen et al., 1968](#_ENREF_16)) |
| -- | 318.15 | 0 | -44.3 | -0.3 | ([Nims and Smith, 1933](#_ENREF_45)) |
| NaCl | 318.15 | 0.500 | -45.4 | -1.4 | ([Pettit, 1984](#_ENREF_50)) |
|  | 323.15 | 0 | -44.3 | 1.1 | ([Smith et al., 1937](#_ENREF_62)) |

**Table 6S** Experimental values of the protonation constants of L-leucine in NaCl in the molal concentration scale

| *I* / mol kg^-1^ | *T* / K | log $\text{K}_{\text{1}}^{\text{H}}$ | log $\text{K}_{\text{2}}^{\text{H}}$ |
| --- | --- | --- | --- |
| 0 | 274.15 | 10.472±0.014 ^a^ | 2.384±0.001 ^a^ |
| 0 | 283.15 | 10.186±0.016 | 2.346±0.006 |
| 0 | 285.65 | 10.113±0.014 | 2.339±0.007 |
| 0.102 | 288.15 | 9.867±0.008 | 2.325±0.013 |
| 0.477 | 288.15 | 9.873±0.007 | 2.387±0.006 |
| 0.943 | 288.15 | 9.970±0.004 | 2.467±0.001 |
| 2.598 | 288.15 | 10.375±0.005 | 2.759±0.004 |
| 2.733 | 288.15 | 10.408±0.013 | 2.781±0.002 |
| 4.046 | 288.15 | 10.736±0.012 | 3.080±0.071 |
| 4.401 | 288.15 | 10.875±0.058 | 3.077±0.011 |
| 0 | 293.15 | 9.915±0.004 | 2.325±0.004 |
| 0.010 | 293.15 | 9.866±0.041 | 2.326±0.004 |
| 0.151 | 293.15 | 9.723±0.005 | 2.343±0.003 |
| 0.511 | 293.15 | 9.772±0.037 | 2.393±0.005 |
| 0.010 | 295.15 | 9.706±0.068 | 2.322±0.004 |
| 0 | 298.15 | 9.762±0.014 | 2.315±0.011 |
| 0 | 298.15 | 9.777±0.002 | 2.320±0.016 |
| 0 | 298.15 | 9.841±0.069 | 2.423±0.123 |
| 0 | 298.15 | 9.657±0.123 | 2.363±0.061 |
| 0 | 298.15 | 9.696±0.083 | 2.363±0.061 |
| 0.010 | 298.15 | 9.651±0.041 | 2.329±0.024 |
| 0.010 | 298.15 | 9.724±0.035 | 2.329±0.024 |
| 0.056 | 298.15 | 9.625±0.008 | 2.333±0.028 |
| 0.101 | 298.15 | 9.586±0.007 | 2.292±0.021 |
| 0.101 | 298.15 | 9.643±0.052 | 2.318±0.000 |
| 0.101 | 298.15 | 9.634±0.043 | 2.348±0.032 |
| 0.101 | 298.15 | 9.664±0.074 | 2.335±0.018 |
| 0.101 | 298.15 | 9.621±0.029 | 2.322±0.005 |
| 0.101 | 298.15 | 9.586±0.007 | 2.317±0.000 |
| 0.101 | 298.15 | 9.586±0.007 | 2.326±0.009 |
| 0.101 | 298.15 | 9.607±0.015 | 2.373±0.058 |
| 0.101 | 298.15 | 9.517±0.079 | 2.476±0.165 |
| 0.101 | 298.15 | 9.620±0.029 | 2.300±0.018 |
| 0.101 | 298.15 | 9.625±0.033 | 2.317±0.000 |
| 0.101 | 298.15 | 9.639±0.048 | 2.377±0.062 |
| 0.101 | 298.15 | 9.604±0.011 | 2.303±0.015 |
| 0.101 | 298.15 | 9.556±0.038 | 2.457±0.145 |
| 0.101 | 298.15 | 9.577±0.017 | 2.330±0.013 |
| 0.101 | 298.15 | 9.642±0.051 | 2.328±0.011 |
| 0.101 | 298.15 | 9.445±0.155 | 2.300±0.018 |
| 0.101 | 298.15 | 9.719±0.132 | 2.497±0.188 |
| 0.101 | 298.15 | 9.659±0.069 | 2.279±0.040 |
| 0.101 | 298.15 | 9.630±0.039 | 2.329±0.003 |
| 0.138 | 298.15 | 9.586±0.005 | 2.303±0.020 |
| 0.162 | 298.15 | 9.560±0.019 | 2.337±0.003 |
| 0.202 | 298.15 | 9.656±0.086 | 2.342±0.003 |
| 0.202 | 298.15 | 9.613±0.041 | 2.329±0.003 |
| 0.202 | 298.15 | 9.630±0.059 | 2.324±0.007 |
| 0.305 | 298.15 | 9.560±0.013 | 2.353±0.008 |
| 0.450 | 298.15 | 9.590±0.004 | 2.374±0.007 |
| 0.506 | 298.15 | 9.581±0.013 | 2.329±0.050 |
| 0.512 | 298.15 | 9.576±0.019 | 2.391±0.015 |
| 0.512 | 298.15 | 9.584±0.011 | 2.391±0.015 |
| 0.512 | 298.15 | 9.657±0.065 | 2.524±0.153 |
| 0.723 | 298.15 | 9.602±0.027 | 2.417±0.007 |
| 0.864 | 298.15 | 9.652±0.002 | 2.432±0.001 |
| 1.240 | 298.15 | 9.727±0.002 | 2.497±0.004 |
| 1.763 | 298.15 | 9.842±0.000 | 2.581±0.000 |
| 2.757 | 298.15 | 10.042±0.028 | 2.748±0.003 |
| 5.035 | 298.15 | 10.539±0.070 | 3.089±0.057 |
| 0.101 | 308.15 | 9.227±0.111 | 2.114±0.210 |
| 0.203 | 308.15 | 9.274±0.039 | 2.301±0.029 |
| 0.151 | 310.15 | 9.259±0.011 | 2.320±0.004 |
| 0.152 | 310.15 | 9.306±0.038 | 2.337±0.013 |
| 0.152 | 310.15 | 9.265±0.005 | 2.321±0.003 |
| 0 | 310.65 | 9.448±0.011 | 2.315±0.010 |
| 0 | 313.15 | 9.389±0.012 | 2.313±0.005 |
| 0.101 | 318.15 | 8.786±0.322 | 1.817±0.535 |
| 0.104 | 318.15 | 9.100±0.008 | 2.335±0.004 |
| 0.489 | 318.15 | 9.063±0.018 | 2.354±0.030 |
| 0.957 | 318.15 | 9.160±0.023 | 2.455±0.004 |
| 2.781 | 318.15 | 9.469±0.014 | 2.710±0.032 |
| 4.512 | 318.15 | 9.860±0.034 | 3.034±0.013 |
| 0 | 323.15 | 9.161±0.015 | 2.332±0.001 |

^a^ ± 95 % C.I.

**Table 7S** Experimental values of the protonation constants of L-leucine in (CH_3_)_4_NCl in the molal concentration scale

| *I* / mol kg^-1^ | *T* / K | log $\text{K}_{\text{1}}^{\text{H}}$ | log $\text{K}_{\text{2}}^{\text{H}}$ |
| --- | --- | --- | --- |
| 0.101 | 288.15 | 9.869±0.006 ^a^ | 2.338±0.012 ^a^ |
| 1.034 | 288.15 | 9.837±0.011 | 2.418±0.020 |
| 3.917 | 288.15 | 9.948±0.007 | 2.727±0.005 |
| 0.101 | 298.15 | 9.584±0.001 | 2.306±0.001 |
| 0.523 | 298.15 | 9.531±0.004 | 2.303±0.025 |
| 1.038 | 298.15 | 9.549±0.006 | 2.383±0.015 |
| 1.086 | 298.15 | 9.541±0.005 | 2.353±0.021 |
| 2.141 | 298.15 | 9.578±0.004 | 2.475±0.002 |
| 3.459 | 298.15 | 9.621±0.008 | 2.622±0.003 |
| 3.973 | 298.15 | 9.650±0.003 | 2.675±0.001 |
| 0.102 | 318.15 | 9.086±0.005 | 2.302±0.016 |
| 1.050 | 318.15 | 9.039±0.007 | 2.362±0.001 |
| 4.025 | 318.15 | 9.126±0.003 | 2.620±0.004 |

^a^ ± 95 % C.I.

**Table 8S** Experimental values of the protonation constants of L-valine in NaCl in the molal concentration scale

| *I* / mol kg^-1^ | *T* / K | log $\text{K}_{\text{1}}^{\text{H}}$ | log $\text{K}_{\text{2}}^{\text{H}}$ |
| --- | --- | --- | --- |
| 0 | 274.15 | 10.431±0.015 ^a^ | 2.336±0.014 ^a^ |
| 0 | 283.15 | 10.151±0.015 | 2.306±0.005 |
| 0 | 283.15 | 10.114±0.062 | 2.318±0.010 |
| 0 | 285.65 | 10.079±0.013 | 2.300±0.003 |
| 0 | 288.15 | 9.970±0.058 | 2.310±0.017 |
| 0 | 293.15 | 9.762±0.143 | 2.296±0.003 |
| 0.100 | 293.15 | 9.671±0.018 | 2.273±0.028 |
| 0.100 | 293.15 | 9.671±0.019 | 2.273±0.029 |
| 0.151 | 293.15 | 9.656±0.015 | 2.003±0.371 |
| 0.151 | 293.15 | 9.656±0.015 | 2.302±0.004 |
| 0.511 | 293.15 | 9.711±0.066 | 2.489±0.201 |
| 0.101 | 295.15 | 9.613±0.021 | 2.255±0.049 |
| 0.512 | 296.15 | 9.588±0.018 | 2.333±0.007 |
| 0 | 298.15 | 9.717±0.011 | 2.292±0.018 |
| 0.010 | 298.15 | 9.642±0.018 | 2.299±0.017 |
| 0.010 | 298.15 | 9.682±0.032 | 2.251±0.043 |
| 0.020 | 298.15 | 9.701±0.091 | 2.288±0.002 |
| 0.100 | 298.15 | 9.613±0.080 | 2.367±0.093 |
| 0.100 | 298.15 | 9.536±0.015 | 2.283±0.012 |
| 0.101 | 298.15 | 9.512±0.046 | 2.295±0.002 |
| 0.101 | 298.15 | 9.559±0.026 | 2.332±0.057 |
| 0.101 | 298.15 | 9.576±0.035 | 2.403±0.137 |
| 0.101 | 298.15 | 9.584±0.045 | 2.259±0.043 |
| 0.101 | 298.15 | 9.612±0.081 | 2.367±0.093 |
| 0.101 | 298.15 | 9.544±0.005 | 2.287±0.007 |
| 0.101 | 298.15 | 9.547±0.001 | 2.296±0.004 |
| 0.101 | 298.15 | 9.539±0.011 | 2.272±0.026 |
| 0.111 | 298.15 | 9.513±0.038 | 2.275±0.023 |
| 0.151 | 298.15 | 9.546±0.000 | 2.298±0.004 |
| 0.162 | 298.15 | 9.490±0.045 | 2.298±0.004 |
| 0.202 | 298.15 | 9.549±0.038 | 2.303±0.001 |
| 0.252 | 298.15 | 9.495±0.023 | 2.298±0.009 |
| 0.506 | 298.15 | 9.488±0.036 | 2.315±0.016 |
| 0.509 | 298.15 | 9.591±0.092 | 2.322±0.008 |
| 0.512 | 298.15 | 9.517±0.006 | 2.317±0.014 |
| 0.513 | 298.15 | 9.554±0.046 | 2.333±0.005 |
| 1.035 | 298.15 | 9.573±0.020 | 2.406±0.070 |
| 0 | 303.15 | 9.564±0.062 | 2.299±0.018 |
| 0.101 | 303.15 | 9.444±0.035 | 2.296±0.003 |
| 0.151 | 303.15 | 9.421±0.030 | 2.299±0.004 |
| 0.513 | 303.15 | 9.417±0.044 | 2.331±0.017 |
| 0 | 308.15 | 9.446±0.055 | 2.302±0.015 |
| 0.101 | 308.15 | 9.260±0.037 | 2.301±0.003 |
| 0.203 | 308.15 | 9.260±0.005 | 2.284±0.034 |
| 0.203 | 308.15 | 9.260±0.005 | 2.284±0.034 |
| 0.151 | 310.15 | 9.388±0.210 | 2.307±0.004 |
| 0.151 | 310.15 | 9.198±0.027 | 2.292±0.022 |
| 0.152 | 310.15 | 9.257±0.047 | 2.319±0.013 |
| 0.152 | 310.15 | 9.253±0.042 | 2.279±0.038 |
| 0.152 | 310.15 | 9.253±0.042 | 2.279±0.038 |
| 0.152 | 310.15 | 9.234±0.019 | 2.338±0.037 |
| 0.152 | 310.15 | 9.235±0.019 | 2.340±0.039 |
| 0.152 | 310.15 | 9.229±0.013 | 2.303±0.008 |
| 0 | 310.65 | 9.420±0.013 | 2.293±0.001 |
| 0 | 313.15 | 9.361±0.013 | 2.297±0.003 |
| 0 | 313.15 | 9.323±0.061 | 2.307±0.011 |
| 0.152 | 313.15 | 9.148±0.006 | 2.313±0.004 |
| 0 | 323.15 | 9.139±0.013 | 2.321±0.009 |

^a^ ± 95 % C.I.

**Table 9S** Experimental values of the protonation constants of L-valine in (CH_3_)_4_NCl in the molal concentration scale at *T* = 298.15 K

| *I* / mol kg^-1^ | log $\text{K}_{\text{1}}^{\text{H}}$ | log $\text{K}_{\text{2}}^{\text{H}}$ |
| --- | --- | --- |
| 0.524 | 9.449±0.024 ^a^ | 2.263±0.049 ^a^ |
| 1.148 | 9.463±0.002 | 2.352±0.003 |
| 2.368 | 9.496±0.009 | 2.485±0.021 |
| 4.421 | 9.558±0.002 | 2.686±0.004 |

^a^ ± 95 % C.I.

**Table 10S** Experimental values of the protonation constants of L-serine in NaCl in the molal concentration scale

| *I* / mol kg^-1^ | *T* / K | log $\text{K}_{\text{1}}^{\text{H}}$ | log $\text{K}_{\text{2}}^{\text{H}}$ |
| --- | --- | --- | --- |
| 0 | 274.15 | 9.910±0.023 ^a^ | 2.294±0.008 ^a^ |
| 0.050 | 283.15 | 9.534±0.028 | 2.246±0.019 |
| 0 | 285.15 | 9.580±0.030 | 2.228±0.005 |
| 0 | 285.65 | 9.572±0.023 | 2.227±0.005 |
| 0.202 | 288.15 | 9.314±0.017 | 2.237±0.047 |
| 0.050 | 292.65 | 9.290±0.047 | 2.206±0.018 |
| 0 | 293.15 | 9.369±0.023 | 2.188±0.001 |
| 0.010 | 293.15 | 9.277±0.029 | 2.195±0.004 |
| 0.050 | 293.15 | 9.166±0.068 | 2.179±0.008 |
| 0.100 | 293.15 | 9.110±0.088 | 2.183±0.001 |
| 0.101 | 293.15 | 9.187±0.007 | 2.183±0.001 |
| 0.151 | 293.15 | 9.176±0.002 | 1.874±0.318 |
| 0.511 | 293.15 | 9.193±0.045 | 2.438±0.276 |
| 0.723 | 293.15 | 9.081±0.090 | 2.191±0.006 |
| 0.100 | 295.15 | 9.170±0.030 | 2.177±0.001 |
| 0 | 298.15 | 9.225±0.042 | 2.184±0.008 |
| 0.050 | 298.15 | 9.122±0.020 | 2.188±0.016 |
| 0.050 | 298.15 | 9.079±0.024 | 2.166±0.007 |
| 0.050 | 298.15 | 9.101±0.002 | 2.162±0.011 |
| 0.050 | 298.15 | 9.092±0.011 | 2.171±0.002 |
| 0.060 | 298.15 | 9.113±0.021 | 2.171±0.001 |
| 0.097 | 298.15 | 9.065±0.001 | 2.171±0.002 |
| 0.097 | 298.15 | 9.068±0.002 | 2.147±0.024 |
| 0.100 | 298.15 | 9.123±0.061 | 2.245±0.079 |
| 0.101 | 298.15 | 9.057±0.007 | 2.165±0.005 |
| 0.101 | 298.15 | 9.104±0.044 | 2.156±0.083 |
| 0.101 | 298.15 | 9.070±0.025 | 2.192±0.029 |
| 0.151 | 298.15 | 9.003±0.043 | 2.185±0.018 |
| 0.151 | 298.15 | 9.032±0.012 | 2146±0.023 |
| 0.151 | 298.15 | 9.004±0.041 | 2.184±0.018 |
| 0.151 | 298.15 | 9.083±0.041 | 2.170±0.002 |
| 0.152 | 298.15 | 9.049±0.003 | 2.150±0.021 |
| 0.161 | 298.15 | 9.099±0.061 | 2.170±0.002 |
| 0.202 | 298.15 | 9.067±0.041 | 2.196±0.049 |
| 0.202 | 298.15 | 9.032±0.002 | 2.181±0.020 |
| 0.202 | 298.15 | 9.067±0.038 | 2.217±0.053 |
| 0.202 | 298.15 | 9.054±0.024 | 2.161±0.005 |
| 0.251 | 298.15 | 9.024±0.002 | 2.399±0.244 |
| 0.252 | 298.15 | 9.024±0.002 | 2.166±0.001 |
| 0.475 | 298.15 | 9.015±0.000 | 2.183±0.015 |
| 0.475 | 298.15 | 9.009±0.007 | 2.168±0.001 |
| 0.506 | 298.15 | 9.007±0.010 | 2.181±0.011 |
| 0.512 | 298.15 | 9.031±0.015 | 2.180±0.004 |
| 0.918 | 298.15 | 9.052±0.003 | 2.209±0.015 |
| 0.918 | 298.15 | 9.055±0.000 | 2.210±0.016 |
| 1.045 | 298.15 | 9.084±0.013 | 2.219±0.014 |
| 1.548 | 298.15 | 9.159±0.009 | 2.269±0.016 |
| 1.918 | 298.15 | 9.215±0.001 | 2.289±0.006 |
| 2.086 | 298.15 | 9.230±0.018 | 2.311±0.003 |
| 2.635 | 298.15 | 9.351±0.001 | 2.380±0.000 |
| 2.972 | 298.15 | 9.391±0.028 | 2.389±0.034 |
| 2.972 | 298.15 | 9.402±0.016 | 2.372±0.052 |
| 3.198 | 298.15 | 9.466±0.003 | 2.438±0.014 |
| 3.199 | 298.15 | 9.467±0.004 | 2.439±0.013 |
| 3.199 | 298.15 | 9.457±0.006 | 2.446±0.005 |
| 3.199 | 298.15 | 9.470±0.008 | 2.438±0.014 |
| 3.199 | 298.15 | 9.467±0.004 | 2.439±0.013 |
| 3.896 | 298.15 | 9.627±0.023 | 2.548±0.007 |
| 3.924 | 298.15 | 9.624±0.014 | 2.547±0.002 |
| 4.300 | 298.15 | 9.720±0.033 | 2.652±0.060 |
| 4.369 | 298.15 | 9.685±0.019 | 2.572±0.033 |
| 4.462 | 298.15 | 9.738±0.016 | 2.651±0.036 |
| 5.606 | 298.15 | 9.944±0.020 | 2.770±0.003 |
| 0.050 | 303.15 | 8.996±0.017 | 2.174±0.011 |
| 0.101 | 303.15 | 8.943±0.003 | 2.162±0.001 |
| 0.202 | 303.15 | 8.922±0.018 | 2.164±0.002 |
| 0.254 | 303.15 | 8.857±0.041 | 2.162±0.004 |
| 0.513 | 303.15 | 9.009±0.125 | 2.182±0.016 |
| 1.047 | 303.15 | 9.004±0.067 | 2.233±0.006 |
| 0.203 | 308.15 | 8.746±0.041 | 2.173±0.018 |
| 0 | 310.15 | 8.946±0.033 | 2.156±0.004 |
| 0.151 | 310.15 | 8.737±0.017 | 2.110±0.047 |
| 0.151 | 310.15 | 8.738±0.016 | 2.136±0.021 |
| 0.151 | 310.15 | 8.866±0.118 | 2.139±0.018 |
| 0.151 | 310.15 | 8.737±0.017 | 2.126±0.031 |
| 0.151 | 310.15 | 8.732±0.022 | 2.133±0.024 |
| 0.151 | 310.15 | 8.740±0.013 | 2.156±0.004 |
| 0.152 | 310.15 | 8.757±0.026 | 2.146±.017 |
| 0 | 310.65 | 8.940±0.028 | 2.156±0.004 |
| 0.050 | 313.15 | 8.759±0.013 | 2.160±0.005 |
| 0.101 | 313.15 | 8.736±0.031 | 2.158±0.002 |
| 0.203 | 313.15 | 8.714±0.046 | 2.203±0.048 |
| 0 | 323.15 | 8.669±0.032 | 2.149±0.015 |
| 0.102 | 323.15 | 8.522±0.033 | 2.158±0.002 |
| 0.202 | 328.15 | 8.354±0.004 | 2.209±0.033 |
| 0.102 | 333.15 | 8.326±0.036 | 2.196±0.003 |

^a^ ± 95 % C.I.

**Table 11S** Experimental values of the protonation constants of L-serine in (CH_3_)_4_NCl in the molal concentration scale at *T* = 298.15 K

| *I* / mol kg^-1^ | log $\text{K}_{\text{1}}^{\text{H}}$ | log $\text{K}_{\text{2}}^{\text{H}}$ |
| --- | --- | --- |
| 0.105 | 9.073±0.004 ^a^ | 2.173±0.019 ^a^ |
| 0.492 | 9.023±0.004 | 2.251±0.011 |
| 1.028 | 9.019±0.004 | 2.293±0.006 |
| 2.311 | 9.058±0.000 | 2.430±0.001 |
| 4.041 | 9.112±0.000 | 2.596±0.000 |

^a^ ± 95 % C.I.

**Table 12S** Experimental values of the protonation constants of L-phenylalanine in NaCl in the molal concentration scale

| *I* / mol kg^-1^ | *T* / K | log $\text{K}_{\text{1}}^{\text{H}}$ | log $\text{K}_{\text{2}}^{\text{H}}$ |
| --- | --- | --- | --- |
| 0 | 273.15 | 9.974±0.007 ^a^ | 2.234±0.020 ^a^ |
| 0.100 | 278.15 | 9.689±0.006 | 2.210±0.008 |
| 0 | 283.15 | 9.671±0.006 | 2.176±0.020 |
| 0 | 283.15 | 9.710±0.006 | 2.146±0.020 |
| 0 | 293.15 | 9.388±0.006 | 2.156±0.020 |
| 0.005 | 293.15 | 9.330±0.006 | 2.133±0.011 |
| 0.010 | 293.15 | 9.309±0.006 | 2.090±0.019 |
| 0.050 | 293.15 | 9.219±0.005 | 2.119±0.016 |
| 0.101 | 293.15 | 9.066±0.005 | 2.257±0.014 |
| 0.373 | 293.15 | 9.234±0.013 | 2.228±0.013 |
| 0.100 | 296.15 | 9.163±0.005 | 2.177±0.014 |
| 0.101 | 297.15 | 9.100±0.005 | 2.155±0.014 |
| 0 | 298.15 | 9.281±0.006 | 2.152±0.020 |
| 0.050 | 298.15 | 9.090±0.005 | 2.114±0.017 |
| 0.050 | 298.15 | 9.103±0.005 | 2.161±0.017 |
| 0.100 | 298.15 | 9.061±0.005 | 2.157±0.014 |
| 0.101 | 298.15 | 9.046±0.005 | 2.158±0.014 |
| 0.101 | 298.15 | 9.095±0.005 | 2.154±0.014 |
| 0.151 | 298.15 | 9.090±0.007 | 2.095±0.013 |
| 0.162 | 298.15 | 9.027±0.007 | 2.168±0.007 |
| 0.202 | 298.15 | 9.068±0.008 | 2.104±0.013 |
| 0.481 | 298.15 | 8.983±0.015 | 2.255±0.018 |
| 0.506 | 298.15 | 9.021±0.015 | 2.267±0.018 |
| 0.512 | 298.15 | 9.028±0.015 | 2.262±0.018 |
| 0.512 | 298.15 | 9.024±0.015 | 2.284±0.018 |
| 0.723 | 298.15 | 9.027±0.017 | 2.151±0.022 |
| 0.966 | 298.15 | 9.053±0.019 | 2.377±0.023 |
| 1.020 | 298.15 | 9.101±0.019 | 2.328±0.024 |
| 1.948 | 298.15 | 9.204±0.033 | 2.532±0.020 |
| 2.976 | 298.15 | 9.433±0.066 | 2.693±0.020 |
| 0 | 303.15 | 9.139±0.006 | 2.163±0.020 |
| 0.050 | 303.15 | 8.968±0.005 | 2.112±0.017 |
| 0.101 | 303.15 | 8.971±0.005 | 2.241±0.014 |
| 0.203 | 303.15 | 8.929±0.008 | 2.575±0.013 |
| 0.203 | 303.15 | 9.027±0.008 | 2.065±0.013 |
| 0.050 | 308.15 | 8.846±0.005 | 2.113±0.017 |
| 0.101 | 308.15 | 8.840±0.005 | 1.907±0.014 |
| 0.203 | 308.15 | 9.003±0.008 | 2.220±0.013 |
| 0.203 | 308.15 | 8.750±0.008 | 2.151±0.013 |
| 0.151 | 310.15 | 8.743±0.007 | 2.157±0.013 |
| 0.151 | 310.15 | 8.749±0.007 | 2.161±0.013 |
| 0 | 313.15 | 8.890±0.006 | 2.154±0.020 |
| 0 | 313.15 | 8.920±0.006 | 2.159±0.020 |
| 0.101 | 313.15 | 8.732±0.005 | 2.227±0.014 |
| 0.101 | 318.15 | 8.578±0.005 | 1.755±0.015 |
| 0.102 | 323.15 | 8.503±0.006 | 2.229±0.015 |
| 0.102 | 333.15 | 8.332±0.007 | 2.282±0.017 |

^a^ ± 95 % C.I.

**Table 13S** Experimental values of the protonation constants of L-phenylalanine in (CH_3_)_4_NCl in the molal concentration scale at *T* = 298.15 K

| *I* / mol kg^-1^ | log $\text{K}_{\text{1}}^{\text{H}}$ | log $\text{K}_{\text{2}}^{\text{H}}$ |
| --- | --- | --- |
| 0.070 | 9.051±0.007 | 2.124±0.017 |
| 0.613 | 8.958±0.023 | 2.232±0.012 |
| 1.299 | 8.950±0.027 | 2.330±0.012 |
| 3.045 | 8.988±0.014 | 2.546±0.019 |
| 3.937 | 9.023±0.011 | 2.655±0.029 |

^a^ ± 95 % C.I.

**Table 14S** Experimental values of the protonation constants of L-Alanine in NaCl in the molal concentration scale

| *I* / mol kg^-1^ | *T* / K | log $\text{K}_{\text{1}}^{\text{H}}$ | log $\text{K}_{\text{2}}^{\text{H}}$ |
| --- | --- | --- | --- |
| 0.100 | 273.15 | 10.346±0.012 ^a^ | 2.589±0.037 ^a^ |
| 0 | 283.15 | 10.313±0.016 | 2.399±0.044 |
| 0.100 | 293.15 | 9.831±0.007 | 2.425±0.034 |
| 0.511 | 293.15 | 9.883±0.025 | 2.630±0.028 |
| 1.048 | 293.15 | 9.869±0.036 | 2.479±0.037 |
| 0 | 298.15 | 9.891±0.014 | 2.356±0.044 |
| 0 | 298.15 | 9.886±0.014 | 2.353±0.044 |
| 0.090 | 298.15 | 9.728±0.007 | 2.351±0.035 |
| 0.090 | 298.15 | 9.735±0.007 | 2.355±0.035 |
| 0.101 | 298.15 | 9.719±0.007 | 2.355±0.034 |
| 0.101 | 298.15 | 9.753±0.007 | 2.496±0.034 |
| 0.101 | 298.15 | 9.676±0.007 | 2.431±0.034 |
| 0.101 | 298.15 | 9.729±0.007 | 2.359±0.034 |
| 0.101 | 298.15 | 9.745±0.007 | 2.371±0.034 |
| 0.151 | 298.15 | 9.687±0.008 | 2.374±0.030 |
| 0.202 | 298.15 | 9.694±0.010 | 2.368±0.028 |
| 0.202 | 298.15 | 9.686±0.010 | 2.260±0.028 |
| 0.305 | 298.15 | 9.697±0.016 | 2.394±0.026 |
| 0.486 | 298.15 | 9.774±0.024 | 2.408±0.028 |
| 0.486 | 298.15 | 9.746±0.024 | 2.422±0.028 |
| 0.506 | 298.15 | 9.697±0.025 | 2.385±0.028 |
| 0.608 | 298.15 | 9.708±0.028 | 2.403±0.030 |
| 0.918 | 298.15 | 9.770±0.034 | 2.442±0.035 |
| 0.918 | 298.15 | 9.773±0.034 | 2.470±0.035 |
| 1.022 | 298.15 | 9.754±0.035 | 2.436±0.036 |
| 1.035 | 298.15 | 9.768±0.035 | 2.438±0.036 |
| 1.045 | 298.15 | 9.801±0.035 | 2.454±0.037 |
| 1.895 | 298.15 | 9.946±0.037 | 2.562±0.037 |
| 2.206 | 298.15 | 9.919±0.038 | 2.590±0.036 |
| 2.972 | 298.15 | 10.136±0.049 | 2.659±0.033 |
| 2.972 | 298.15 | 10.138±0.049 | 2.698±0.033 |
| 3.307 | 298.15 | 10.170±0.056 | 2.704±0.035 |
| 3.490 | 298.15 | 9.972±0.061 | 2.745±0.036 |
| 3.795 | 298.15 | 10.301±0.069 | 2.807±0.039 |
| 3.970 | 298.15 | 10.342±0.075 | 2.822±0.042 |
| 4.315 | 298.15 | 10.462±0.086 | 2.929±0.048 |
| 4.516 | 298.15 | 10.487±0.092 | 2.946±0.052 |
| 5.606 | 298.15 | 10.633±0.131 | 2.975±0.079 |
| 6.557 | 298.15 | 10.508±0.167 | 3.008±0.105 |
| 0.203 | 303.15 | 9.653±0.010 | 2.346±0.028 |
| 0.254 | 303.15 | 9.574±0.013 | 2.443±0.027 |
| 0.101 | 310.15 | 9.631±0.008 | 2.455±0.034 |
| 0.151 | 310.15 | 9.404±0.009 | 2.342±0.030 |
| 0.000 | 313.15 | 9.519±0.015 | 2.342±0.044 |
| 0.203 | 313.15 | 9.362±0.011 | 2.399±0.028 |
| 0.204 | 323.23 | 9.196±0.013 | 2.330±0.028 |

^a^ ± 95 % C.I.

**Table 15S** Experimental values of the protonation constants of L-Alanine in (CH_3_)_4_NCl in the molal concentration scale at *T* = 298.15 K

| *I* / mol kg^-1^ | log $\text{K}_{\text{1}}^{\text{H}}$ | log $\text{K}_{\text{2}}^{\text{H}}$ |
| --- | --- | --- |
| 0.106 | 9.731±0.029 ^a^ | 2.406±0.038 ^a^ |
| 0.486 | 9.673±0.080 | 2.412±0.054 |
| 1.017 | 9.679±0.100 | 2.467±0.063 |
| 1.890 | 9.601±0.088 | 2.464±0.047 |
| 2.331 | 9.686±0.079 | 2.540±0.041 |
| 3.931 | 9.728±0.122 | 2.667±0.102 |

^a^ ± 95 % C.I.

**Table 16S** Experimental values of the protonation constants of L-glycine in NaCl in the molal concentration scale

| *I* / mol kg^-1^ | *T* / K | log $\text{K}_{\text{1}}^{\text{H}}$ | log $\text{K}_{\text{2}}^{\text{H}}$ |
| --- | --- | --- | --- |
| 0 | 278.15 | 10.253±0.02 ^a^ | 2.317±0.03 ^a^ |
| 0 | 283.15 | 10.107±0.02 | 2.308±0.03 |
| 0 | 288.15 | 9.960±0.02 | 2.287±0.03 |
| 0 | 298.15 | 9.684±0.02 | 2.254±0.03 |
| 0.125 | 298.15 | 9.484±0.02 | 2.246±0.02 |
| 0.607 | 298.15 | 9.462±0.04 | 2.304±0.01 |
| 0.840 | 298.15 | 9.507±0.05 | 2.321±0.02 |
| 1.182 | 298.15 | 9.574±0.05 | 2.363±0.02 |
| 2.302 | 298.15 | 9.747±0.04 | 2.479±0.01 |
| 3.438 | 298.15 | 10.008±0.05 | 2.609±0.01 |
| 0 | 308.15 | 9.430±0.02 | 2.229±0.03 |
| 0.189 | 310.15 | 9.157±0.02 | 2.231±0.02 |
| 0 | 313.15 | 9.308±0.02 | 2.216±0.03 |
| 0 | 323.15 | 9.080±0.02 | 2.190±0.03 |
| 0 | 348.15 | 8.569±0.02 | 2.130±0.03 |

^a^ ± 95 % C.I.

**Table 17S** Experimental values of the protonation constants of L-glycine in (CH_3_)_4_NCl in the molal concentration scale at *T* = 298.15 K

| *I* / mol kg^-1^ | log $\text{K}_{\text{1}}^{\text{H}}$ | log $\text{K}_{\text{2}}^{\text{H}}$ |
| --- | --- | --- |
| 0.358 | 9.460±0.03 ^a^ | 2.263±0.04 ^a^ |
| 0.882 | 9.421±0.04 | 2.253±0.06 |
| 1.900 | 9.474±0.05 | 2.413±0.06 |
| 3.069 | 9.497±0.03 | 2.504±0.04 |
| 5.037 | 9.559±0.01 | 2.704±0.02 |

^a^ ± 95 % C.I.

**Table 18S** Experimental values of the protonation enthalpy of L-glycine in NaCl in the molal concentration scale

| *I* / mol kg^-1^ | *T* / K | Δ$\text{H}_{\text{1}}^{\text{0}}$ / kJ mol^-1^ | Δ$\text{H}_{\text{2}}^{\text{0}}$ / kJ mol^-1^ |
| --- | --- | --- | --- |
| 0 | 278.15 | -44.82±0.1 ^a^ | -4.04±0.1 ^a^ |
| 0 | 283.15 | -44.82±0.1 ^a^ | -5.27±0.1 |
| 0 | 288.15 | -44.54±0.2 | -4.04±0.1 |
| 0.505 | 293.15 | -47.75±0.3 | -4.47±0.1 |
| 0.000 | 298.15 | -44.30±0.1 | -4.01±0.1 |
| 0.101 | 298.15 | -44.65±0.1 | -4.16±0.1 |
| 0.303 | 298.15 | -45.44±0.2 | -4.30±0.1 |
| 1.020 | 298.15 | -46.37±0.6 | -4.70±0.1 |
| 1.280 | 298.15 | -47.92±0.7 | -5.06±0.1 |
| 3.200 | 298.15 | -52.95±2.0 | -6.81±0.2 |
| 0 | 308.15 | -43.92±0.1 | -4.04±0.1 |
| 0 | 313.15 | -43.71±0.1 | -2.61±0.1 |
| 0 | 348.15 | -42.98±0.1 | -4.04±0.1 |

^a^ ± 95 % C.I.

**Table 19S** Experimental values of the protonation enthalpy of L-valine in NaCl in the molal concentration scale

| *I* / mol kg^-1^ | *T* / K | Δ$\text{H}_{\text{1}}^{\text{0}}$ / kJ mol^-1^ | Δ$\text{H}_{\text{2}}^{\text{0}}$ / kJ mol^-1^ |
| --- | --- | --- | --- |
| 0 | 274.15 | -44.78±1.16 ^a^ | -3.94±0.20 ^a^ |
| 0 | 283.15 | -45.14±0.04 | -2.65±0.15 |
| 0 | 285.65 | -45.22±0.09 | -2.33±0.11 |
| 0 | 298.15 | -44.97±0.11 | -0.52±0.14 |
| 0.101 | 298.15 | -45.46±0.37 | -0.01±0.50 |
| 0.162 | 298.15 | -45.69±0.27 | -0.50±0.07 |
| 1.022 | 298.15 | -47.43±0.02 | -1.22±0.07 |
| 1.050 | 298.15 | -47.47±0.03 | -1.24±0.08 |
| 0 | 310.65 | -44.22±0.09 | 1.58±0.32 |
| 0 | 313.15 | -44.05±0.15 | 1.89±0.11 |
| 0 | 323.15 | -43.37±0.48 | 3.77±0.71 |

^a^ ± 95 % C.I.

**Table 20S** Experimental values of the protonation enthalpy of L-serine in NaCl in the molal concentration scale

| *I* / mol kg^-1^ | *T* / K | Δ$\text{H}_{\text{1}}^{\text{0}}$ / kJ mol^-1^ | Δ$\text{H}_{\text{2}}^{\text{0}}$ / kJ mol^-1^ |
| --- | --- | --- | --- |
| 0 | 274.15 | -43.65±0.10 ^a^ | -7.90±0.61 ^a^ |
| 0 | 285.65 | -43.46±0.35 | -6.56±0.72 |
| 0 | 293.15 | -42.26±0.60 | -4.89±0.72 |
| 0 | 298.15 | -42.51±0.79 | -3.72±1.24 |
| 0.050 | 298.15 | -42.79±0.11 | -4.47±0.45 |
| 0.101 | 298.15 | -43.17±0.33 | -3.39±0.52 |
| 0.161 | 298.15 | -42.71±0.52 | -3.14±0.18 |
| 0.202 | 298.15 | -43.44±0.16 | -2.49±1.19 |
| 0 | 310.65 | -42.35±0.28 | -3.24±0.73 |
| 0 | 323.15 | -41.41±0.21 | -1.36±0.58 |

^a^ ± 95 % C.I.

**Table 21S** Experimental values of the protonation enthalpy of L-leucine in NaCl in the molal concentration scale

| *I* / mol kg^-1^ | *T* / K | Δ$\text{H}_{\text{1}}^{\text{0}}$ / kJ mol^-1^ | Δ$\text{H}_{\text{2}}^{\text{0}}$ / kJ mol^-1^ |
| --- | --- | --- | --- |
| 0 | 274.15 | -45.96±0.39 ^a^ | -5.10±0.13 ^a^ |
| 0 | 283.15 | -45.97±0.13 | -3.52±0.40 |
| 0 | 285.65 | -45.81±0.14 | -3.56±0.03 |
| 0.010 | 293.15 | -45.44±0.20 | -2.46±0.06 |
| 0 | 298.15 | -45.67±0.43 | -1.81±0.17 |
| 0.100 | 298.15 | -45.88±0.14 | -1.45±0.35 |
| 0.101 | 298.15 | -45.41±0.42 | -2.53±0.06 |
| 0.161 | 298.15 | -45.54±0.52 | -2.58±0.06 |
| 0.500 | 298.15 | -47.07±0.23 | -2.08±0.06 |
| 1.000 | 298.15 | -48.83±0.26 | -2.75±0.20 |
| 2.000 | 298.15 | -51.90±0.17 | -3.23±0.18 |
| 3.000 | 298.15 | -54.96±0.06 | -4.15±0.11 |
| 4.000 | 298.15 | -58.01±0.07 | -5.06±0.05 |
| 5.000 | 298.15 | -61.06±0.19 | -5.97±0.02 |
| 0 | 310.65 | -44.85±0.18 | 0.23±0.11 |
| 0 | 313.15 | -44.70±0.18 | 0.62±0.14 |
| 0 | 323.15 | -43.98±0.39 | 2.37±0.44 |

^a^ ± 95 % C.I.

**Table 22S** Experimental values of the protonation enthalpy of L-leucine in (CH_3_)_4_NCl in the molal concentration scale at *T* = 298.15 K

| *I* / mol kg^-1^ | Δ$\text{H}_{\text{1}}^{\text{0}}$ / kJ mol^-1^ | Δ$\text{H}_{\text{2}}^{\text{0}}$ / kJ mol^-1^ |
| --- | --- | --- |
| 0.100 | -45.65±0.15 | -2.28±0.41 |
| 0.500 | -46.01±0.23 | -2.90±0.31 |
| 1.000 | -46.28±0.17 | -3.72±0.22 |
| 2.000 | -46.79±0.02 | -5.30±0.01 |
| 3.000 | -47.20±0.26 | -7.05±0.04 |

^a^ ± 95 % C.I.

**Table 23S** Experimental values of the protonation enthalpy of L-phenylalanine in NaCl in the molal concentration scale

| *I* / mol kg^-1^ | *T* / K | Δ$\text{H}_{\text{1}}^{\text{0}}$ / kJ mol^-1^ | Δ$\text{H}_{\text{2}}^{\text{0}}$ / kJ mol^-1^ |
| --- | --- | --- | --- |
| 0.100 | 278.15 | -43.09±0.16 ^a^ | -4.58±0.26 ^a^ |
| 0. | 283.15 | -46.31±0.14 | -3.67±0.23 |
| 0.010 | 293.15 | -43.82±0.09 | -2.30±0.18 |
| 0. | 298.15 | -44.88±0.07 | -1.50±0.17 |
| 0.101 | 298.15 | -45.22±0.07 | -1.92±0.17 |
| 0.159 | 298.15 | -44.30±0.07 | -1.93±0.16 |
| 1.022 | 298.15 | -46.48±0.06 | -3.44±0.16 |
| 3.490 | 298.15 | -50.38±0.16 | -9.22±0.36 |
| 0 | 313.15 | -44.11±0.11 | 0.49±0.23 |
| 0.101 | 318.15 | -48.28±0.14 | 0.96±0.26 |

^a^ ± 95 % C.I.

**Table 24S** Experimental values of the protonation enthalpy of L-Alanine in NaCl in the molal concentration scale

| *I* / mol kg^-1^ | *T* / K | Δ$\text{H}_{\text{1}}^{\text{0}}$ / kJ mol^-1^ | Δ$\text{H}_{\text{2}}^{\text{0}}$ / kJ mol^-1^ |
| --- | --- | --- | --- |
| 0 | 283.15 | -45.41±0.71 ^a^ | -4.58±0.78 ^a^ |
| 0 | 298.15 | -44.72±0.71 | -2.37±0.47 |
| 0 | 298.15 | -44.80±0.71 | -2.62±0.47 |
| 0 | 298.15 | -43.30±0.71 | -1.60±0.47 |
| 0.100 | 298.15 | -44.92±0.62 | -2.92±0.44 |
| 0.500 | 298.15 | -46.53±0.68 | -3.22±0.51 |
| 0.500 | 298.15 | -46.40±0.68 | -3.52±0.51 |
| 1.000 | 298.15 | -48.10±1.34 | -4.12±0.84 |
| 0 | 308.15 | -44.36±0.71 | -1.32±0.32 |
| 0 | 318.15 | -43.91±0.71 | -0.45±0.30 |
| 0.500 | 318.15 | -45.51±0.68 | -1.30±0.38 |

^a^ ± 95 % C.I.

**Table 25S** Calculated thermodynamic parameters of L-Alanine at different temperatures, ionic strengths and in different ionic media

| *I* ^a^ | *T* / K | log $\text{K}_{\text{1}}^{\text{H}}$ | log $\text{K}_{\text{2}}^{\text{H}}$ | Δ$\text{H}_{\text{1}}^{\text{0}}$ ^b^ | | Δ$\text{H}_{\text{2}}^{\text{0}}$ ^b^ | | -Δ$\text{G}_{\text{1}}^{\text{0}}$ ^b^ | -Δ$\text{G}_{\text{2}}^{\text{0}}$ ^b^ | *T*Δ$\text{S}_{\text{1}}^{\text{0}}$ ^b^ | | *T*Δ$\text{S}_{\text{2}}^{\text{0}}$ ^b^ | |
| --- | --- | --- | --- | --- | --- | --- | --- | --- | --- | --- | --- | --- | --- |
|  |  | **NaCl** | | | | | | | | | | | |
| 0.00 | 283.15 | 10.329±0.014 ^c^ | 2.404±0.016 ^c^ | -44.87±0.32 ^c^ | | -4.19±0.34 ^c^ | | 55.99±0.08 ^c^ | 13.03±0.08 ^c^ | 11.12±0.32 ^c^ | | 8.84±0.34 ^c^ | |
| 0.10 | 283.15 | 10.156±0.010 | 2.415±0.014 | -45.59±0.28 | | -4.36±0.32 | | 55.05±0.06 | 13.09±0.08 | 9.46±0.28 | | 8.73±0.34 | |
| 0.50 | 283.15 | 10.154±0.010 | 2.461±0.010 | -46.83±0.30 | | -5.06±0.36 | | 55.04±0.06 | 13.34±0.06 | 8.22±0.30 | | 8.28±0.36 | |
| 1.00 | 283.15 | 10.233±0.014 | 2.523±0.010 | -48.13±0.56 | | -5.94±0.46 | | 55.47±0.08 | 13.67±0.06 | 7.34±0.45 | | 7.73±0.46 | |
| 3.00 | 283.15 | 10.626±0.028 | 2.777±0.018 | -53.02±1.92 | | -9.45±1.14 | | 57.60±0.14 | 15.06±0.10 | 4.58±1.94 | | 5.60±1.04 | |
| 5.00 | 283.15 | 11.042±0.038 | 3.036±0.024 | -57.80±3.34 | | -12.96±1.88 | | 59.85±0.20 | 16.46±0.12 | 2.05±3.34 | | 3.49±1.88 | |
| 0.00 | 298.15 | 9.912±0.012 | 2.365±0.016 | -44.24±0.32 | | -2.57±0.20 | | 56.58±0.08 | 13.50±0.08 | 12.34±0.32 | | 10.93±0.22 | |
| 0.10 | 298.15 | 9.727±0.010 | 2.374±0.014 | -45.12±0.28 | | -2.74±0.20 | | 55.52±0.06 | 13.55±0.08 | 10.40±0.28 | | 10.81±0.20 | |
| 0.50 | 298.15 | 9.712±0.010 | 2.414±0.010 | -46.45±0.28 | | -3.44±0.22 | | 55.43±0.06 | 13.78±0.06 | 8.98±0.28 | | 10.34±0.22 | |
| 1.00 | 298.15 | 9.777±0.012 | 2.467±0.010 | -47.80±0.56 | | -4.32±0.34 | | 55.81±0.08 | 14.08±0.06 | 8.01±0.56 | | 9.76±0.36 | |
| 3.00 | 298.15 | 10.123±0.012 | 2.690±0.014 | -52.75±1.92 | | -7.83±1.08 | | 57.78±0.12 | 15.35±0.08 | 5.03±1.92 | | 7.52±1.08 | |
| 5.00 | 298.15 | 10.493±0.024 | 2.916±0.016 | -57.55±3.32 | | -11.34±1.84 | | 59.89±0.14 | 16.64±0.10 | 2.34±3.34 | | 5.30±1.84 | |
| 0.00 | 310.15 | 9.616±0.014 | 2.357±0.016 | -43.75±0.32 | | -1.27±0.16 | | 57.10±0.08 | 13.99±0.10 | 13.35±0.32 | | 12.72±0.18 | |
| 0.15 | 310.15 | 9.402±0.010 | 2.369±0.012 | -44.98±0.26 | | -1.53±0.12 | | 55.83±0.06 | 14.07±0.08 | 10.85±0.26 | | 12.53±0.14 | |
| 0.50 | 310.15 | 9.391±0.010 | 2.400±0.010 | -46.15±0.28 | | -2.15±0.14 | | 55.76±0.06 | 14.25±0.06 | 9.61±0.28 | | 12.10±0.14 | |
| 1.00 | 310.15 | 9.446±0.014 | 2.447±0.010 | -47.53±0.56 | | -3.02±0.30 | | 56.09±0.08 | 14.53±0.06 | 8.56±0.56 | | 11.51±0.30 | |
| 3.00 | 310.15 | 9.756±0.024 | 2.645±0.016 | -52.53±1.92 | | -6.53±1.04 | | 57.93±0.14 | 15.71±0.10 | 5.40±1.94 | | 9.17±1.04 | |
| 5.00 | 310.15 | 10.093±0.032 | 2.848±0.020 | -57.35±3.32 | | -10.05±1.80 | | 59.93±0.20 | 16.91±0.12 | 2.58±3.34 | | 6.86±1.82 | |
|  |  | **(CH_3_)_4_NCl** | | | | | | | | | | | |
| 0.00 | 298.15 | 9.912±0.012 | 2.365±0.016 |  |  |  |  | 56.58±0.08 | 13.50±0.08 |  |  |  |  |
| 0.10 | 298.15 | 9.719±0.012 | 2.373±0.014 |  |  |  |  | 55.48±0.06 | 13.55±0.08 |  |  |  |  |
| 0.50 | 298.15 | 9.660±0.016 | 2.405±0.012 |  |  |  |  | 55.14±0.10 | 13.73±0.06 |  |  |  |  |
| 1.00 | 298.15 | 9.659±0.024 | 2.443±0.012 |  |  |  |  | 55.13±0.14 | 13.94±0.08 |  |  |  |  |
| 3.00 | 298.15 | 9.704±0.036 | 2.591±0.018 |  |  |  |  | 55.39±0.20 | 14.79±0.10 |  |  |  |  |
| 5.00 | 298.15 | 9.759±0.040 | 2.738±0.020 |  |  |  |  | 55.71±0.22 | 15.63±0.12 |  |  |  |  |

^a^ in mol Kg^-1^; ^b^ in kJ mol^-1^; ^c^ ± 95% C.I.

**Table 26S** Calculated thermodynamic parameters of Glycine at different temperatures, ionic strengths and in different ionic media

| *I* ^a^ | *T* / K | log $\text{K}_{\text{1}}^{\text{H}}$ | log $\text{K}_{\text{2}}^{\text{H}}$ | Δ$\text{H}_{\text{1}}^{\text{0}}$ ^b^ | | Δ$\text{H}_{\text{2}}^{\text{0}}$ ^b^ | | -Δ$\text{G}_{\text{1}}^{\text{0}}$ ^b^ | -Δ$\text{G}_{\text{2}}^{\text{0}}$ ^b^ | *T*Δ$\text{S}_{\text{1}}^{\text{0}}$ ^b^ | | *T*Δ$\text{S}_{\text{2}}^{\text{0}}$ ^b^ | |
| --- | --- | --- | --- | --- | --- | --- | --- | --- | --- | --- | --- | --- | --- |
|  |  | **NaCl** | | | | | | | | | | | |
| 0 | 283.15 | 10.194±0.004 ^c^ | 2.384±0.008 ^c^ | -44.95±0.06 ^c^ | | -6.03±0.02 ^c^ | | 55.26±0.02 ^c^ | 12.93±0.04 ^c^ | 10.31±0.06 ^c^ | | 6.89±0.04 ^c^ | |
| 0.10 | 283.15 | 10.016±0.004 | 2.395±0.006 | -45.53±0.06 | | -6.12±0.02 | | 54.29±0.02 | 12.98±0.04 | 8.76±0.06 | | 6.86±0.04 | |
| 0.50 | 283.15 | 9.999±0.006 | 2.436±0.006 | -46.21±0.06 | | -6.46±0.02 | | 54.20±0.04 | 13.20±0.04 | 7.99±0.06 | | 6.75±0.04 | |
| 1.00 | 283.15 | 10.063±0.010 | 2.488±0.010 | -46.82±0.06 | | -6.88±0.02 | | 54.55±0.06 | 13.49±0.06 | 7.73±0.08 | | 6.61±0.06 | |
| 3.00 | 283.15 | 10.412±0.014 | 2.698±0.014 | -48.94±0.10 | | -8.58±0.08 | | 56.44±0.08 | 14.63±0.08 | 7.51±0.12 | | 6.05±0.12 | |
| 5.00 | 283.15 | 10.789±0.016 | 2.910±0.016 | -50.94±0.16 | | -10.28±0.14 | | 58.48±0.08 | 15.77±0.06 | 7.55±0.18 | | 5.49±0.18 | |
| 0 | 298.15 | 9.777±0.004 | 2.329±0.008 | -44.33±0.02 | | -3.99±0.02 | | 55.81±0.02 | 13.29±0.04 | 11.48±0.04 | | 9.30±0.04 | |
| 0.10 | 298.15 | 9.588±0.004 | 2.338±0.006 | -45.07±0.02 | | -4.08±0.02 | | 54.73±0.02 | 13.34±0.04 | 9.66±0.04 | | 9.26±0.04 | |
| 0.50 | 298.15 | 9.562±0.006 | 2.376±0.006 | -45.84±0.02 | | -4.42±0.02 | | 54.58±0.04 | 13.56±0.04 | 8.74±0.04 | | 9.14±0.04 | |
| 1.00 | 298.15 | 9.619±0.010 | 2.424±0.010 | -46.49±0.04 | | -4.84±0.02 | | 54.91±0.06 | 13.84±0.06 | 8.41±0.05 | | 8.99±0.06 | |
| 3.00 | 298.15 | 9.946±0.014 | 2.619±0.014 | -48.67±0.08 | | -6.54±0.08 | | 56.77±0.08 | 14.95±0.08 | 8.11±0.12 | | 8.40±0.12 | |
| 5.00 | 298.15 | 10.304±0.016 | 2.814±0.016 | -50.69±0.14 | | -8.24±0.14 | | 58.81±0.10 | 16.06±0.10 | 8.12±0.18 | | 7.82±0.18 | |
| 0 | 310.15 | 9.480±0.004 | 2.312±0.008 | -43.84±0.06 | | -2.37±0.04 | | 56.29±0.02 | 13.73±0.04 | 12.45±0.06 | | 11.37±0.04 | |
| 0.15 | 310.15 | 9.262±0.004 | 2.326±0.006 | -44.86±0.06 | | -2.49±0.04 | | 54.99±0.02 | 13.81±0.04 | 10.14±0.06 | | 11.32±0.04 | |
| 0.50 | 310.15 | 9.246±0.006 | 2.357±0.006 | -45.55±0.06 | | -2.79±0.04 | | 54.90±0.04 | 13.99±0.04 | 9.35±0.06 | | 11.20±0.04 | |
| 1.00 | 310.15 | 9.297±0.010 | 2.402±0.010 | -46.23±0.06 | | -3.22±0.04 | | 55.20±0.06 | 14.26±0.06 | 8.97±0.08 | | 11.05±0.06 | |
| 3.00 | 310.15 | 9.608±0.014 | 2.585±0.014 | -48.45±0.10 | | -4.91±0.08 | | 57.05±0.08 | 15.35±0.08 | 8.60±0.12 | | 10.44±0.12 | |
| 5.00 | 310.15 | 9.950±0.016 | 2.770±0.016 | -50.49±0.16 | | -6.61±0.14 | | 59.08±0.10 | 16.44±0.10 | 8.59±0.18 | | 9.83±0.18 | |
|  |  | **(CH_3_)_4_NCl** | | | | | | | | | | | |
| 0 | 298.15 | 9.777±0.004 | 2.329±0.008 |  |  |  |  | 55.81±0.02 | 13.29±0.04 |  |  |  |  |
| 0.10 | 298.15 | 9.583±0.004 | 2.337±0.006 |  |  |  |  | 54.70±0.02 | 13.34±0.04 |  |  |  |  |
| 0.50 | 298.15 | 9.524±0.010 | 2.373±0.008 |  |  |  |  | 54.37±0.06 | 13.55±0.04 |  |  |  |  |
| 1.00 | 298.15 | 9.527±0.014 | 2.423±0.010 |  |  |  |  | 54.38±0.08 | 13.83±0.06 |  |  |  |  |
| 3.00 | 298.15 | 9.596±0.022 | 2.635±0.014 |  |  |  |  | 54.77±0.12 | 15.04±0.08 |  |  |  |  |
| 5.00 | 298.15 | 9.677±0.024 | 2.852±0.016 |  |  |  |  | 55.24±0.14 | 16.28±0.10 |  |  |  |  |

^a^ in mol Kg^-1^; ^b^ in kJ mol^-1^; ^c^ ± 95% C.I.

**Table 27S** Calculated thermodynamic parameters of L-Leucine at different temperatures, ionic strengths and in different ionic media

| *I* ^a^ | *T* / K | log $\text{K}_{\text{1}}^{\text{H}}$ | log $\text{K}_{\text{2}}^{\text{H}}$ | Δ$\text{H}_{\text{1}}^{\text{0}}$ ^b^ | Δ$\text{H}_{\text{2}}^{\text{0}}$ ^b^ | -Δ$\text{G}_{\text{1}}^{\text{0}}$ ^b^ | -Δ$\text{G}_{\text{2}}^{\text{0}}$ ^b^ | *T*Δ$\text{S}_{\text{1}}^{\text{0}}$ ^b^ | *T*Δ$\text{S}_{\text{2}}^{\text{0}}$ ^b^ |
| --- | --- | --- | --- | --- | --- | --- | --- | --- | --- |
|  |  | **NaCl** | | | | | | | |
| 0 | 283.15 | 10.204±0.005 ^c^ | 2.356±0.008 ^c^ | -45.89±0.06 ^c^ | -3.70±0.14 ^c^ | 55.31±0.05 ^c^ | 12.77±0.09 ^c^ | 9.42±0.16 ^c^ | 9.07±0.33 ^c^ |
| 0.10 | 283.15 | 10.032±0.004 | 2.364±0.007 | -46.65±0.06 | -3.78±0.14 | 54.38±0.05 | 12.82±0.08 | 7.73±0.14 | 9.04±0.32 |
| 0.50 | 283.15 | 10.043±0.004 | 2.412±0.005 | -48.04±0.06 | -4.10±0.14 | 54.44±0.04 | 13.08±0.05 | 6.40±0.14 | 8.97±0.30 |
| 1.00 | 283.15 | 10.146±0.005 | 2.487±0.004 | -49.54±0.07 | -4.51±0.15 | 55.00±0.05 | 13.48±0.04 | 5.46±0.17 | 8.97±0.31 |
| 3.00 | 283.15 | 10.661±0.007 | 2.829±0.006 | -55.21±0.17 | -6.15±0.24 | 57.79±0.08 | 15.33±0.06 | 2.59±0.36 | 9.19±0.48 |
| 5.00 | 283.15 | 11.208±0.008 | 3.189±0.007 | -60.76±0.27 | -7.78±0.36 | 60.76±0.09 | 17.29±0.07 | 0.00±0.56 | 9.51±0.72 |
| 0 | 298.15 | 9.778±0.005 | 2.321±0.008 | -45.33±0.09 | -1.86±0.18 | 55.81±0.06 | 13.25±0.09 | 10.48±0.21 | 11.39±0.40 |
| 0.10 | 298.15 | 9.594±0.004 | 2.329±0.007 | -46.25±0.09 | -1.94±0.18 | 54.76±0.05 | 13.30±0.08 | 8.51±0.19 | 11.35±0.38 |
| 0.50 | 298.15 | 9.589±0.004 | 2.374±0.005 | -47.73±0.08 | -2.27±0.16 | 54.73±0.04 | 13.55±0.05 | 7.00±0.17 | 11.28±0.33 |
| 1.00 | 298.15 | 9.677±0.004 | 2.445±0.004 | -49.27±0.08 | -2.68±0.14 | 55.23±0.05 | 13.95±0.05 | 5.96±0.18 | 11.27±0.29 |
| 3.00 | 298.15 | 10.137±0.007 | 2.772±0.006 | -54.99±0.15 | -4.31±0.15 | 57.86±0.08 | 15.82±0.06 | 2.87±0.32 | 11.51±0.31 |
| 5.00 | 298.15 | 10.632±0.008 | 3.117±0.007 | -60.57±0.25 | -5.95±0.25 | 60.69±0.09 | 17.79±0.07 | 0.12±0.52 | 11.84±0.51 |
| 0 | 310.15 | 9.474±0.005 | 2.319±0.008 | -44.89±0.14 | -0.40±0.33 | 56.25±0.06 | 13.77±0.10 | 11.37±0.31 | 13.37±0.67 |
| 0.15 | 310.15 | 9.261±0.004 | 2.330±0.007 | -46.17±0.14 | -0.52±0.32 | 54.99±0.05 | 13.84±0.08 | 8.82±0.29 | 13.32±0.65 |
| 0.50 | 310.15 | 9.260±0.004 | 2.369±0.005 | -47.48±0.13 | -0.81±0.30 | 54.98±0.04 | 14.06±0.06 | 7.50±0.27 | 13.26±0.61 |
| 1.00 | 310.15 | 9.335±0.005 | 2.436±0.004 | -49.05±0.13 | -1.21±0.28 | 55.43±0.05 | 14.47±0.05 | 6.38±0.27 | 13.25±0.56 |
| 3.00 | 310.15 | 9.755±0.007 | 2.752±0.005 | -54.82±0.16 | -2.85±0.24 | 57.92±0.08 | 16.34±0.06 | 3.10±0.35 | 13.50±0.48 |
| 5.00 | 310.15 | 10.211±0.008 | 3.086±0.006 | -60.42±0.25 | -4.48±0.27 | 60.63±0.09 | 18.33±0.07 | 0.22±0.52 | 13.85±0.54 |
|  |  | **(CH_3_)_4_NCl** | | | | | | | |
| 0 | 298.15 | 9.778±0.005 | 2.321±0.008 | -45.33±0.09 | -1.86±0.18 | 55.81±0.06 | 13.25±0.09 | 10.48±0.21 | 11.39±0.40 |
| 0.10 | 298.15 | 9.585±0.005 | 2.328±0.008 | -45.98±0.08 | -2.04±0.17 | 54.71±0.05 | 13.29±0.08 | 8.73±0.19 | 11.25±0.38 |
| 0.50 | 298.15 | 9.529±0.006 | 2.359±0.012 | -46.39±0.08 | -2.73±0.15 | 54.39±0.07 | 13.46±0.14 | 8.00±0.20 | 10.73±0.40 |
| 1.00 | 298.15 | 9.535±0.009 | 2.400±0.018 | -46.58±0.09 | -3.60±0.16 | 54.43±0.10 | 13.70±0.20 | 7.84±0.26 | 10.10±0.50 |
| 3.00 | 298.15 | 9.618±0.014 | 2.576±0.026 | -46.93±0.22 | -7.06±0.40 | 54.90±0.16 | 14.70±0.30 | 7.96±0.53 | 7.64±0.98 |
| 5.00 | 298.15 | 9.713±0.016 | 2.756±0.029 | -47.13±0.38 | -10.53±0.71 | 55.44±0.17 | 15.73±0.33 | 8.31±0.82 | 5.20±1.53 |

^a^ in mol Kg^-1^; ^b^ in kJ mol^-1^; ^c^ ± 95% C.I.

**Table 28S** Calculated thermodynamic parameters of L-Phenylalanine at different temperatures, ionic strengths and in different ionic media

| *I* ^a^ | *T* / K | log $\text{K}_{\text{1}}^{\text{H}}$ | log $\text{K}_{\text{2}}^{\text{H}}$ | Δ$\text{H}_{\text{1}}^{\text{0}}$ ^b^ | Δ$\text{H}_{\text{2}}^{\text{0}}$ ^b^ | -Δ$\text{G}_{\text{1}}^{\text{0}}$ ^b^ | -Δ$\text{G}_{\text{2}}^{\text{0}}$ ^b^ | *T*Δ$\text{S}_{\text{1}}^{\text{0}}$ ^b^ | *T*Δ$\text{S}_{\text{2}}^{\text{0}}$ ^b^ |
| --- | --- | --- | --- | --- | --- | --- | --- | --- | --- |
|  |  | **NaCl** | | | | | | | |
| 0 | 283.15 | 9.672±0.006 ^c^ | 2.159±0.007 ^c^ | -44.63±0.18 ^c^ |  | 52.43±0.07 ^c^ | 11.70±0.08 ^c^ | 7.80±0.38 ^c^ |  |
| 0.10 | 283.15 | 9.496±0.006 | 2.174±0.004 | -45.26±0.18 |  | 51.48±0.06 | 11.79±0.05 | 6.22±0.37 |  |
| 0.50 | 283.15 | 9.482±0.014 | 2.250±0.014 | -46.08±0.16 |  | 51.40±0.15 | 12.20±0.15 | 5.32±0.43 |  |
| 1.00 | 283.15 | 9.542±0.022 | 2.360±0.023 | -46.87±0.16 |  | 51.72±0.24 | 12.80±0.25 | 4.86±0.55 |  |
| 3.00 | 283.15 | 9.854±0.034 | 2.853±0.037 | -49.70±0.32 |  | 53.41±0.36 | 15.46±0.40 | 3.72±0.95 |  |
| 5.00 | 283.15 | 10.185±0.038 | 3.365±0.042 | -52.41±0.55 |  | 55.21±0.41 | 18.24±0.45 | 2.80±1.35 |  |
| 0 | 298.15 | 9.258±0.006 | 2.144±0.007 | -44.06±0.15 | -1.64±0.16 | 52.84±0.07 | 12.24±0.08 | 8.78±0.33 | 10.60±0.35 |
| 0.10 | 298.15 | 9.071±0.006 | 2.157±0.004 | -44.84±0.15 | -1.85±0.16 | 51.78±0.06 | 12.31±0.05 | 6.94±0.31 | 10.46±0.32 |
| 0.50 | 298.15 | 9.046±0.014 | 2.225±0.014 | -45.75±0.12 | -2.72±0.14 | 51.63±0.16 | 12.70±0.16 | 5.88±0.40 | 9.98±0.41 |
| 1.00 | 298.15 | 9.097±0.022 | 2.325±0.023 | -46.58±0.12 | -3.80±0.13 | 51.93±0.25 | 13.27±0.26 | 5.35±0.54 | 9.47±0.57 |
| 3.00 | 298.15 | 9.381±0.034 | 2.777±0.037 | -49.47±0.31 | -8.13±0.21 | 53.55±0.38 | 15.85±0.42 | 4.08±0.96 | 7.72±0.92 |
| 5.00 | 298.15 | 9.686±0.038 | 3.249±0.042 | -52.21±0.55 | -12.46±0.37 | 55.29±0.43 | 18.55±0.47 | 3.08±1.36 | 6.09±1.17 |
| 0 | 310.15 | 8.962±0.007 | 2.133±0.007 | -43.60±0.17 |  | 53.22±0.08 | 12.66±0.08 | 9.62±0.37 |  |
| 0.15 | 310.15 | 8.747±0.006 | 2.151±0.005 | -44.67±0.16 |  | 51.93±0.07 | 12.77±0.05 | 7.26±0.35 |  |
| 0.50 | 310.15 | 8.730±0.014 | 2.206±0.014 | -45.49±0.15 |  | 51.84±0.17 | 13.10±0.16 | 6.35±0.43 |  |
| 1.00 | 310.15 | 8.774±0.022 | 2.299±0.023 | -46.35±0.14 |  | 52.10±0.26 | 13.65±0.27 | 5.75±0.58 |  |
| 3.00 | 310.15 | 9.036±0.034 | 2.722±0.037 | -49.29±0.31 |  | 53.66±0.40 | 16.16±0.44 | 4.37±1.00 |  |
| 5.00 | 310.15 | 9.322±0.039 | 3.165±0.042 | -52.04±0.55 |  | 55.35±0.45 | 18.79±0.49 | 3.31±1.39 |  |
|  |  | **(CH_3_)_4_NCl** | | | | | | | |
| 0 | 298.15 | 9.258±0.006 | 2.144±0.007 |  |  | 52.84±0.07 | 12.24±0.08 |  |  |
| 0.10 | 298.15 | 9.062±0.008 | 2.154±0.008 |  |  | 51.72±0.08 | 12.29±0.09 |  |  |
| 0.50 | 298.15 | 8.993±0.019 | 2.199±0.017 |  |  | 51.33±0.21 | 12.55±0.19 |  |  |
| 1.00 | 298.15 | 8.981±0.028 | 2.262±0.025 |  |  | 51.26±0.31 | 12.91±0.28 |  |  |
| 3.00 | 298.15 | 8.986±0.041 | 2.541±0.037 |  |  | 51.29±0.46 | 14.50±0.42 |  |  |
| 5.00 | 298.15 | 9.003±0.046 | 2.828±0.041 |  |  | 51.39±0.51 | 16.14±0.46 |  |  |

^a^ in mol Kg^-1^; ^b^ in kJ mol^-1^; ^c^ ± 95% C.I.

**Table 29S** Calculated thermodynamic parameters of L-Serine at different temperatures, ionic strengths and in different ionic media

| *I* ^a^ | *T* / K | log $\text{K}_{\text{1}}^{\text{H}}$ | log $\text{K}_{\text{2}}^{\text{H}}$ | Δ$\text{H}_{\text{1}}^{\text{0}}$ ^b^ | | Δ$\text{H}_{\text{2}}^{\text{0}}$ ^b^ | | -Δ$\text{G}_{\text{1}}^{\text{0}}$ ^b^ | -Δ$\text{G}_{\text{2}}^{\text{0}}$ ^b^ | *T*Δ$\text{S}_{\text{1}}^{\text{0}}$ ^b^ | | *T*Δ$\text{S}_{\text{2}}^{\text{0}}$ ^b^ | |
| --- | --- | --- | --- | --- | --- | --- | --- | --- | --- | --- | --- | --- | --- |
|  |  | **NaCl** | | | | | | | | | | | |
| 0 | 283.15 | 9.657±0.009 ^c^ | 2.223±0.006 ^c^ | -43.39±0.06 ^c^ | | -6.59±0.21 ^c^ | | 52.35±0.09 ^c^ | 12.05±0.06 ^c^ | 8.96±0.21 ^c^ | | 5.46±0.44 ^c^ | |
| 0.10 | 283.15 | 9.472±0.008 | 2.223±0.006 | -43.00±0.08 | | -5.86±0.32 | | 51.35±0.08 | 12.05±0.06 | 8.35±0.22 | | 6.19±0.63 | |
| 0.50 | 283.15 | 9.423±0.008 | 2.226±0.017 | -39.78±0.53 | | -2.93±1.52 | | 51.08±0.08 | 12.07±0.18 | 11.30±1.06 | | 9.13±3.01 | |
| 1.00 | 283.15 | 9.444±0.012 | 2.234±0.032 |  |  |  |  | 51.19±0.13 | 12.11±0.34 |  |  |  |  |
| 3.00 | 283.15 | 9.611±0.033 | 2.282±0.091 |  |  |  |  | 52.10±0.35 | 12.37±0.96 |  |  |  |  |
| 5.00 | 283.15 | 9.803±0.054 | 2.335±0.149 |  |  |  |  | 53.14±0.58 | 12.66±1.58 |  |  |  |  |
| 0 | 298.15 | 9.255±0.009 | 2.162±0.005 | -42.78±0.05 | | -4.48±0.20 | | 52.83±0.10 | 12.34±0.06 | 10.05±0.22 | | 7.87±0.41 | |
| 0.10 | 298.15 | 9.068±0.007 | 2.169±0.005 | -42.54±0.08 | | -3.74±0.25 | | 51.76±0.08 | 12.38±0.05 | 9.22±0.22 | | 8.64±0.50 | |
| 0.50 | 298.15 | 9.046±0.006 | 2.199±0.009 | -39.42±0.53 | | -0.82±1.47 | | 51.63±0.07 | 12.55±0.10 | 12.22±1.05 | | 11.73±2.88 | |
| 1.00 | 298.15 | 9.105±0.007 | 2.241±0.013 |  |  |  |  | 51.97±0.07 | 12.79±0.14 |  |  |  |  |
| 3.00 | 298.15 | 9.431±0.010 | 2.424±0.020 |  |  |  |  | 53.83±0.11 | 13.84±0.22 |  |  |  |  |
| 5.00 | 298.15 | 9.785±0.011 | 2.613±0.022 |  |  |  |  | 55.85±0.12 | 14.92±0.25 |  |  |  |  |
| 0 | 310.15 | 8.968±0.009 | 2.143±0.006 | -42.29±0.05 | | -2.78±0.30 | | 53.25±0.10 | 12.73±0.07 | 10.96±0.22 | | 9.94±0.59 | |
| 0.15 | 310.15 | 8.763±0.007 | 2.161±0.005 | -41.85±0.13 | | -1.69±0.40 | | 52.03±0.08 | 12.83±0.06 | 10.18±0.31 | | 11.14±0.79 | |
| 0.50 | 310.15 | 8.773±0.007 | 2.205±0.012 | -39.12±0.54 | | 0.87±1.44 | | 52.09±0.08 | 13.09±0.14 | 12.97±1.06 | | 13.96±2.84 | |
| 1.00 | 310.15 | 8.859±0.010 | 2.272±0.023 |  |  |  |  | 52.61±0.12 | 13.49±0.27 |  |  |  |  |
| 3.00 | 310.15 | 9.301±0.025 | 2.554±0.065 |  |  |  |  | 55.23±0.29 | 15.17±0.76 |  |  |  |  |
| 5.00 | 310.15 | 9.772±0.040 | 2.842±0.107 |  |  |  |  | 58.02±0.47 | 16.88±1.25 |  |  |  |  |
|  |  | **(CH_3_)_4_NCl** | | | | | | | | | | | |
| 0 | 298.15 | 9.255±0.009 | 2.162±0.005 |  |  |  |  | 52.83±0.10 | 12.34±0.06 |  |  |  |  |
| 0.10 | 298.15 | 9.062±0.008 | 2.173±0.004 |  |  |  |  | 51.72±0.09 | 12.40±0.04 |  |  |  |  |
| 0.50 | 298.15 | 9.006±0.014 | 2.216±0.014 |  |  |  |  | 51.40±0.16 | 12.65±0.16 |  |  |  |  |
| 1.00 | 298.15 | 9.011±0.021 | 2.273±0.023 |  |  |  |  | 51.43±0.23 | 12.97±0.26 |  |  |  |  |
| 3.00 | 298.15 | 9.086±0.031 | 2.507±0.037 |  |  |  |  | 51.86±0.34 | 14.31±0.41 |  |  |  |  |
| 5.00 | 298.15 | 9.174±0.034 | 2.743±0.041 |  |  |  |  | 52.37±0.38 | 15.66±0.46 |  |  |  |  |

^a^ in mol Kg^-1^; ^b^ in kJ mol^-1^; ^c^ ± 95% C.I.

**Table 30S** Calculated thermodynamic parameters of L-Valine at different temperatures, ionic strengths and in different ionic media

| *I* ^a^ | *T* / K | log $\text{K}_{\text{1}}^{\text{H}}$ | log $\text{K}_{\text{2}}^{\text{H}}$ | Δ$\text{H}_{\text{1}}^{\text{0}}$ ^b^ | | Δ$\text{H}_{\text{2}}^{\text{0}}$ ^b^ | | -Δ$\text{G}_{\text{1}}^{\text{0}}$ ^b^ | -Δ$\text{G}_{\text{2}}^{\text{0}}$ ^b^ | *T*Δ$\text{S}_{\text{1}}^{\text{0}}$ ^b^ | | *T*Δ$\text{S}_{\text{2}}^{\text{0}}$ ^b^ | |
| --- | --- | --- | --- | --- | --- | --- | --- | --- | --- | --- | --- | --- | --- |
|  |  | **NaCl** | | | | | | | | | | | |
| 0 | 283.15 | 10.148±0.008 ^c^ | 2.310±0.005 ^c^ | -45.04±0.10 ^c^ | | -2.58±0.19 ^c^ | | 55.01±0.09 ^c^ | 12.52±0.06 ^c^ | 9.97±0.26 ^c^ | | 9.95±0.38 ^c^ | |
| 0.10 | 283.15 | 9.979±0.006 | 2.316±0.008 | -45.70±0.09 | | -2.39±0.58 | | 54.09±0.06 | 12.55±0.09 | 8.39±0.22 | | 10.16±1.15 | |
| 0.50 | 283.15 | 9.985±0.018 | 2.340±0.035 | -46.69±0.16 | | -1.66±2.88 | | 54.13±0.19 | 12.68±0.38 | 7.44±0.49 | | 11.02±5.69 | |
| 1.00 | 283.15 | 10.067±0.029 | 2.372±0.065 | -47.68±0.31 | | -0.75±5.78 | | 54.57±0.30 | 12.86±0.69 | 6.89±0.86 | | 12.11±11.40 | |
| 3.00 | 283.15 | 10.449±0.046 | 2.510±0.173 | -51.33±0.98 | |  |  | 56.64±0.49 | 13.61±1.84 | 5.32±2.15 | |  |  |
| 5.00 | 283.15 | 10.845±0.053 | 2.651±0.280 | -54.86±1.65 | |  |  | 58.79±0.56 | 14.37±2.98 | 3.93±3.42 | |  |  |
| 0 | 298.15 | 9.730±0.008 | 2.286±0.005 | -44.46±0.09 | | -0.33±0.13 | | 55.54±0.09 | 13.05±0.06 | 11.08±0.26 | | 12.72±0.27 | |
| 0.10 | 298.15 | 9.549±0.006 | 2.293±0.006 | -45.27±0.09 | | -0.15±0.57 | | 54.51±0.07 | 13.09±0.07 | 9.23±0.22 | | 12.94±1.12 | |
| 0.50 | 298.15 | 9.544±0.018 | 2.324±0.020 | -46.35±0.16 | | 0.58±2.88 | | 54.48±0.20 | 13.27±0.22 | 8.12±0.50 | | 13.85±5.66 | |
| 1.00 | 298.15 | 9.615±0.028 | 2.365±0.031 | -47.39±0.31 | | 1.49±5.78 | | 54.88±0.32 | 13.50±0.34 | 7.49±0.87 | | 14.99±11.35 | |
| 3.00 | 298.15 | 9.961±0.045 | 2.537±0.047 | -51.09±0.98 | |  |  | 56.86±0.50 | 14.48±0.52 | 5.77±2.16 | |  |  |
| 5.00 | 298.15 | 10.324±0.050 | 2.712±0.052 | -54.64±1.65 | |  |  | 58.93±0.56 | 15.48±0.59 | 4.28±3.43 | |  |  |
| 0 | 310.15 | 9.432±0.008 | 2.296±0.005 | -43.99±0.10 | | 1.46±0.13 | | 56.01±0.10 | 13.63±0.06 | 12.02±0.27 | | 15.10±0.28 | |
| 0.15 | 310.15 | 9.225±0.007 | 2.309±0.009 | -45.13±0.09 | | 1.74±0.86 | | 54.77±0.08 | 13.71±0.10 | 9.65±0.24 | | 15.45±1.70 | |
| 0.50 | 310.15 | 9.224±0.018 | 2.340±0.026 | -46.08±0.16 | | 2.38±2.88 | | 54.77±0.21 | 13.90±0.30 | 8.69±0.51 | | 16.27±5.68 | |
| 1.00 | 310.15 | 9.286±0.029 | 2.388±0.047 | -47.15±0.31 | | 3.29±5.78 | | 55.14±0.33 | 14.18±0.54 | 7.99±0.90 | | 17.47±11.39 | |
| 3.00 | 310.15 | 9.606±0.045 | 2.584±0.121 | -50.90±0.98 | |  |  | 57.04±0.53 | 15.34±1.41 | 6.14±2.18 | |  |  |
| 5.00 | 310.15 | 9.943±0.052 | 2.783±0.197 | -54.47±1.65 | |  |  | 59.04±0.60 | 16.53±2.29 | 4.57±3.45 | |  |  |
|  |  | **(CH_3_)_4_NCl** | | | | | | | | | | | |
| 0 | 298.15 | 9.730±0.008 | 2.286±0.005 |  |  |  |  | 55.54±0.09 | 13.05±0.06 |  |  |  |  |
| 0.10 | 298.15 | 9.536±0.008 | 2.293±0.007 |  |  |  |  | 54.43±0.09 | 13.09±0.08 |  |  |  |  |
| 0.50 | 298.15 | 9.473±0.011 | 2.322±0.019 |  |  |  |  | 54.07±0.12 | 13.25±0.21 |  |  |  |  |
| 1.00 | 298.15 | 9.470±0.015 | 2.362±0.028 |  |  |  |  | 54.05±0.17 | 13.48±0.31 |  |  |  |  |
| 3.00 | 298.15 | 9.510±0.022 | 2.536±0.042 |  |  |  |  | 54.28±0.25 | 14.48±0.47 |  |  |  |  |
| 5.00 | 298.15 | 9.563±0.025 | 2.716±0.046 |  |  |  |  | 54.58±0.27 | 15.50±0.52 |  |  |  |  |

^a^ in mol Kg^-1^; ^b^ in kJ mol^-1^; ^c^ ± 95% C.I.

**Table 31S** Total and specific solubility of L-leucine in NaCl in the molar and in the molal concentration scales at *T* = 298.15 K

| *I* / mol dm^-3^ | log *S*^T^_c_ | log *S*^0^_c_ | *I* / mol kg^-1^ | log *S*^T^_m_ | log *S*^0^_m_ |
| --- | --- | --- | --- | --- | --- |
| 0.056 | -0.787±0.001 ^a^ | -0.788±0.001 | 0.056 | -0.786±0.001 ^a^ | -0.786±0.001 |
| 0.056 | -0.783±0.001 | -0.783±0.001 | 0.056 | -0.782±0.001 | -0.782±0.001 |
| 0.056 | -0.786±0.001 | -0.786±0.001 | 0.056 | -0.784±0.001 | -0.784±0.001 |
| 0.056 | -0.786±0.001 | -0.786±0.001 | 0.056 | -0.784±0.001 | -0.784±0.001 |
| 0.133 | -0.791±0.001 | -0.792±0.001 | 0.134 | -0.789±0.001 | -0.789±0.001 |
| 0.133 | -0.791±0.001 | -0.792±0.001 | 0.134 | -0.789±0.001 | -0.790±0.001 |
| 0.133 | -0.793±0.001 | -0.794±0.001 | 0.134 | -0.790±0.001 | -0.791±0.001 |
| 0.133 | -0.792±0.001 | -0.793±0.001 | 0.134 | -0.790±0.001 | -0.791±0.001 |
| 0.445 | -0.814±0.000 | -0.815±0.001 | 0.450 | -0.810±0.001 | -0.810±0.001 |
| 0.445 | -0.814±0.000 | -0.814±0.001 | 0.450 | -0.809±0.001 | -0.809±0.001 |
| 0.445 | -0.814±0.000 | -0.814±0.001 | 0.450 | -0.809±0.001 | -0.809±0.001 |
| 0.848 | -0.851±0.001 | -0.852±0.001 | 0.864 | -0.843±0.001 | -0.844±0.001 |
| 0.848 | -0.851±0.001 | -0.852±0.001 | 0.864 | -0.843±0.001 | -0.844±0.001 |
| 0.848 | -0.848±0.001 | -0.849±0.001 | 0.864 | -0.840±0.001 | -0.841±0.001 |
| 1.209 | -0.893±0.001 | -0.894±0.001 | 1.240 | -0.882±0.001 | -0.882±0.001 |
| 1.209 | -0.890±0.001 | -0.893±0.001 | 1.240 | -0.879±0.001 | -0.882±0.001 |
| 1.701 | -0.941±0.001 | -0.942±0.001 | 1.763 | -0.925±0.002 | -0.927±0.001 |
| 1.701 | -0.937±0.001 | -0.940±0.001 | 1.763 | -0.921±0.002 | -0.925±0.001 |
| 2.609 | -1.043±0.001 | -1.049±0.002 | 2.757 | -1.019±0.002 | -1.025±0.001 |
| 2.609 | -1.038±0.001 | -1.044±0.002 | 2.757 | -1.014±0.002 | -1.020±0.001 |
| 4.546 | -1.270±0.003 | -1.276±0.004 | 5.035 | -1.226±0.004 | -1.232±0.003 |
| 4.546 | -1.262±0.003 | -1.268±0.004 | 5.035 | -1.217±0.004 | -1.224±0.003 |

^a^ ± 95 % C.I.

**Table 32S** Total and specific solubility of L-phenylalanine in (CH_3_)_4_NCl and in NaCl in the molar and in the molal concentration scales at *T* = 298.15 K

| Medium | *I* / mol dm^-3^ | log *S*^T^_c_ ≈ log *S*^0^_c_ | *I* / mol kg^-1^ | log *S*^T^_m_ ≈ log *S*^0^_m_ |
| --- | --- | --- | --- | --- |
| (CH_3_)_4_NCl | 0.069 | -1.076±0.010 | 0.070 | -1.071 |
|  | 0.069 | -1.076±0.010 | 0.070 | -1.071 |
|  | 0.094 | -1.148±0.010 | 0.095 | -1.142 |
|  | 0.094 | -1.148±0.010 | 0.095 | -1.142 |
|  | 0.549 | -1.187±0.009 | 0.585 | -1.159 |
|  | 0.532 | -1.194±0.009 | 0.566 | -1.167 |
|  | 0.536 | -1.194±0.009 | 0.571 | -1.167 |
|  | 1.089 | -1.191±0.008 | 1.237 | -1.136 |
|  | 1.185 | -1.196±0.008 | 1.362 | -1.135 |
|  | 1.191 | -1.183±0.008 | 1.370 | -1.122 |
|  | 2.425 | -1.336±0.011 | 3.285 | -1.204 |
|  | 2.234 | -1.360±0.011 | 2.946 | -1.240 |
|  | 2.387 | -1.362±0.011 | 3.216 | -1.232 |
|  | 2.969 | -1.447±0.014 | 4.361 | -1.280 |
|  | 2.952 | -1.472±0.014 | 4.325 | -1.306 |
|  | 2.960 | -1.472±0.014 | 4.342 | -1.306 |
| NaCl | 0.101 | -1.155±0.010 | 0.101 | -1.153 |
|  | 0.101 | -1.125±0.010 | 0.101 | -1.123 |
|  | 0.492 | -1.080±0.009 | 0.498 | -1.075 |
|  | 0.491 | -1.114±0.009 | 0.497 | -1.109 |
|  | 0.493 | -1.029±0.009 | 0.499 | -1.024 |
|  | 0.988 | -1.113±0.008 | 1.009 | -1.104 |
|  | 0.988 | -1.119±0.008 | 1.009 | -1.110 |
|  | 0.978 | -1.119±0.008 | 0.999 | -1.110 |
|  | 1.980 | -1.224±0.011 | 2.064 | -1.206 |
|  | 1.961 | -1.205±0.011 | 2.043 | -1.187 |
|  | 1.942 | -1.207±0.010 | 2.023 | -1.189 |
|  | 2.980 | -1.309±0.016 | 3.176 | -1.281 |
|  | 2.958 | -1.283±0.016 | 3.151 | -1.256 |
|  | 2.940 | -1.303±0.016 | 3.130 | -1.276 |

^a^ ± 95 % C.I.

**References**

Anderson KP, Greenhalgh WO, Butler EA (1967) Formation constant, enthalpy, and entropy values for the association of nickel(II) ion with glycinate, alanate, and phenylalanate ions at 10, 25, and 40.degree. Inorg. Chem. 6: 1056-1058.

Anderson KP, Newell DA, Izatt RM (1966) Formation Constant, Enthalpy, and Entropy Values for the Association of Alanine with H+ and Cu2+ at 10, 25, and 40 °. Inorg. Chem. 5: 62-65.

Arena G, Cali R, Cucinotta V, Musumeci S, Rizzarelli E, Sammartano S (1983) Thermodynamics of metal complexes with ligand-ligand interaction, simple and mixed complexes of copper(II) and zinc(II) with adenosine 5[prime or minute]-triphosphate and L-tryptophan or L-alanine. J. Chem. Soc. Dalton. Trans. 1271-1278.

Avedikian L (1967) Bull. Soc. Chim. Fr. 254.

Aziz A, Lyle SJ (1971) Americium(III) and europium(III) complexes with lactate, pyruvate and α-alaninate in aqueous solutions—A comparison of equilibrium constants. J. Inorg. Nucl. Chem. 33: 3407-3408.

Berezina LP, Samoilenko VG, Pozukhin AI (1973) Zh. Neorg. Khim. 18: 393.

Berthon G (1995) The Stability Constants Of Metal Complexes Of Amino Acids With Polar Side Chains. Pure Appl. Chem. 67: 1117-1240.

Berthon G, Piktas M, Blais M-J (1984) Trace metal requirements in total parenteral nutrition. Part 6. A quantitative study of the copper(II)-histidine ternary complexes with leucine, glutamic acid, methionine, tryptophan and alanine, and final evaluation of the daily doses of copper and zinc specific to a nutritive mixture of a given composition. Inorg. Chim. Acta 93: 117-130.

Bonnet MC, Paris RA, Martin RP (1972) Bull. Soc. Chim. Fr. 909.

Brandariz I, Fiol S, Herrero R, Vilarino T, Sastre de Vicente M (1993) Protonation constants of α-Alanine, γ-Aminobutyric acid, and ε-Aminocaproic Acid. J. Chem. Eng. Data 38: 531-533.

Chandel CPS, Gupta CM (1982) Chem. Scripta 24: 229.

Chandel CPS, Gupta CM (1984) Mixed Chelates of Cadmium(II) with N-(2-Hydroxyethyl)ethylenediamine and Some Amino Acids. Bull. Chim. Soc. Jpn. 57: 2303-2306.

Chidambaram MV, Bhattacharya PK (1970) J. Indian Chem. Soc 47: 881.

Childs CW, Perrin DD (1969) Equilibria in solutions which contain a metal ion and an amino-acid. J. Chem. Soc. A: Inorganic, Physical, Theoretical 1039-1044.

Christensen JJ, Hansen LD, Izatt RM (1976) Handbook of proton ionization heats. John Wiley & Sons.

Christensen JJ, Oscarson JL, Izatt RM (1968) Thermodynamics of proton ionization in dilute aqueous solution. X. .DELTA.G.deg. (pK), .DELTA.H.deg., and .DELTA.S.deg. values for proton ionization from several monosubstituted carboxylic acids at 10, 25, and 40.deg. J. Am. Chem. Soc. 90: 5949-5953.

Doğan A, Köseoğlu F, Kilic E (2002) Studies on the macroscopic protonation constants of some α-amino acids in ethanol-water mixtures. Anal. Biochem. 309: 75-78.

Enea O, Berthon G, Cromer-Morin M, Scharff J-P (1979) Thermodynamique des complexes metalliques ternaires des amino acides: Etude du systeme Ni(II)—Glycine—DL-α-alanine. Thermochim. Acta 33: 311-322.

Felty WL, Ekstrom CG, Leussing DL (1970) Equilibrium studies involving Schiff base complexes. The zinc(II)-pyridoxal phosphate-glycine and -.alpha.-alanine systems. J. Am. Chem. Soc. 92: 3006-3011.

Fischer BE, Sigel H (1980) Ternary complexes in solution. 35. Intramolecular hydrophobic ligand-ligand interactions in mixed ligand complexes containing an aliphatic amino acid. J. Am. Chem. Soc. 102: 2998-3008.

Friedman JD, Levina MG (1974) Zh. Neorg. Khim. 19: 2422.

Gergely A, Kiraly R, Nagypal I, Mojzes J (1971) Acta Chim. Acad. Sci. Hung. 67: 133.

Gergely A, Nagypaál I, Kiss T, Kiraly R (1974a) Acta Chim. Acad. Sci. Hung. 82: 257.

Gergely A, Nagypal I, Farkas E (1974b) Magy. Kém. Foly. 80: 25.

Gergely A, Sóvágó I (1973) Log β, ΔH and ΔS values of mixed complexes of Cu(II) with histamine and some aliphatic aminoacids. J. Inorg. Nucl. Chem. 35: 4355-4365.

Gergely A, Sóvágó I, Nagypaál I, Király R (1972) Equilibrium relations of alpha-aminoacid mixed complexes of transition metal ions. Inorg. Chim. Acta 6: 435-439.

Gharib F, Nejhad BN, Nouri N (2015) Tautomeric and Microscopic Protonation Constants of Alanine and Valine in Different Aqueous Solutions of Methanol and Tetrahydrofuran. J. Solution Chem. 44: 1655-1672.

Gillard RD, Irving HM, Parkins RM, Payne NC, Pettit LD (1966) The isomers of complexes of [small alpha]-amino-acids with copper(II). J. Chem. Soc. A: Inorganic, Physical, Theoretical 1159-1164.

Griesser R, Prijs B, Sigel H, Foery W, Wright LD, McCormick DB (1970) Stability and structure of binary and ternary metal ion complexes with biocytin, the sulfoxide and sulfone, N-acetyl-L-lysine, and L-alanine. Biochemistry 9: 3285-3293.

Hamborg ES, Niederer JPM, F. VG (2007) Dissociation Constants and Thermodynamic Properties of Amino Acids used in CO_2_ Absorption from (293 to 353) K. J. Chem. Eng. Data 52: 2491-2502.

Heijne GJM, van der Linden WE (1975) Determination of stability constants of cadmium(II) with some amino-acids by use of an ion-selective electrode. Talanta 22: 923-925.

Irving H, Pettit LD (1963) 292. The stabilities of metal complexes of some C-substituted derivatives of glycine. J. Chem. Soc. (Resumed) 1546-1553.

Izatt RM, Wrathall JW, Andersen KP (1961) Studies Of The Copper(II)—Alanine And Phenylalanine Systems In Aqueous Solution. Dissociation And Formation Constants As A Function Of Temperature. J. Phys. Chem. 65: 1914-1915.

Jawaid M, Ingman F, Liem DH (1978) Studies on the Hydrolysis of Methylmercury(II) and its Complex Formation with Some Aliphatic Carboxylic and Aminocarboxylic Acids. Acta Chem. Slov. 32A: 333-343.

Korsunov IA, Sergeev GM (1971) Radiokhimiya 13: 901.

Kurganov AA, Davankov VA, Koreskov JD, Rogozin SV (1977) Soviet. J. Coord. Chim. 3: 667.

Leussing DL, Hanna EM (1966) Metal Ion Catalysis in Transamination. III. Nickel(II) and Zinc(II) Mixed Complexes Involving Pyruvate and Various Substituted Aliphatic Amino Acids1. J. Am. Chem. Soc. 88: 693-696.

Leussing DL, Leach BE (1971) Stabilities, rates of formation, and rates of transimination in aqueous solutions of some zinc(II)-Schiff base complexes derived from salicylaldehyde. J. Am. Chem. Soc. 93: 3377-3384.

Lim MC (1978) Mixed-ligand complexes of palladium(II). Part 3. Diaqua(ethylene-diamine) palladium(II) complexes of L-amino-acids. J. Chem. Soc. Dalton. Trans. 726-728.

Malik GS, Singh SP (1978) J. Prakt. Chem. 320: 324.

Martell AE, Smith RM, Motekaitis RJ (2004) NIST Critically Selected Stability Constants of Metal Complexes Standard Reference Database 46, Version 8.0.

Matsui H, Ohtaki H (1982) A Potentiometric Study on Mixed Ligand Cadmium(II) Complexes with 2-Amino Carboxylic Acids. Bull. Chim. Soc. Jpn. 55: 461-465.

Meyer JL, Bauman JE (1970) Thermodynamics of amino acid-copper(II) complexes. J. Chem. Eng. Data 15: 404-407.

Nair MS, Santappa M (1981) Indian J. Chem. 20A: 990.

Nims LF, Smith PK (1933) The Ionization Of Dl-Alanine From Twenty To Forty-Five Degrees. J. Biol. Chem. 101: 401-412.

Nourmand M, Meissami N (1982) Complex Formation Between Uranium(VI) Ion and some α-Aminoacids. Polyhedron 1: 537-539.

Orenberg JB, Fischer BE, Sigel H (1980) Binary and ternary complexes of metal ions, nucleoside 5′-monophosphates, and amino acids. J. Inorg. Nucl. Chem. 42: 785-792.

Perrin DD (1958) 633. The stability of complexes of ferric ion and amino-acids. J. Chem. Soc. (Resumed) 3125-3128.

Petit-Ramel MM, Paris MR (1968) Bull. Soc. Chim. Fr. 2791.

Pettit LD (1984) Critical survey of formation constants of complexes of histidine, phenylalanine, tyrosine, L-DOPA and tryptophan. Pure Appl. Chem. 56: 247-292.

Pettit LD, Powell KJ (2001) IUPAC Stability Constants Database. Academic Software, IUPAC.

Prasad K, Mohan MS (1987) Studies On Biologically Relevant Binary And Ternary Metal Complexes. I. Ternary Cu(Ii) Complexes With Bipyridyl And Bidentate Amino Acids. J. Coord. Chem. 16: 1-7.

Ramanujam VV, Rengaraj K, Sivasankar B (1979) Studies on Uranyl Complexes. II. Unidentate Carboxylate Coordination in Uranyl Complexes of α-, β-, and γ-Amino Acids: A Polarographic Study. Bull. Chim. Soc. Jpn. 52: 2713-2716.

Ramanujam VV, Selvarajan VM (1981) J. Indian Chem. Soc. 58: 1131.

Rangaraj K, Ramanujam VV (1977) Stability constants of some uranyl complexes. J. Inorg. Nucl. Chem. 39: 489-491.

Sarin R, Munshi KN (1973) Stability constants and thermodynamic functions of indium(III) complexes with some organic acids from potentiometric data. J. Inorg. Nucl. Chem. 35: 201-207.

Sawhney MP, Joshi DP, Sharma KN, Jain PK (1980) Indian J. Chem. 19A: 85.

Scheidegger H, Felty W, Leussing DL (1970) Complexing of zinc(II) in the transamination system: .alpha.-oxoglutarate + .alpha.-alaninate.far..rar.glutamate + pyruvate. J. Am. Chem. Soc. 92: 808-810.

Sharma G, Tandon JP (1971) Potentiometric studies of ternary complex formation. Talanta 18: 1163-1167.

Sharma VS, Mathur HB (1965) Indian J. Chem. 3: 475.

Simeon VL, Weber OA Croat. Chim. Acta 38: 161.

Smith PK, Taylor AC, Smith ERB (1937) Thermodynamic Properties Of Solutions Of Amino Acids And Related Substances: Iii. The Ionization Of Aliphatic Amino Acids In Aqueous Solution From One To Fifty Degrees. J. Biol. Chem. 122: 109-123.

Sóvágó I, Kiss T, Gergely A (1993) Critical Survey Of The Stability Constants Of Complexes Of Aliphatic Amino Acids. Pure Appl. Chem. 65: 1029-1080.

Sóvágó I, Varnagy K, Bényei A (1986) Magy. Kém. Foly. 92: 114.

Sturtevant JM (1942) Calorimetric Investigations of Organic Reactions. IV. The Heats of Ionization of dl-Alanine at 25°. J. Am. Chem. Soc. 64: 762-768.

Vieles MMP, Bonniol A (1973) Compt. Rend. Acad. Sci.. Ser. C. 276: 1769.

Vlasova NN, Davidenko NK (1985) Zh. Neorg. Khim. 3: 1738.

Zhang XD, He M, Sun JY, Zhang F, Song XM, Liu QT (2000a) Studies on ligand-ligand interaction-formation and stability of proton-dipeptide-amino acid complexes. Acta. Chim. Sinica 58: 1641-1644.

Zhang XD, He M, Sun JY, Zhang F, Song XM, Liu QT (2000b) Study on the stability of Zn(II) - 12,Pentyl-1,4,7,10-tetraazacyclotridecane-11,13-dione - α-amino acids ternary complexes. Acta Chim. Sinica 58: 662-665.
